# Supplementary material for: Exploring ways to respond to rising obesity and diabetes in the Caribbean using a system dynamics model
Source: PLOS Glob Public Health. 2022 May 19;2(5):e0000436. doi: 10.1371/journal.pgph.0000436 (PMC10021196; doi:10.1371/journal.pgph.0000436)
Supplement: S1 File — (PDF) [file pgph.0000436.s001.pdf]

Systems Science for Caribbean Health: An obesity and diabetes simulation model for Jamaica

Supporting documentation

Leonor Guariguata[1], Leandro Garcia[2], Natasha Sobers[1], Trevor S Ferguson[3], James Woodcock[4], T. Alafia Samuels[5], Cornelia Guell[6], Nigel Unwin[1,4,6]

1 George Alleyne Chronic Disease Research Centre, University of the West Indies, Cave Hill, Barbados
2 Centre for Public Health, School of Medicine, Dentistry and Biomedical Sciences, Queen's University, Belfast, United Kingdom
3 Epidemiology Research Unit, Caribbean Institute for Health Research, University of the West Indies, Mona, Jamaica
4 MRC Epidemiology Unit, University of Cambridge School of Clinical Medicine, Cambridge, United Kingdom
5 Caribbean Institute for Health Research, University of the West Indies, Mona, Jamaica
6 European Centre for Environment and Human Health, University of Exeter Medical School, Cornwall, United Kingdom

Jan 18, 2022

Background

The Systems Science for Caribbean Health project uses a system dynamics approach to the development of a simulation model to estimate future trends in obesity and type 2 diabetes in Jamaica (which accounts for between 90 to 95% of all diabetes) as well as the potential effects of interventions. This model produces estimates for type 2 diabetes and not type 1 diabetes. For simplicity, the rest of the document refers to diabetes. The model is based on stakeholder-developed causal loop diagrams (CLDs) that map the feedback and pathways driving rising rates of diabetes and obesity in the Caribbean. Jamaica was chosen as a test case for developing the simulation model in part because of the availability of repeated surveillance data from the Jamaica Health and Lifestyle Surveys. This document presents the supporting information used in developing the model as well as the assumptions applied to the inputs. This documentation is following the general guidelines proposed by STRESS(1) on reporting for models, specifically for system dynamics models.

An online version of the model is available via Silico: https://silico.app/@lguariguata/diabetes-model?s=i8NOUzgpTHuxQIXmyqc-Qw

The model was developed using R with “deSolve” package. The Git repository is available at: https://github.com/leoguari/SSCH-Model

Background 2
1 Model Structure 7
1.1 Summary Stock and Flow diagram 7
1.2 Time scale 7
Figure S1.1 Summary stock and flow diagram of the core structure of the SSCH model 8
2 Population inputs 9
Figure S2.1 Estimates of adult population inflow by year, Jamaica 9
Proportion of population over and under 55 years 9
Figure S2.2 Proportion of adults over 55 years Jamaica 10
Table S2.1 Average age for adult men and women for the Jamaican population 10
2.1 Aging across the different stocks 11
2.3 Equations for estimating the proportion of each stock over the age of 55 11
Table S2.2 Variables for estimating over 55 fractions 12
3 All-cause mortality 13
3.1 Calculating all-cause mortality 13
Figure S3.1 Structure for estimating all-cause mortality 14
3.3 Equations for estimating all-cause mortality 16
Table S3.1 Variables for estimating all-cause mortality 17
3.4 Calibration of mortality estimates with other sources 18
Figure S3.2 Comparison of all-cause mortality for adults (20+ years) from World Population Prospects, IHME Global Burden of Disease, and the SSCH Model for Jamaica 18
4 Pre-diabetes and diabetes incidence 19
Table S4.1 Definitions used for prediabetes and diabetes 19
4.1 The relationship between prediabetes and diabetes prevalence 19
Table S4.2 Summary of the relationships of prediabetes prevalence to diabetes prevalence from objectively measured studies in populations relevant to the Caribbean 20
Table S4.3 Comparison of the effect of different diagnostic criteria on prediabetes prevalence(21) 21
4.2 Diabetes incidence 21
4.3 Estimating the onset of diabetes 21
Table S4.4 A selection of studies estimating diabetes incidence in people with prediabetes 22
4.4 Effect of aging on diabetes onset 22
4.5 Effect of obesity on diabetes onset 22

Table S4.5 Estimates of the relative risk of diabetes in the obese 22
4.6 Effect of physical activity on diabetes onset 23
4.7 Effects of sugar-sweetened beverage and fruit and vegetable consumption on diabetes onset 23
4.8 Estimating Obese fraction by glycemic status 23
4.8.1 Equations to estimate Obese fraction by glycemic status 23
Table S4.6 Obese fraction calculation variables 23
Figure S4.1 Structure for onset of diabetes 24
4.9 Equations to estimate diabetes onset rate 25
Table S4.7 Variables to estimate diabetes onset rate 26
4.10 Comparison of SSCH diabetes incidence estimate to GBD incidence estimate 27
Figure S4.2 Comparison of estimates for new cases of diabetes from SSCH model and the Global Burden of Disease for Jamaica 27
5 Pre-diabetes epidemiology 28
Table S5.1 Evidence in the literature for prediabetes incidence 28
5.1 Effect of aging on prediabetes onset 29
5.2 Effect of obesity on prediabetes onset 29
Table S5.2 Evidence of relative risk of prediabetes in the obese 29
5.3 Recovery from pre-DM 29
Table S5.3 Summary of studies with information on reversion rates 30
31
5.4 Equations to estimate the onset and recovery for prediabetes 31
Table S5.4 Variables to estimate the onset and recovery for prediabetes 32
6 Estimating obesity 33
6.1 Estimating Obesity from BMI 33
Figures S6.1 a, b, c Binomial curves to estimate the obesity prevalence from BMI taken from African and diaspora population 34
Figure S6.2 Structure for estimating BMI and obesity 35
6.2 Equations used to estimate the obesity prevalence 36
Table S6.1 Variables to estimate obesity and BMI 37
7 Physical Activity 38
Table S7.1 Evidence of Caribbean MVPA from STEPs Surveys(56) 38
7.1 Jamaican data on physical activity 38
Figure S7.1 Self-reported physical activity levels from the Jamaica Health and Lifestyle Survey 2007/2008(20) 39
Figure S7.2 Self-report PA (min/d, GPAQ) for occupation, travel and recreation PA, by site for men and women from the METS study(59) JA = Jamaica 40

7.2 Modeling Physical Activity data for Jamaica 40
7.3 Data inputs for physical activity time series 41
Figure S7.3 Modeled trends for the SSCH inputs for physical activity 42
Table S7.2 Time series inputs for MVPA by domain 42
Figure S7.4 Structure for estimating MVPA 43
7.4 Equations for estimating MVPA 44
Table S7.3 Variables for estimating MVPA 45
7.5 Physical Activity Level estimation 46
Table S7.4 Reference ranges for PAL 46
7.6 Estimating physical activity intensity 46
Table S7.5 MET values for typical activities 46
Table S7.6 MET assumption inputs for PA categories 47
7.7 Differences in physical activity levels between men and women 47
Figure S7.5 SSCH modeled PAL levels for men and women 47
Figure S7.6 Structure for calculating Physical Activity Level 48
7.8 Equations to estimate physical activity levels 49
Table S7.7 Variables to calculate physical activity level 50
8 Caloric intake 51
8.1 Estimating caloric intake for men and women 51
Table S8.1 Caloric intake studies relevant to Jamaica and the Caribbean 52
8.2 Sugar-sweetened beverage intake 52
Table S8.2 Sugar sweetened beverage intake time series inputs 52
8.3 Ultra-processed food consumption 52
Figures S8.1, a,b Average daily retail of ultra-processed products in Latin America(77) 53
Figure S8.2 SSCH modeled ultra-processed foods calories 54
Table S8.3 Studies reporting estimates of ultra-processed food consumption 55
Figure S8.3 SSCH estimates of proportion of total caloric intake from ultra-processed foods 55
Figure S8.4 SSCH total caloric intake estimates for men and women 56
Figure S8.5 Structure for estimating caloric intake 57
8.4 Equations to estimate caloric intake 58
Table S8.4 Variables to estimate caloric intake 59
9 Calibration and baseline model predictions 60
9.1 Diabetes prevalence 60

Figure S9.1 Estimated diabetes prevalence from the SSCH model compared to measured estimates from studies 60

Figure S9.2 Estimated obesity prevalence from the SSCH model compared to measured estimates from studies 61

Figure S9.3 Estimated BMI from the SSCH model compared to measured estimates from studies 61

10 Scenario testing 62

Table S10.1. Stakeholder-identified priority areas for intervention simulation 62

Table S10.2. A summary of literature-based scenarios for testing in the SSCH model and 30-year effects on diabetes and obesity prevalence 63

10.1 Magnitude change of changing individual inputs 64

Figure S10.1 a-i. Dose-response curves for magnitude changes in physical activity, diet, and health system interventions 65

10.2 Aspirational scenarios 66

Table S10.3 Aspirational scenarios and their impacts on diabetes and obesity prevalence 66

10.2 Achieving global targets 66

Figure S10.2 Impact of changes in caloric intake and MVPA on achieving global targets to stop the rise in diabetes and obesity prevalence in adults. 67

Figure S10.3 Analysis of global targets with no decreasing trend in MVPA or increasing trend in caloric intake 68

11 Sensitivity analyses 69

Table S11.1 Ranges for the sensitivity analyses 69

11.1 Multivariable sensitivity analysis of epidemiological assumptions 69

Figure S11.1 Multivariate sensitivity analyses effect on diabetes prevalence estimates70

70

11.2 Univariable sensitivity analysis of epidemiological assumptions 70

Figures S11.2a-o Univariate sensitivity analyses effect on diabetes prevalence 71

References 73

1 Model Structure

The model can be summarized by the diagram below which shows a stock and flow structure.

1.1 Summary Stock and Flow diagram

The diagram is composed of a core structure for the epidemiological model of diabetes progression. There are three main stocks represented by the boxes for normoglycemic population (NGT), prediabetes population (preDM), and diabetes population (DM). Added up, these account for the whole of the adult population with the underlying assumption that there is no overlap between these groups. People may move from a normoglycemic state, to prediabetes and from prediabetes back to normoglycemia. Those who have prediabetes may also move to diabetes, but this flow moves only in one direction. A few clinical trials in controlled conditions have shown that it is possible for some people with type 2 diabetes to revert to a prediabetes or normoglycemic state(2). However, this reversal has not been demonstrated at the population level and for the purposes of this model is not considered.

Stocks are connected by flows which determine the accumulated values in each stock over time. The flows connecting each of the stocks are described below.

Normoglycemic population

- Inflows: population growth(3); reversion of people with prediabetes back to normoglycemia
- Outflows: all-cause mortality in people with normoglycemia; progression from normoglycemia to prediabetes

Prediabetes population

- Inflows: people converting from normoglycemia to prediabetes
- Outflows: people reverting from prediabetes to normoglycemia; all-cause mortality in people with prediabetes; conversion of prediabetes to diabetes

Diabetes population

- Inflows: people converting from prediabetes to diabetes
- Outflows: all-cause mortality in people with diabetes

These flows are themselves modified by other factors, such as aging of the population, the population prevalence of obesity and determinants further upstream like caloric intake, dietary patterns, and physical activity patterns. These will be discussed in detail.

1.2 Time scale

The model was constructed from 1990 and projected to 2050 with a time step of one year.

Figure S1.1 Summary stock and flow diagram of the core structure of the SSCH model

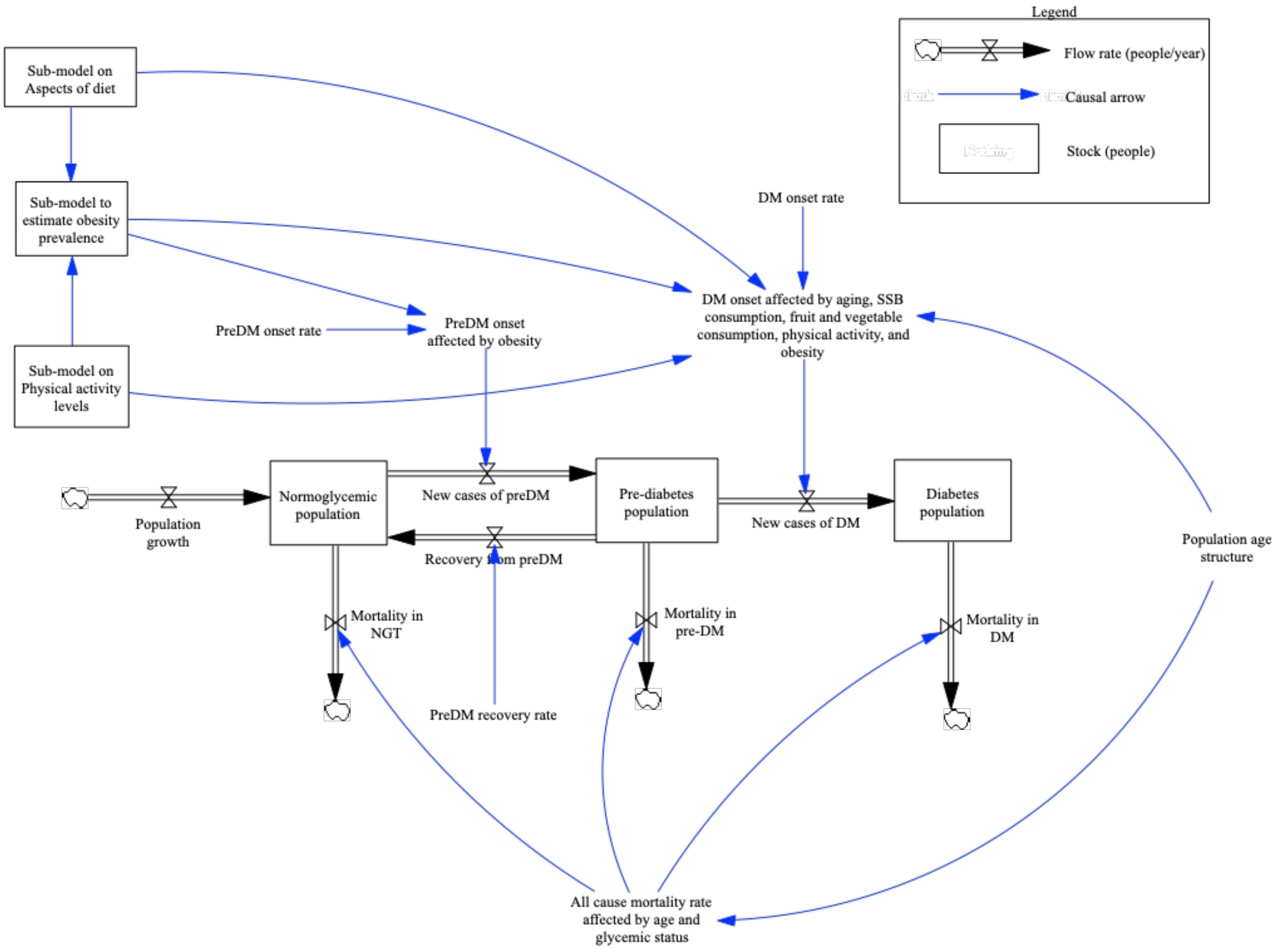

2 Population inputs

The population considered for the model is adults (20+ years) in Jamaica. Population estimates were derived from the World Population Prospects (2019 Revision)(3). Annualized age- and sex-specific data were downloaded for adults in Jamaica from 1990 to 2019 and projections from 2020 to 2050. Total deaths by age and sex were also downloaded for the same years and projections for use in validation and to estimate inflows to the population. These were used to estimate the total inflow to the adult population using the following equation:

Annual Inflow of adults = ΔTotal population$_{(yi+1-yi)}$ + Deaths$_{yi}$

*Where y_i is a given year

Figure S2.1 Estimates of adult population inflow by year, Jamaica

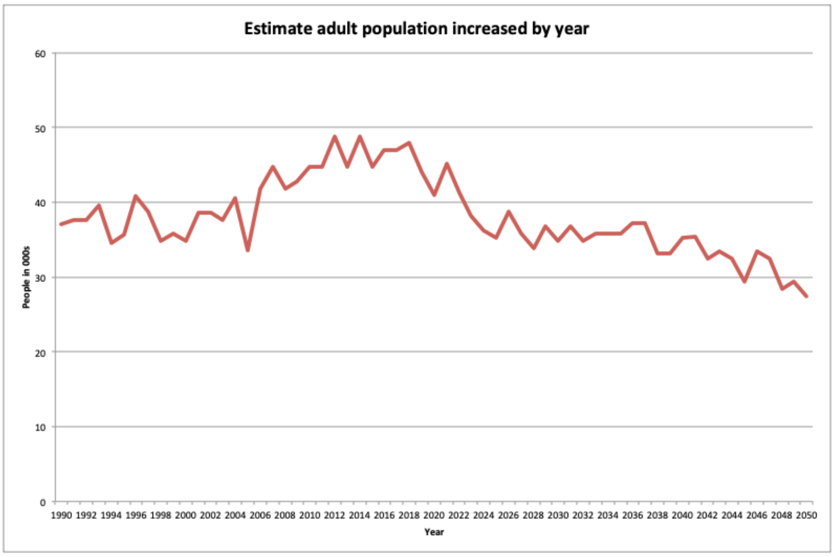

Proportion of population over and under 55 years

Aging is an important risk factor related to diabetes. It may be of interest in the future to subdivide the populations presented here by age group to explore the effects of changes in population age structure on diabetes prevalence. However, system dynamics modeling favors models that can reproduce historical data with as simple a structure as possible. We followed the suggestions by Jones et al(4) in developing a similar model of diabetes epidemiology in the United States and used proportions of the population in different age groups, combined with relative risk estimates for mortality and diabetes incidence to adjust for changing age structures. The World Population Prospects(3) data were used, again, to generate estimates of the proportion of the adult population 55 years and over, and between 20 and 54 years to be able to implement the calculations. The curve shows an overall aging of the population and this is taken into account in the estimates.

Figure S2.2 Proportion of adults over 55 years Jamaica

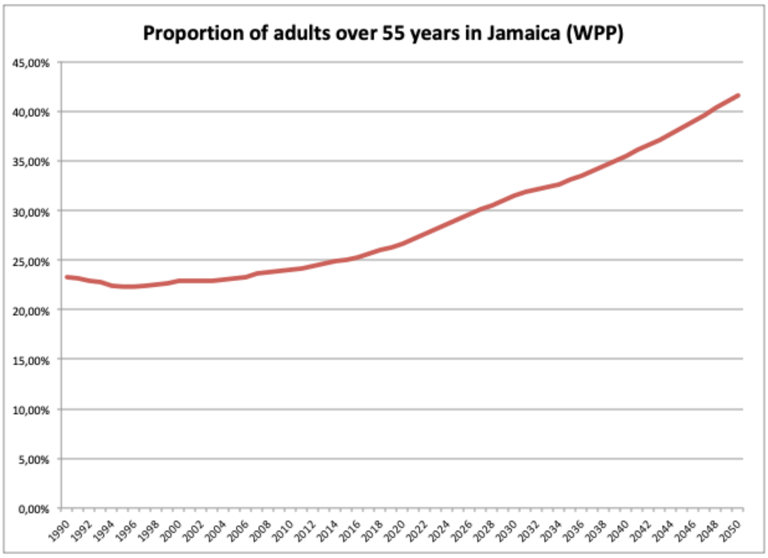

Similarly, the average age for men and women from 1990 and 2050 was calculated using WPP estimates. These are necessary for the estimation of basal metabolic rate.

Table S2.1 Average age for adult men and women for the Jamaican population

Year	Female average age	Male average age
1990	41.5	40.7
1995	41.6	41.0
2000	42.2	41.9
2005	43.0	42.5
2010	43.3	42.8
2015	43.5	42.9
2020	44.1	43.4
2025	45.2	44.4
2030	46.5	45.5
2035	47.7	46.5
2040	48.8	47.5
2045	49.9	48.5
2050	51.0	49.4

2.1 Aging across the different stocks

Age is a risk factor for prediabetes and diabetes. As a result, the age structures of the three stocks are different from each other with people with prediabetes being on average older than those who are normoglycemic, and people with diabetes being older than those with prediabetes. This effect of aging affects the incidence of prediabetes and diabetes, as well as the risk of death for each condition.

We used a weighted average that included the relative risk of prediabetes and diabetes to algebraically estimate the fraction of people over the age of 55 for each stock.

2.3 Equations for estimating the proportion of each stock over the age of 55

$$FracNG_{\geq 55} = FracTotalPop_{\geq 55} / (NGprev/100 + PreDMprev/100*RRPreDM_{\geq 55} + DMprev/100 * RRDM_{\geq 55})$$

$$FracPreDM_{\geq 55} = (FracNG_{\geq 55} * RRPreDM_{\geq 55})*100 / ((RRPreDM_{\geq 55}*FracNG_{\geq 55} + (100 - FracNG_{\geq 55}))$$

$$FracDM_{\geq 55} = (FracPreDM_{\geq 55}*RRDM_{\geq 55})*100 / ((RRDM_{\geq 55}*FracPreDM_{\geq 55} + (100 - FracPreDM_{\geq 55}))$$

$$FracTotalPop_{<55} = 100 - FracTotalPop_{\geq 55}$$

$$FracNG_{<55} = 100 - FracNG_{\geq 55}$$

$$FracPreDM_{<55} = 100 - FracPreDM_{\geq 55}$$

$$FracDM_{<55} = 100 - FracDM_{\geq 55}$$

Table S2.2 Variables for estimating over 55 fractions

Variable	Units	Description
$FracTotalPop_{\geq 55}$	dimensionless	Proportion of the total adult population over 55 years from the World Population Prospects(2)
$FracTotalPop_{<55}$	dimensionless	Proportion of the total adult population under 55 years
$FracNG_{\geq 55}$	dimensionless	Fraction of people over 55 in the normoglycemic population
$FracPreDM_{\geq 55}$	dimensionless	Fraction of people over 55 in the prediabetes population
$FracDM_{\geq 55}$	dimensionless	Fraction of people over 55 in the diabetes population
$FracNG_{<55}$	dimensionless	Fraction of people under 55 in the normoglycemic population
$FracPreDM_{<55}$	dimensionless	Fraction of people under 55 in the prediabetes population
$FracDM_{<55}$	dimensionless	Fraction of people under 55 in the diabetes population
NGPrev	dimensionless	Fraction of population that is normoglycemic
PreDMPrev	dimensionless	Fraction of population that has prediabetes
DMPrev	dimensionless	Fraction of population that has diabetes
$RRPreDM_{\geq 55}$	dimensionless	Relative risk of prediabetes in people over 55 years; set at 1.2
$RRDM_{\geq 55}$	dimensionless	Relative risk of diabetes in people over 55 years; set at 2.4

3 All-cause mortality

The mortality rate for each of the different stocks (NG, preDM, DM) was calculated using a baseline estimate for all-cause mortality for adults under and over 55 years. This estimate for mortality was extracted from the Global Burden of Disease study (GBD) for Jamaica(5). We obtained annual total all-cause mortality rates from the Global Burden of Disease Study for adults in Jamaica from 20 - 54 years, and 55 years and older. The mortality rates from 1990 to 2019 estimated by the GBD do not change substantially over time, so an average of the annual rates is used as a constant. These are as follows:

Total all-cause mortality (deaths per 1000) in adults 20 - 54 years: 2.09
Total all-cause mortality (deaths per 1000) in adults 55 plus years: 31.96

There are differences in mortality by sex but these were not taken into account at this point because the stocks themselves are not separated by sex and it does not add to the precision of the estimates.

3.1 Calculating all-cause mortality

The total all-cause mortality (ACM) rate is a weighted average of the different rates in the sub-populations by glycemic status. This can be represented by the equation:

Total ACM rate = (ACM rate in NG * NG population + ACM rate in preDM * preDM population + ACM rate in DM * DM population)/Total population

These rates can be further subdivided by age groups by using the estimated proportion of people in each age group by glycemic status as described above.

The total all-cause mortality rate for the population is a weighted average of underlying mortality rates for each of the stocks (normoglycemic, prediabetes and diabetes). Tang et al show that the rates of all-cause mortality in normoglycemic people and people with prediabetes are similar and thus the rate in these two stocks is assumed to be the same.

The equation can be rewritten as:

Total ACM rate in age group = [ACM rate in NG-preDM in age group * (NG population in age group + preDM population in age group) + ACM rate in NG-preDM in age group * RR of ACM in DM in age group * DM population in age group] / Total population in age group

This can be written alternatively using the diabetes prevalence (%) as:

Total ACM rate in age group = ACM rate in NG-preDM in age group * (1 - DM prev) + ACM rate in NG-preDM in age group * RR of ACM in DM in age group * DM prev

Figure S3.1 Structure for estimating all-cause mortality

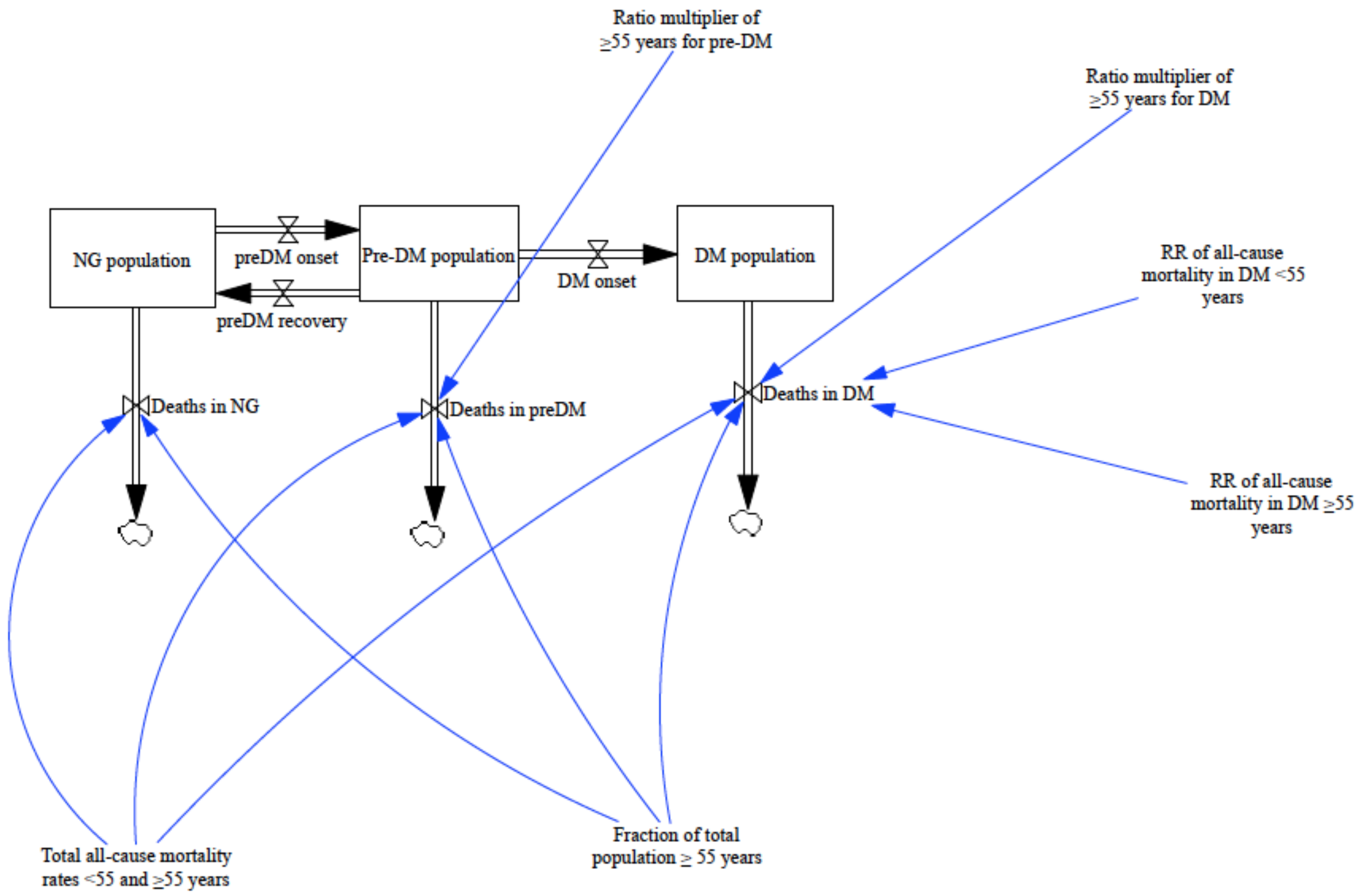

For diabetes, the rate of all-cause mortality is higher and varies by age. We used estimates from Roglic and Unwin(6) and from African American populations in a national cohort of the US(7) to determine a relative risk of all-cause mortality in adults with diabetes from 20 - 54 years and 55 years and over.

The values used for the SSCH model from these sources are:
Relative risk (RR) of all-cause mortality in people with diabetes under 55 years = 3.5
Relative risk (RR) of all-cause mortality in people with diabetes over 55 years = 1.6

These are assumed to be constant over time based on evidence from the US NHANES study showing that even though the underlying mortality rate changes over time, the relative risk remains stable. Despite there being evidence that mortality rates are decreasing in some parts of the world(8,9), and especially in people with diabetes, this does not seem to be true of the Caribbean where mortality rates have remained relatively constant(10,11).

We can then separate the equations out for the two glycemic status groups to determine the ACM rates for each group by rearranging the equation above and applying the assumptions for total ACM and the RR of ACM in the DM population.

3.3 Equations for estimating all-cause mortality

ACM rate in NG-preDM populations
20 to 54 years

$$\text{ACM NG-preDM}_{<55} = 2.09 / [(1 - \text{DMprev}_{<55}) + 1.6 * \text{DMprev}_{<55})]$$

where

$$\text{DMprev}_{<55} = (\text{DMPop} * (100\text{- FracDM}_{\geq 55})*100 / (\text{TotalPop} * (100\text{-FracTotalPop}_{\leq 55}))$$

55 years and older

$$\text{ACM NG-preDM}_{\geq 55} = 31.96 / [(1 - \text{DMprev}_{\geq 55}) + 3.5 * \text{DMprev}_{\geq 55})]$$

where

$$\text{DMprev}_{\geq 55} = (\text{DMPop} * \text{FracDM}_{\geq 55}) / (\text{TotalPop} * \text{FracTotalPop}_{\geq 55})$$

ACM rate in DM population

$$\text{ACM for DM}_{<55} = \text{ACM for NG-preDM}_{<55} * 3.5$$

$$\text{ACM for DM}_{\geq 55} = \text{ACM for NG-preDM}_{\geq 55} * 1.6$$

Calculating deaths per year

$$\text{DeathsNG} = (\text{ACM NG-preDM}_{<55} * \text{NGPop} * \text{FracTotalPop}_{<55}) + (\text{ACM NG-preDM}_{\geq 55} * \text{NGPop} * \text{FracTotalPop}_{\geq 55})$$

$$\text{DeathsPreDM} = (\text{ACM NG-preDM}_{<55} * \text{PreDMPop} * \text{FracPreDM}_{<55}) + (\text{ACM NG-preDM}_{\geq 55} * \text{PreDMPop} * \text{FracPreDM}_{\geq 55})$$

where

$$\text{FracPreDM}_{\geq 55} = \text{FracPreDMPop}_{\geq 55} * \text{RatioPreDM}_{\geq 55}$$

$$\text{FracPreDM}_{<55} = 1 - \text{FracPreDM}_{\geq 55}$$

$$\text{DeathsDM} = (\text{ACM for DM}_{<55} * \text{DMPop} * \text{FracDM}_{<55}) + (\text{ACM for DM}_{\geq 55} * \text{DMPop} * \text{FracDM}_{\geq 55})$$

Table S3.1 Variables for estimating all-cause mortality

Name	Units	Description
ACM NG-preDM$_{<55}$	per 1000 people per year	All-cause mortality rate in normoglycemic and prediabetes adults 20 to 54 years
DMprev$_{<55}$	dimensionless	The fraction of people with diabetes under 55 years over the total population under 55 years
FracDM$_{\geq 55}$	dimensionless	Fraction of the diabetes population (stock) over 55 years
FracTotalPop$_{\geq 55}$	dimensionless	Fraction of the total population (sum of NG, preDM, and DM stocks) under 55 years
FracDM$_{<55}$	dimensionless	The fraction of the diabetes population (stock) under 55 years
TotalPop	People in 1000s	The sum of the stocks for NG, preDM and DM
FracTotalPop$_{<55}$	dimensionless	The population under 55 years taken from estimates from the World Population Prospects (described above)
ACM NG-preDM$_{\geq 55}$	per 1000 people per year	All-cause mortality rate in normoglycemic and prediabetes adults 55 years and older
DMprev$_{\geq 55}$	dimensionless	Fraction of people with diabetes over 55 years over the total population over 55 years
ACM for DM$_{<55}$	per 1000 people per year	All-cause mortality rate in adults with diabetes under 55 years
ACM for DM$_{\geq 55}$	per 1000 people per year	All-cause mortality rate in adults with diabetes 55 years and older
DeathsNG	People in 1000s	Number of deaths in normoglycemic people (stock)
NGPop	People in 1000s	Normoglycemic population (stock)
DeathsPreDM	People in 1000s	Number of deaths in prediabetes people (stock)
PreDMPop	People in 1000s	Pre-diabetes population (stock)
FracPreDM$_{\geq 55}$	dimensionless	Fraction of the prediabetes population (stock) over 55 years
FracPreDM$_{<55}$	dimensionless	Fraction of the prediabetes population (stock) under 55 years
DeathsDM	People in 1000s	Number of deaths in diabetes people (stock)
DMPop	People in 1000s	Diabetes population (stock)
ACMTotal$_{<55}$	per 1000 people per year	All-cause mortality estimate for the whole population under 55 years taken from GBD study set at 2.09
ACMTotal$_{\geq 55}$	per 1000 people per year	All-cause mortality estimate for the whole population over 55 years taken from GBD study set at 31.96
RR ACM DM$_{<55}$	dimensionless	Relative risk of all-cause mortality in people with diabetes under 55 years; set at 3.5
RR ACM DM$_{\geq 55}$	dimensionless	Relative risk of all-cause mortality in people with diabetes over 55 years; set at 1.65

3.4 Calibration of mortality estimates with other sources

Validation of overall deaths compared the estimates from GBD and estimates derived from WPP. The WPP only provides death estimates in adults by 5 year age bands. An average imputation was used following a simple trendline. GBD/IHME does not provide a projection for deaths going forward. The SSCH estimates are slightly higher than those from WPP. The effect of any overestimate in projected deaths from the SSCH model on the outcomes of interest would mostly affect the prevalence of diabetes and cause the incidence of diabetes to be underestimated. We prefer to adopt a conservative approach to our key measures of interest (prevalence, incidence, and obesity prevalence) and thus will not adjust further for mortality.

Figure S3.2 Comparison of all-cause mortality for adults (20+ years) from World Population Prospects, IHME Global Burden of Disease, and the SSCH Model for Jamaica

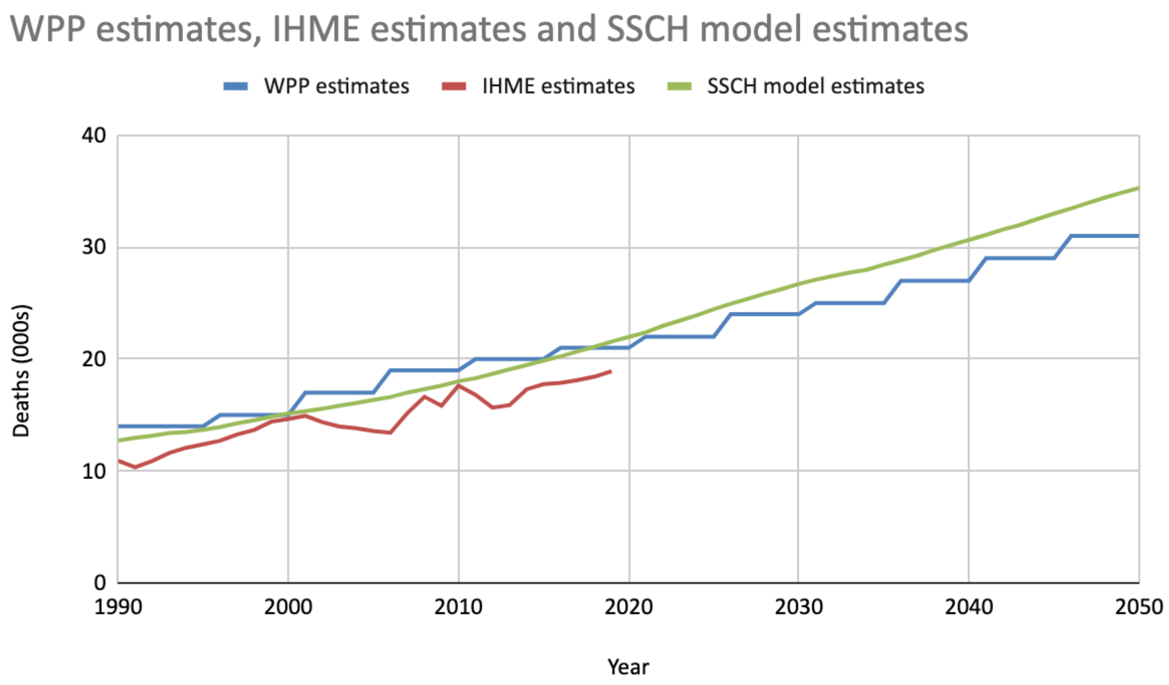

4 Pre-diabetes and diabetes incidence

There are many diagnostic criteria for defining prediabetes and diabetes and the definition used has an impact in determining the initial proportions to establish the sizes of the stocks. A summary of diagnostic criteria currently being used and their cut points are listed in the table below.

Table S4.1 Definitions used for prediabetes and diabetes

**Prediabetes**			
Criterion	Glucose Tolerance	Fasting Glucose	HbA1c
WHO 2006(12)	2 h plasma glucose 7.8 - 11.0 mmol/L (140 - 200 mg/dL) after 75g OGTT	6.1 - 6.9 mmol/L (110 - 125 mg/dL)	NA
ADA 2010(13)	2 h plasma glucose 7.8 - 11.0 mmol/L (140 - 200 mg/dL) after 75g OGTT	5.6 - 6.9 mmol/L (100 - 125 mg/dL)	5.7 - 6.4%
**Diabetes**			
WHO 2003 and ADA 2010	2h plasma glucose ≥ 11.1 mmol/L (200 mg/dL)<br>Or<br>(random plasma glucose ≥ 11.1 mmol/L (200mg/dL)	≥ 7 mmol/L (126 mg/dL)	≥ 6.5%

In order to simulate the estimated sizes of each of the stock populations (NG, preDM, DM) we need to estimate their initial sizes. We assume that prediabetes prevalence is roughly double that of diabetes prevalence using the American Diabetes Association definition of a fasting blood glucose of ≥5.6 mmol/L and <7.0 mmol/L or an HbA1c of 5.7 - 6.4 % or post oral glucose tolerance 2 hour result of 7.8 - 11.1 mmol/L. A different, more limited definition of diabetes would result in a different ratio as fewer people would then be diagnosed as “pre-diabetic” in various forms (impaired fasting glucose, raised HbA1c, impaired glucose tolerance or some combination of these).

4.1 The relationship between prediabetes and diabetes prevalence

A number of studies with objectively measured diabetes that show the relationship between prediabetes categories and diabetes in relevant populations. A summary is presented in Table 4.2.

Table S4.2 Summary of the relationships of prediabetes prevalence to diabetes prevalence from objectively measured studies in populations relevant to the Caribbean

Country	Pre-diabetes prevalence	Diabetes prevalence	Ratio	Notes on criteria
British Virgin Islands(14)	25.7	8.8	2.92	plasma (≥6.1 mmol/L and <7 or capillary ≥5.6 and <6.1mmol/L)
Dominica(15)	28.2	17.7	1.59	plasma (≥6.1 mmol/L and <7 or capillary ≥5.6 and <6.1mmol/L)
Belize(16)	16.5	12.9	1.28	venous plasma sample 100 - 125 mg/dL for IFG
Jamaica(17)	12	10.2	1.17	Isolated IFG using ADA definition ≥5.6 - <7.0 mmol/L
Barbados(18)	44.1	20.3	2.17	ADA definition ≥5.6 and <7.0 mmol/L and HbA1c - broad definition
US Total population(19)	38	14.3	2.66	ADA definition ≥5.6 and <7.0 mmol/L and HbA1c
US subset Black population(19)	39.6	21.8	1.82	ADA definition ≥5.6 and <7.0 mmol/L and HbA1c
		Total average	1.94	
		Average without JHLS	2.07	

The initial diabetes prevalence was set as 4.5% of the total population and prediabetes as 7.0%. This was based on calibration of the model with existing data from the Jamaica Health and Lifestyle Surveys. The JHLS II (2007-2008) Survey(20) used the 2006 WHO definition of diabetes and reported a prevalence of 7.9% of adults 15-74 years. The survey reported a prevalence or 2.8% of impaired fasting glucose. If the diabetes epidemic is accelerating, as is the case in Jamaica, the difference between diabetes and prediabetes prevalence over time will decrease as the susceptible population for diabetes decreases. However, much depends on the definition of diabetes and prediabetes used.

Evidence from a review done by the European Diabetes Epidemiology Group(21) comparing different cut points suggests that the prevalence of IFG as defined by the ADA criteria was a median 3.2 times higher than that using the WHO criteria (Table 4.3).

This would suggest that the JHLS II results would be closer to a 9% prevalence of prediabetes for that age group. Nevertheless, given the uncertainty around measuring impaired glycemic states, the variety of diagnostic criteria and their relationships with outcomes this particular stock is used more as an intermediate step in the model and will not be considered for calibration of other variables or as a primary outcome of interest. Definitions of diabetes have less variability and so the onset of diabetes from a pre-diabetic state, or diabetes incidence, is easier to calibrate given the limited data for this population.

Table S4.3 Comparison of the effect of different diagnostic criteria on prediabetes prevalence(21)

Country	WHO criteria	ADA criteria	Ratio	Source
US	6.7	24.1	3.6	NHANES(22)
Denmark	11.8	37.6	3.2	Inter99(23)
France	15.9	45.2	2.8	DETECT-2(23)
China	11.2	26.7	2.4	DETECT-2(23)
India	10.6	37.6	3.5	DETECT-2(23)
US	9.2	28.5	3.1	EDEG(21)
Singapore	9.5	32.3	3.4	Singapore CVD Cohort Study(22)
		median	3.2	

4.2 Diabetes incidence

The estimation of prevalence for both pre-DM and DM depends on a baseline incidence rate that then is adjusted for the effects of obesity, aging, and other interventions related to diet and physical activity. The assumption is that diabetes progression occurs as a stage following prediabetes. Thus, the baseline incidence is estimated in people without obesity but with pre-DM and this is adjusted for proportion of the pre-DM population with obesity and a relative risk for diabetes incidence in the obese.

Two systematic reviews by Gerstein et al. (24) and Xu et al. (25) more recently of the differences in incidence rates for diabetes from normoglycemic and hyperglycemic populations show wide variation in the incidence rates among those with prediabetes, in part due to differences in the diagnostic criteria used. Gerstein et al report annualized incidence rates of diabetes in individuals with impaired glucose tolerance (IGT) from 1.8 to 16.8% per year; isolated IGT from 4.4 to 6.4%; impaired fasting glucose from 1.6 to 34%; isolated impaired fasting glucose from 6.1 to 9.2% and highest among those with both impaired fasting and impaired glucose tolerance from 10 to 15% per year. The pooled relative risk of diabetes in those with different types of prediabetes ranged from 4.66 in those with IFG to 12.13 of those with both IFG and IGT compared to rates in the normoglycemic. Xu et al show a similar variation in the annualized incidence rate in people with prediabetes from 2.20 - 212.15 per 1000 person-years with differences by diagnostic criteria. For the studies using a lower cut-off for prediabetes definition the rates vary from 16.85 to 64.68 (Table 4.4).

We use a baseline rate of 50 cases per 1000 person-years in people with prediabetes. This value most closely calibrates to the diabetes prevalence estimates we have from repeated surveys in Jamaica and fits within the range of the reported incidence rates in similar populations. This baseline rate is held constant throughout the model and only the effects of changes in obesity, age structure, and in health determinants are then included.

4.3 Estimating the onset of diabetes

The baseline rate of diabetes incidence is influenced by the underlying obesity prevalence, aging structure over time of the population, and other factors like sugar sweetened beverage consumption(26), leisure time physical activity(27), and fruit and vegetable consumption(26).

Each of these effects is assumed to be independent of the others. While there are many other risk factors that can influence the incidence of diabetes, the one included in the model were those which most closely aligned with the priorities set for policy interventions in consultation with key stakeholders in the region(28,29).

Table S4.4 A selection of studies estimating diabetes incidence in people with prediabetes

Incidence rate (per 1000 person-years) for people with preDM	Country	Mean age	Year	Diagnostic criteria
36.66	United States	76.5	1997	ADA 2010
50.69	Mexico	45.5	2001	ADA 2007
58.33	Brazil	62	2002	ADA 2003
45.4	United States	55.04	1991	ADA 2003
53	United States	61	1983	ADA 1997

4.4 Effect of aging on diabetes onset

This variable uses a weighted average of the fraction of the population over 55 across each of the stocks and depends on the relative risk of diabetes in those over 55. The average relative risk of diabetes in those over 55 years is set at 2.44 following the effect size for age over 55 years from the Diabetes Risk Score(30).

4.5 Effect of obesity on diabetes onset

This variable uses a weighted average of the fraction of obesity across each of the stocks and depends on the relative risk of diabetes in the obese. The average relative risk of diabetes in the obese is set at 3.9 following these estimates and similar reported estimates(31) (Table 4.5).

Table S4.5 Estimates of the relative risk of diabetes in the obese

Study	Country	Ratio	Notes
US SUPREME-DM(32)	United States	3.4	Observational cohort of 7 million insured US adults
NYC Community Cohort(33)	United States	3	Self-reported cohort of adults in NYC Community Health Survey
InterAct Study(34)	United Kingdom	3.5	Cohort study including longitudinal data
CAMDI Survey from Central America(16)			
	Belize	2.47	Large, objectively measured cross-sectional study of diabetes in Central America. Population-based among adults.
	San José, Costa Rica	4.13	
	San Salvador, El Salvador	4.8	
	Guatemala City	3.7	
	Tegucigalpa	6.1	
	Managua, Nicaragua	4.45	
	Overall for the study	4.5	
	Average	3.95	

4.6 Effect of physical activity on diabetes onset

There are a number of studies showing an effect of physical activity on the incidence of diabetes, especially for those who are obese or have some form of prediabetes. There are many ways to implement an effect size on the overall incidence rate, in particular with respect to interventions in physical activity. We apply a dose-response curve that is dependent on estimated levels of MVPA of RR 0.87 for increments of 10-MET hours per week from a meta-analysis of prospective cohort studies by Smith et al.(27)

4.7 Effects of sugar-sweetened beverage and fruit and vegetable consumption on diabetes onset

The effects of SSB consumption and fruit and vegetable consumption are applied in a similar way to the other effects described above. Each varies the relative risk of diabetes using data drawn from systematic reviews. For SSB consumption, the effect of a unit increase in SSB consumption is taken from a systematic review and meta-analysis by Neuenschwander et al(26) that shows that a one serving per day (250 mL) increase in consumption leads to a 26% increase in incidence. A similar dose-response relationship was described by the same study for total fruit and total vegetable intake where each additional 100g of either per day led to a 0.02 reduction in incidence. The study does not report a combined measure for fruits and vegetables, so in the interest of being conservative in the evidence, we use the same curve for a combined fruit and vegetable intake. In other words: a 100g per day increase in fruit and vegetable consumption leads to a 2% reduction in diabetes incidence.

4.8 Estimating Obese fraction by glycemic status

A number of calculations depend on the estimate of the obese fraction in each of the stocks. This is done using a simple weighted average equation that depends on estimates of the total obesity prevalence which is described in detail below. The equations to establish those fractions are presented here.

4.8.1 Equations to estimate Obese fraction by glycemic status

$$ObeseFracNG = ObesityPrev / (NGprev/100 + PreDMprev/100*RRPreDM_{Obese} + DMprev/100 * RRDM_{Obese})$$

$$ObeseFracPreDM = (ObeseFracNG*RRPreDM_{Obese})*100 / ((RRPreDM_{Obese}*ObeseFracNG + (100 - ObeseFracNG))$$

$$ObeseFracDM = (ObeseFracPreDM*RRDM_{Obese})*100 / ((RRDM_{Obese}*ObeseFracPreDM + (100 - ObeseFracPreDM))$$

Table S4.6 Obese fraction calculation variables

Variable	Units	Description
ObesityPrev	dimensionless	Prevalence (%) of obesity in the total population (full accounting of calculations is in the section on obesity below)
ObeseFracNG	dimensionless	Fraction of obesity in the Normoglycemic population
ObeseFracPreDM	dimensionless	Fraction of obesity in the prediabetes population
ObeseFracDM	dimensionless	Fraction of obesity in the Diabetes population

Figure S4.1 Structure for onset of diabetes

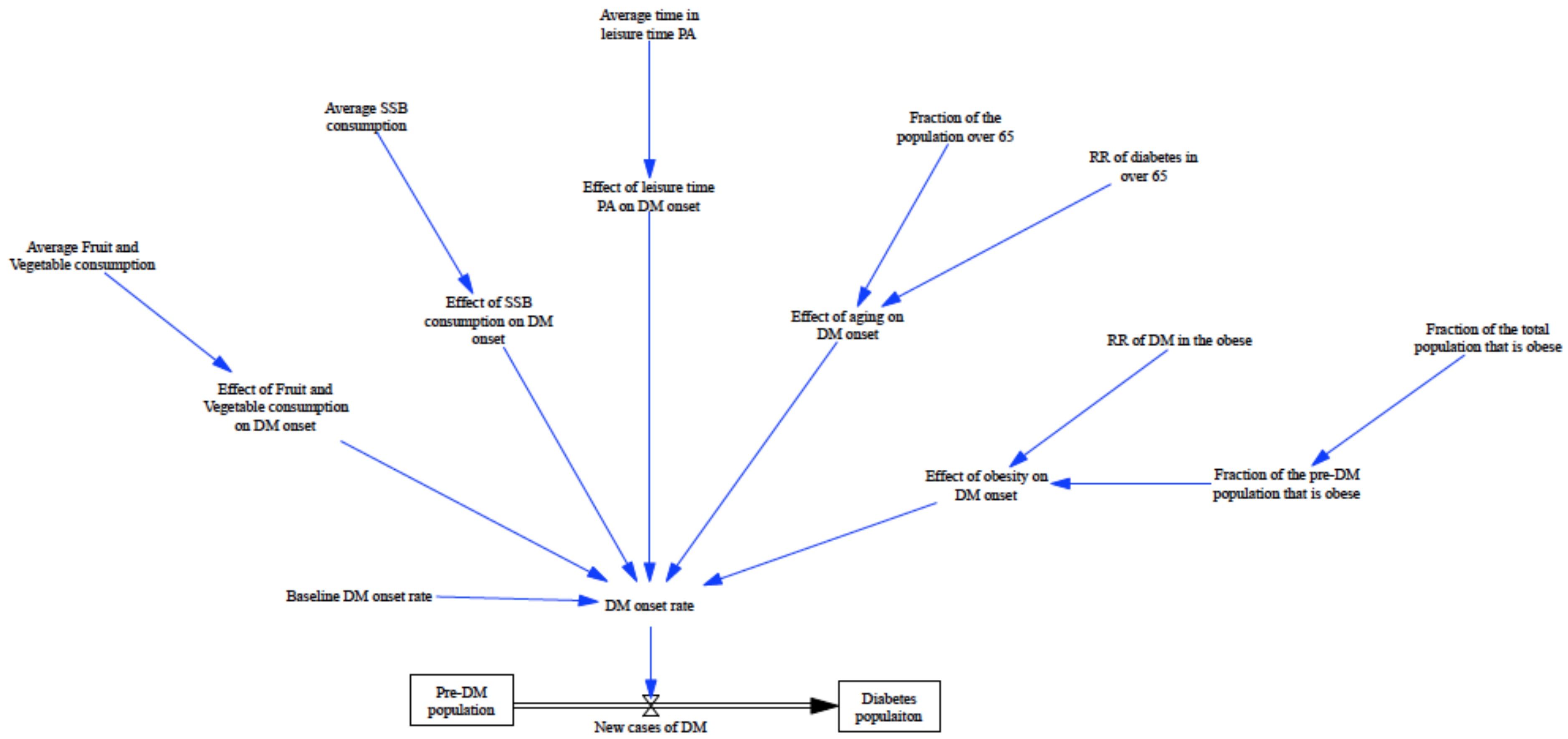

4.9 Equations to estimate diabetes onset rate

$$EffectObesity_{DM} = ((1 - ObeseFracPreDM) + (ObeseFracPreDM)*RRDM_{Obese}) / ((1 - ObeseFracPreDM_{Init}) + (ObeseFracPreDM_{Init}) * RRDM_{Obese}) /$$

$$EffectAging_{DM} =((1 - FracOver55_{PreDM}) + (FracOver55_{PreDM})*RRDM_{Aging}) / ((1 - FracOver55_{PreDMInit}) + (FracOver55_{PreDMInit}) * RRDM_{Aging})$$

$$EffectPA_{DM} = 1-(METs/10 - METs_{Ini}/10_{t}) * RR_{PA}$$

$$EffectFV_{DM} = 1 - (FVConsumption - FVConsumption_{Init}) / 200 * RR_{FV}$$

$$EffectSSB_{DM} = 1 + (SSBUnits - SSBUnits_{Init}) * RR_{SSBs}$$

$$DMOnsetRate_{Adjusted} = DMOnsetRate_{Base} * EffectObesity_{DM} * EffectAging_{DM} * EffectPA_{DM} * EffectFV_{DM} * EffectSSB_{DM}$$

$$DMOnset_{Cases} = PreDMPop * DMOnsetRate_{Adjusted}/1000$$

This final variable estimates the number of cases of DM in the population as a sum of the independent effects of obesity, physical activity, sugar-sweetened beverage consumption, fruit and vegetable consumption, and aging. This allows us then to estimate the impact of changing the patterns of any of these determinants on the incidence of diabetes in the population.

Table S4.7 Variables to estimate diabetes onset rate

Variable	Units	Description
ObesityPrev	dimensionless	Prevalence (%) of obesity in the total population
ObeseFracPreDM	dimensionless	Fraction of obesity in the pre-DM population; assumed to be 1.2 times the rate of obesity in the general population
$ObeseFracPreDM_{Init}$	dimensionless	The initial fraction of obesity in the preDM population; set at 24%
$EffectObesity_{DM}$	dimensionless	Effect of obesity on DM onset
$RRDM_{Obese}$	dimensionless	Relative risk of diabetes in the obese, constant set at 3.9
$EffectAging_{DM}$	dimensionless	Effect of aging on DM onset
$FracOver55_{PreDM}$	dimensionless	Fraction of the preDM population over 55 years taken from the World Population Prospects multiplied by 1.5
$FracOver55_{PreDMInit}$	dimensionless	Initial fraction of the preDM population over 55 years; set at 34%
$RRDM_{Aging}$	dimensionless	Relative risk of diabetes in the over 55, constant set at 2.4
$EffectPA_{DM}$	dimensionless	Effect of leisure time physical activity on DM onset
METs	MET hours per week	Average total MET-hours per week for the whole population
$METs_{Init}$	MET hours per week	Initial Average total MET-hours per week for the whole population
RR_{PA}	dimensionless	Decrease in incidence of diabetes from 10 METh/wk increase set at 0.87 or 13% from Smith et al (27)
$EffectFV_{DM}$	dimensionless	Effect of fruit and vegetable intake on DM onset
FVConsumption	Grams per day	Fruit and vegetable consumption estimated for the population in grams per day
$FVConsumption_{Init}$	Grams per day	Initial Fruit and vegetable consumption in grams per day
RR_{FV}	dimensionless	Effect on onset of consuming fruits and vegetables. Per every 200g consumed per day, there is a 0.02 fraction reduction in new cases
$EffectSSB_{DM}$	dimensionless	Effect of sugar-sweetened beverage consumption on DM onset
SSBUnits	Serving of sugar-sweetened beverages (250g)	Average servings of sugar-sweetened beverages consumed by the population
$SSBUnits_{Init}$	Serving of sugar-sweetened beverages (250g)	Initial servings of sugar-sweetened beverages

RR_{SSBs}	dimensionless	Added risk of a per unit increase in sugar sweetened beverage consumption. Set at 26% increase for every unit consumed.
$DMOnsetRate_{Adjusted}$	per 1000 people per year	Onset of diabetes accounting for baseline rate, obesity, physical activity, consumption of sugar-sweetened beverages, fruit and vegetable intake, and age
$DMOnsetRate_{Base}$	per 1000 people per year	Baseline DM onset rate (per 1000 people per year) set at 50
PreDMPop	People in 1000s	Pre-diabetes population (stock)

4.10 Comparison of SSCH diabetes incidence estimate to GBD incidence estimate

The only modeled country-level estimate for diabetes prevalence that exists for comparison is that from the Global Burden of Disease study. It uses a different methodology than what is applied here. We see from the figure below that overall the SSCH estimate tends to be higher than that from GBD for the same age groups, although the trends are similar. Neither of these estimates can be compared to actual figures as longitudinal studies of diabetes incidence in the region do not exist.

We are assuming that Jamaica experienced a rapid increase in incidence between 1990 and 2000 which is now flattening at a higher rate (around 11 cases per 1000 people per year). Trends from high income countries suggest it is similar in some developing countries(35) and some are even experiencing a decline.

Figure S4.2 Comparison of estimates for new cases of diabetes from SSCH model and the Global Burden of Disease for Jamaica

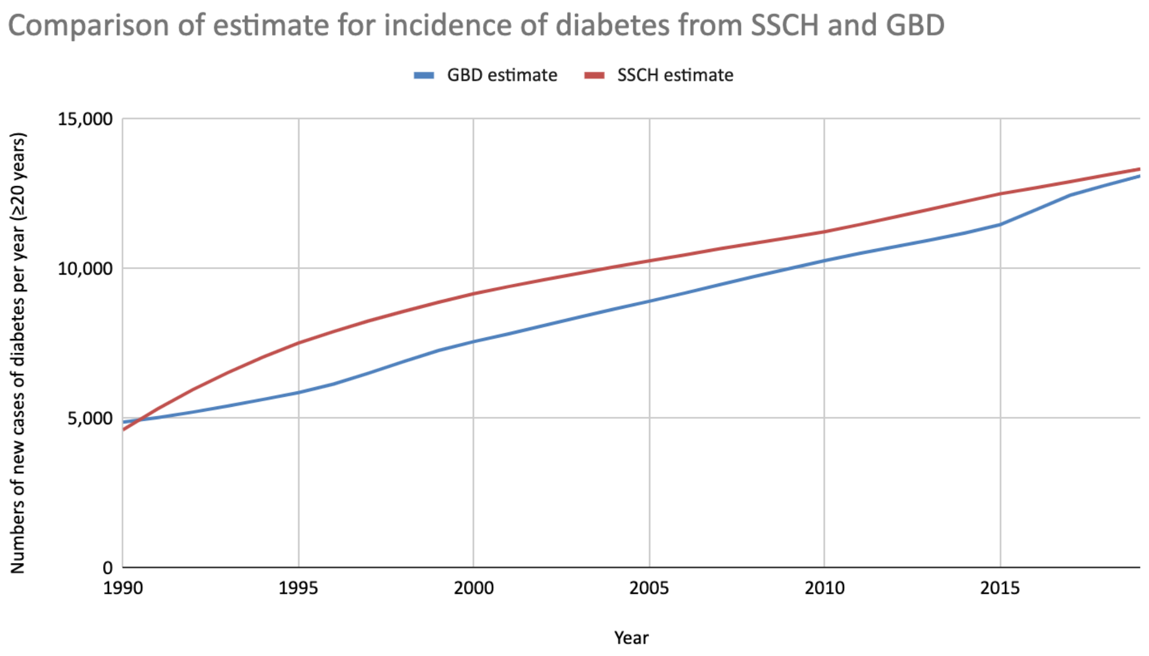

5 Pre-diabetes epidemiology

A similar structure underlies the assumptions regarding onset of prediabetes from NG, however, there is also a backward flow from prediabetes to NG that, as noted previously, is trivial and assumed not to exist at the population level in a significant way. There is some evidence for a increased risk of prediabetes in the obese compared to the non-obese, although the effect is not as large as that for diabetes. A baseline estimate for pre-DM onset in the non-obese is used, as with diabetes, and a relative risk of prediabetes in the obese is then used to estimate the effect of obesity on the overall incidence rate. There is not much research describing the influencing factors in prediabetes onset or indeed its epidemiology. Unlike with diabetes, there are fewer data for estimating prediabetes incidence, and particularly using the diagnostic criteria (ADA) that we apply here.

Table S5.1 Evidence in the literature for prediabetes incidence

**Country**	**Incidence estimate**	**Population Notes**
Chennai, India(36)	29.5 per 1000 person-years	≥20 years, all BMIs included but average BMI was 24.5 so lean (ADA 2010 criteria)
Kerala, India(37)	24.5 per 1000 person-years (IFG)	≥18 years, population-based cohort (ADA 2010 criteria)
Avhaz, Iran(38)	40.8 per 1000 person-years	≥20 years, Ahvaz, Iran using a "healthy population" (ADA 1997 criteria)
Denmark(39)	50 per 1000 person-years (any dysglycemia)	40+ (ADA 1997 criteria)
Tangshan, China(40)	26.3 per 1000 person-years (WHO criteria)	18+ Coal miners in Kailuan (ADA 2010 criteria)
Rotterdam, The Netherlands(41)	20.4 per 1000 person-years	45+ (WHO 1999 criteria)
Germany(42)	15.9 to 30.1% per year	55-74 KORA Study (WHO 1999 criteria)
Memphis, United States(43)	26-48% per year	18-65 Biracial white/Black cohort (ADA 2003 criteria)
Tehran, Iran(44)	36.8 per 1000 person years for women, 46.1 per 1000 per years for men	20+ years (ADA 2003 criteria)

There are a number of issues with the estimates from the studies presented above (which are not exhaustive) and especially when it comes to comparing the populations to those in the Caribbean. As an example, while the economic development and transition in India may be more similar to that of the Caribbean than those of Denmark or the Netherlands, that population presents with diabetes and prediabetes at a much leaner body type and seems to progress faster from normoglycemia. The age profiles of the populations are also quite different and no adjustment was possible. The cohort from China is among miners, and while large, may be subject to the “healthy worker” effect. Despite these caveats, we used these estimates as a guide for establishing a baseline rate for Jamaica. We estimate the baseline incidence of pre-DM in the non-obese at 22 per 1000 people per year. The evidence for this

is not strong for the population of interest, so we relied mostly on calibration which will be discussed below.

5.1 Effect of aging on prediabetes onset
This variable uses a weighted average of the fraction of the population over 55 across each of the stocks and depends on the relative risk of prediabetes in those over 55. The average relative risk of diabetes in those over 55 years is set at 1.2 following evidence used in the CDC diabetes model taken from the NHANES surveys of the US population(4).

5.2 Effect of obesity on prediabetes onset
This variable uses a weighted average of the fraction of obesity across each of the stocks and depends on the relative risk of prediabetes in the obese.

Table S5.2 Evidence of relative risk of prediabetes in the obese

Country	Ratio / RR	Notes
Avhaz, Iran(38)	1.28	calculated comparing overweight as there is no difference with normal weight
Tangshan, China(40)	1.47	averaging for men and women comparing the >20 BMI to the 20 - 22.9 range - Kailuan cohort
Rotterdam, The Netherlands(41)	1.68	averaging the estimate for lifetime risk of prediabetes in those obese compared to those under <25 BMI
The United States(45)	1.3	See supplementary tables Portal Study
Unadjusted average	1.43	

We estimate the relative risk for prediabetes onset from obesity to be 1.5. This was adjusted using the estimates above as well as through calibration.

5.3 Recovery from pre-DM
Prediabetes, even if left untreated, will revert back to normoglycemia for some people. Estimates for the proportion of people who annually revert back to normoglycemia are few and highly variable depending on the diagnostic criteria applied. Those with a more dysglycemic or impaired glucose tolerance will have a lower rate of reversion back to normoglycemia than those with a lower fasting blood glucose.

We take a broad definition of prediabetes that would include any form of dysglycemia. In similar cohorts, the rate of reversion from prediabetes to normoglycemia with no intervention is around 7% per year. This is the value we chose for the model and it calibrates well with the available estimates (Table 5.3). There is some evidence that weight loss among the obese may increase the rate of remission to normoglycemia in people with prediabetes(46). We included an effect for this possibility where if there is a net weight loss in the population, there would be an increase in the rate of recovery for the fraction of the prediabetes population that is obese.

Table S5.3 Summary of studies with information on reversion rates

Country	Rate	Annualized Rate	Notes
United Kingdom(47)	55-83% in 10 years	5-8%	Uses ADA criteria for the higher estimate population-based (40-69 years) - Ely Study
United States(48)	19% in 10 years	1.9%	Control arm of the study - used people with IGT or IFG and BMI 24 or higher - Diabetes Prevention Program
Tangshan, China(49)	44.92% in 10 years	4.5%	Prospective population-based cohort in Tangshan China study
United Kingdom(50)	45% in 5 years	9%	Observational cohort with a broad definition of pre-DM, Whitehall II Study
Spain(51)	31% in 3 years	10.3%	Cohort study in primary care setting with a broad preDM definition - Isolated FPG in this case PREDAPS
Germany(46)	9.2% to 27% in 6 years - depending on diagnostic criteria	1.5 - 4.5%	Older cohort 55 - 74 years (less likely to progress from preDM to DM) KORA Study
Barcelona, Spain(52)	41.6% at one year	41.6%	Prospective, population-based cohort in Catalunya 25+ years Mollerussa cohort

Figure S5.1 Structure for estimating pre-diabetes onset and recovery

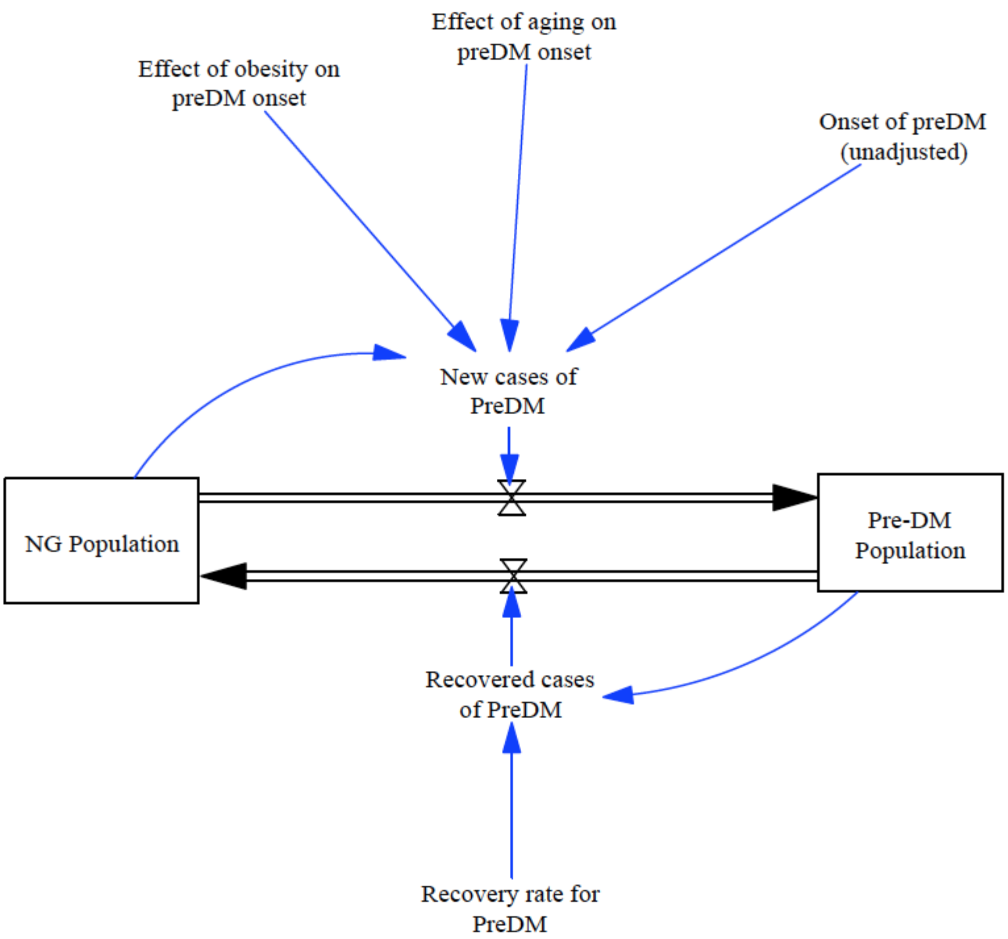

5.4 Equations to estimate the onset and recovery for prediabetes

$$EffectObesity_{PreDM} = ((1 - ObeseFracNG) + (ObeseFracNG) * RRPreDM_{Obese}) / ((1 - ObeseFracNG_{Init}) + (ObeseFracNG_{Init}) * RRPreDM_{Obese})$$

$$EffectAging_{PreDM} = ((1 - FracOver55/100) + (FracOver55/100) * RRPreDM_{Aging} / ((1 - FracOver55_{Init}/100) + (FracOver55_{Init}/100) * RRPreDM_{Aging}$$

$$PreDMOnset_{Rate} = BasePreDMOnset * EffectObesity_{PreDM} * EffectAging_{PreDM}$$

$$PreDMOnset_{Cases} = PreDMPop * PreDMOnset_{Rate}/1000$$

$$PreDMRecovery = NGPop * FracRecoveryPreDM + max(0, -BMIrate)* EffectWeightLossPreDM * ObeseFracPreDM$$

$$BMIrate =(BdWtChange_{Men} /Ht_{Men}\text{^}2 * FracMen) + (BdWtChange_{Women} / Ht_{Women}\text{^}2 *FracWomen)$$

Table S5.4 Variables to estimate the onset and recovery for prediabetes

Variable	Units	Description
BasePreDMOnset	Per 1000 people per year	Baseline prediabetes onset rate, set at 23
NGPop	People in 1000s	Normoglycemic population (stock)
ObeseFracNG	dimensionless	Obese fraction of the NG population
$ObeseFracNG_{Init}$	dimensionless	Initial Obese fraction of the NG population
$RRPreDM_{Obese}$	dimensionless	Relative risk of prediabetes in the obese, constant set at 1.5
$EffectObesity_{PreDM}$	dimensionless	Effect on the onset rate by obesity
FracOver55	dimensionless	Fraction of the population over 55 years
$FracOver55_{Init}$	dimensionless	Initial Fraction of the population over 55
$RRPreDM_{Aging}$	dimensionless	Relative risk of prediabetes in people over 55, constant set at 1.2
$EffectAging_{PreDM}$	dimensionless	Effect on the onset rate by aging
$PreDMOnset_{Rate}$	Per 1000 people per year	Adjusted prediabetes onset rate with effects for obesity and aging
$PreDMOnset_{Cases}$	People in 1000s	Total new cases of prediabetes
PreDMRecovery	People in 1000s	Cases recovering from prediabetes to normoglycemia
PreDMPop	People in 1000s	Pre-diabetes population (stock)
FracRecoveryPreDM	dimensionless	Fraction of people with prediabetes that recover per year; set at 7%
EffectWeightLossPreDM	dimensionless	Increase in recovery rate from weight loss in people with prediabetes who are obese
BMIrate	kg/m^2	Average change in BMI per year
$BdWtChange_{Men}$	kg	Change in average weight for men per year (see obesity calculation below)
$BdWtChange_{Women}$	kg	Change in average weight for women per year (see obesity calculation below)
FracMen	dimensionless	The fraction of men in the total population
FracWomen	dimensionless	The fraction of women in the total population

6 Estimating obesity

One of the key elements for linking population-based upstream interventions to effects on diabetes is through the estimation of the prevalence of obesity in the population, one of its most important risk factors. We estimate obesity as a percentage with a stock for average body weight for men and women. There are more sophisticated ways of estimating weight change using system dynamics models for obesity(53) which describe in detail the micro and macro dynamics of weight accumulation from energy imbalance. However, they depend on two aspects that are not available for this population: yearly data on a large population, and sufficient evidence to accurately determine the resting basal metabolic rate for African populations(54). In this model, the obesity calculation is a means of estimating the effect of upstream interventions on diabetes. This simplified version fits that purpose. If we were more interested in understanding the dynamics of energy expenditure in the population, then a more sophisticated model would be warranted. We will see in the calibration that this simplified model does a sufficient job of matching historical data on BMI from studies in Jamaica.

The stock and flow diagram presented here (Figure 6.2) is a summary but there are two separate diagrams: one for men and one for women, with the same structure. The figures estimated are an average for the whole of the population. The resting metabolic rate is determined from the adapted Harris-Benedict equations(55), which are well-established in the scientific literature for estimating energy expenditure. Average height is held constant. There is an inherent balancing loop within the estimation of body weight. Body composition and body weight adapts to the circumstances determined by physical activity level and caloric intake. Over time, a body will adapt at a new equilibrium weight if any change is made to the input variables (physical activity level and caloric intake). A body with a higher average weight will lead to a higher resting metabolic rate, a greater energy expenditure at the same physical activity level, and a lower average caloric balance, leading to a smaller change in body weight.

6.1 Estimating Obesity from BMI

We used data taken from the WHO NCD Risk Factor Surveillance (STEPs)(56) reports from African and diaspora populations that included objectively measured BMI and obesity to construct a for estimating obesity percentage prevalence from average BMI in a population. It was necessary to take estimates from more than just Caribbean populations in order to get data points at low ranges of BMI and obesity prevalence. For this purpose, estimates were taken from sub-Saharan African countries. Three binomial curves were fit to the data and those were used to construct the equations above.

Figures S6.1 a, b, c Binomial curves to estimate the obesity prevalence from BMI taken from African and diaspora population

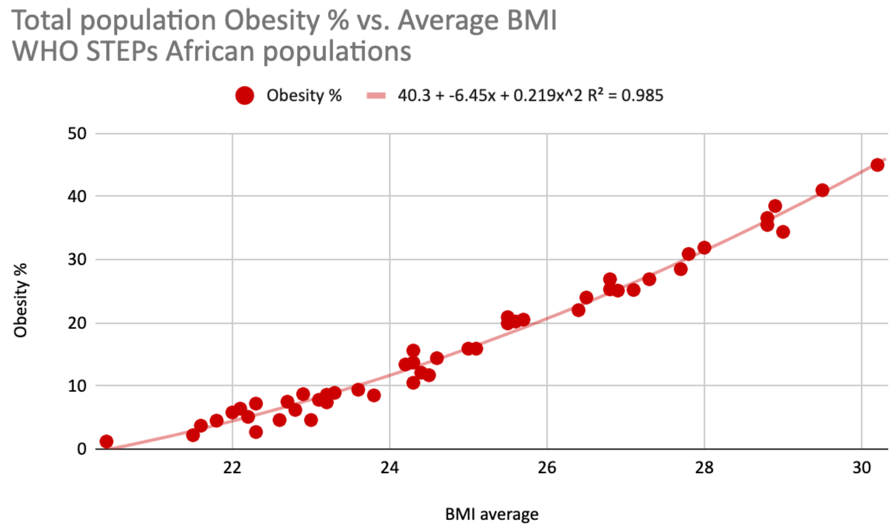

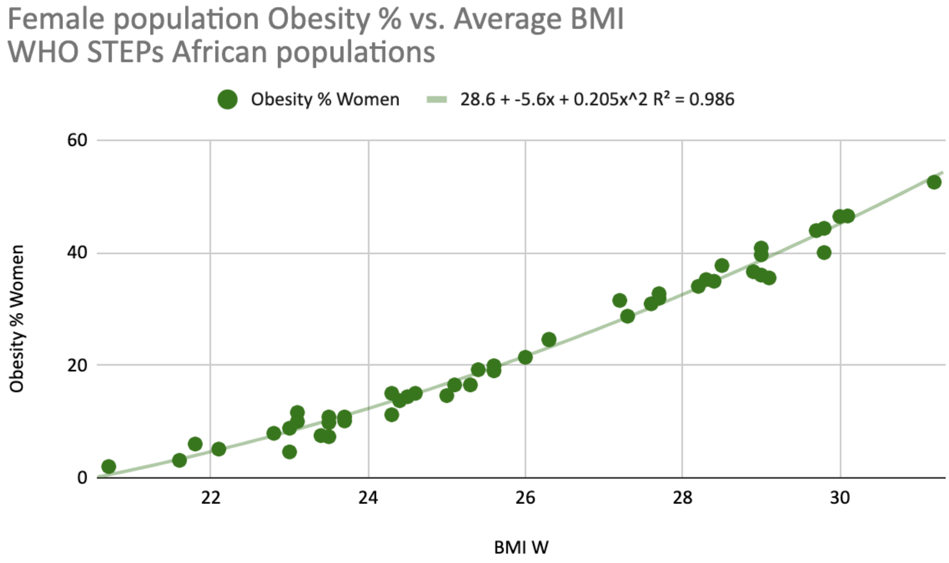

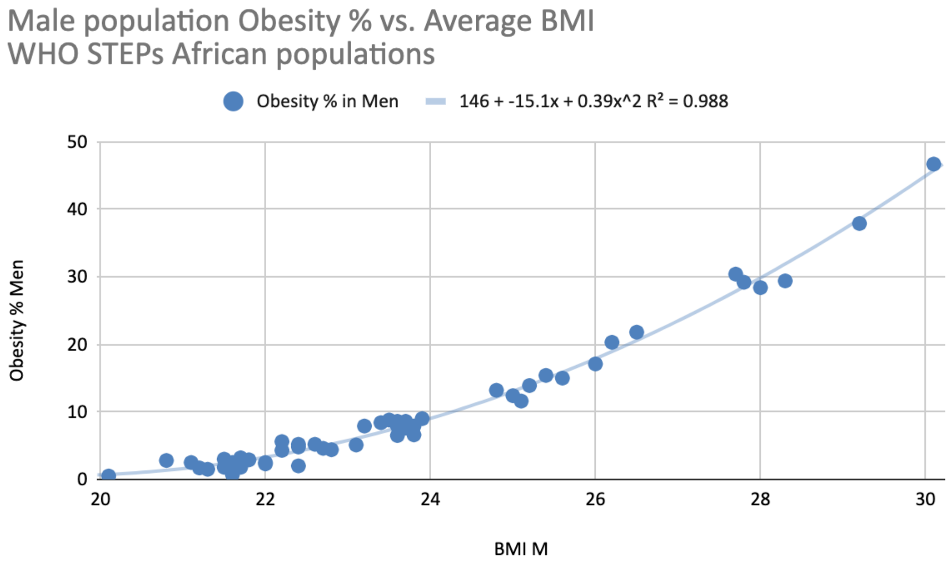

Figure S6.2 Structure for estimating BMI and obesity

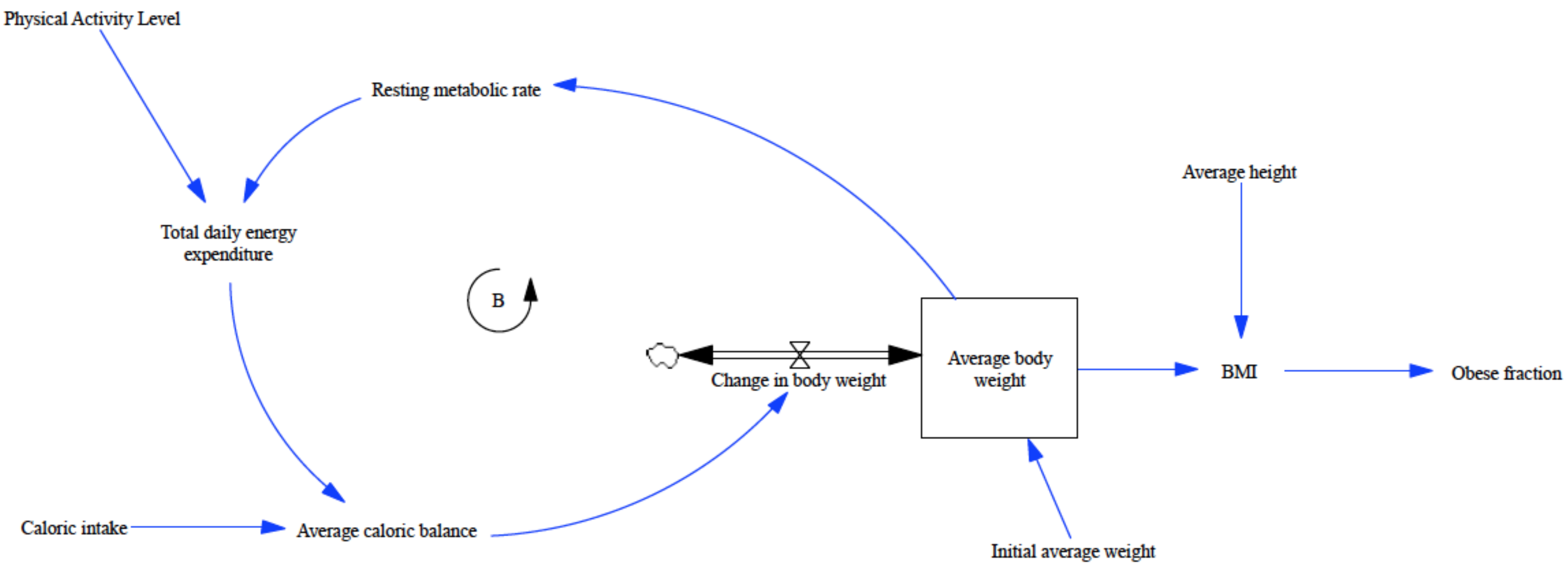

6.2 Equations used to estimate the obesity prevalence

Men

$BMI_{Men} = BdWt_{Men}/Ht_{Men}\text{^}2$

$ObeseFrac_{Men} = 145 - 15.1 * BMI_{Men} + 0.39 * BMI_{Men}\text{^}2$

$RMR_{Men} = 88.362 + 13.397 * BdWt_{Men} + 4.799 * Ht_{Men} - 5.677 * Age_{Men}$

$TDEE_{Men} = PAL_{Men} * RMR_{Men}$

$CalImb_{Men} = Intake_{Men} - TDEE_{Men}$

$BdWtChange_{Men} = CalImb_{Men} / ForbesC$

Women

$BMI_{Women} = BdWt_{Women}/Ht_{Women} \text{ ^ } 2$

$ObeseFrac_{Women} = 28.6 - 5.6 * BMI_{Women} + 0.205 * BMI_{Women}\text{^}2$

$RMR_{Women} = 447.593 + 9.247 * BdWt_{Women} + 3.098 * Ht_{Women} - 4.33 * Age_{Women}$

$TDEE_{Women} = PAL_{Women} * RMR_{Women}$

$CalImb_{Women} = Intake_{Women} - TDEE_{Women}$

$BdWtChange_{Women} = CalImb_{Women} / ForbesC$

Total Obesity

$BMI_{Total} = BMI_{Men} * FracMen + BMI_{Women} * FracWomen$

$ObesityPrev = 40.3 - 6.45 * BMI_{Total} + 0.219 * BMI_{Total} \text{ ^ } 2$

Table S6.1 Variables to estimate obesity and BMI

Variable	Units	Description
BMI_{Men} / BMI_{women}	kg/m^2	Body mass index in men / women
$BdWt_{Men}$ / $BdWt_{Women}$	kg	Average Body weight in men / women (stock)
Ht_{Men} / Ht_{Women}	m	Height in Men / Women
$ObeseFrac_{Men}$ / $ObeseFrac_{Women}$	dimensionless	Obese fraction caculated using BMI in the equation above in Men / Women
RMR_{Men} / RMR_{Women}	kcal/day/year	Average Resting metabolic rate using the modified Harris-Benedict equations(55) in a year for Men / Women
Age_{Men} / Age_{Women}	years	Average age of adult Men / Women taken from World Population Prospect estimates
PAL_{Men} / PAL_{Women}	dimensionless	Ratio for Physical Activity Level - the ratio of total energy expenditure divided by the basal metabolic rate in Men / Women
$TDEE_{Men}$ / $TDEE_{Women}$	kcal/day/year	Average Total Daily Energy Expenditure in a year for Men / Women
$CalImb_{Men}$ / $CalImb_{Women}$	kcal/day/year	Average daily Caloric Imbalance in a year for Men / Women
$Intake_{Men}$ / $Intake_{Women}$	kcal/day/year	Average daily caloric intake in a year for Men / Women
ForbesC	constant	Forbes Constant representing the number of calories needed to accumulate 1 kg of weight - set at 8050
$BdWtChange_{Men}$ / $BdWtChange_{Women}$	kg/year	Body weight change from caloric imbalance in a year (flow)
FracMen	dimensionless	The fraction of men in the total population
FracWomen	dimensionless	The fraction of women in the total population
BMI_{Total}	kg/m^2	Average total body mass index for the population
ObesityPrev	dimensionless	Fraction of the population with obesity

7 Physical Activity

Evidence from the region indicates a mostly physically inactive population. Data extracted from the WHO NCD Risk Factor Surveillance(56) surveys from the Caribbean show a self-reported median 90 minutes per day of moderate to vigorous physical activity (MVPA) across both genders with a substantial difference between men (media 171.04 minutes/day) and women (median 46.18 minutes/day).

Table S7.1 Evidence of Caribbean MVPA from STEPs Surveys(56)

	Total (MVPA min/day)	Men (MVPA min/day)	Women (MVPA min/day)	Ratio Men/Women
St Lucia	90	222.9	42.9	5.2
St Kitts	51.4	107.1	30	3.6
Dominica	162.9	295.7	68.6	4.3
Grenada	85.7	182.9	50.7	3.6
Cayman Islands	68.6	147.9	38.6	3.8
British Virgin Islands	128.5	240	60	4.0
Bermuda	48.6	77.1	34.3	2.2
Barbados	25.7	34.3	8.6	4.0
Trinidad and Tobago	42.9	100	20	5.0
Anguilla	192.7	269.9	118.6	2.3
Saint Vincent and the Grenadines	94.3	203.6	35.7	5.7
**Median**	**85.7**	**182.9**	**38.6**	**4.0**

The STEPs surveys use the International Physical Activity Questionnaire (IPAQ)(57) to establish physical activity patterns. The survey includes questions about domain-related physical activity, however, these are often not reported in the STEPs reports.

7.1 Jamaican data on physical activity

The Jamaica Health and Lifestyle Surveys also collected self-reported data on physical activity from respondents with different parameters than those in the IPAQ. We do not have the survey tools used but the reported data from the 2000 survey(58) suggest that close to 67% of adults reported being sedentary with an additional 20% reporting only light activity. The 2007/08 JHLS(20) also did not report physical activity measures using a standardized tool. However, the survey reports that approximately 90% of Jamaicans were either sedentary or participated in light physical activity during leisure time. Overall around 60% of women reported low or no physical activity, compared to 25% of men. We do not currently have data from the JHLS III survey conducted in 2017(17).

To our knowledge, only one study(59) has published data from 2010 on objectively measured physical activity in a Jamaican cohort. The sample size is small (around 450

adults) and were taken from Spanishtown. Evidence from that study suggests that adults spend more than 200 minutes per day in sedentary time and just 9 minutes per day in MVPA in 10-min bouts or 22 in 1-min bouts. The study also found significant differences between men and women (MVPA - Men around 30 min/d vs 18 min/d in Women); sedentary time was about the same.

Figure S7.1 Self-reported physical activity levels from the Jamaica Health and Lifestyle Survey 2007/2008(20)

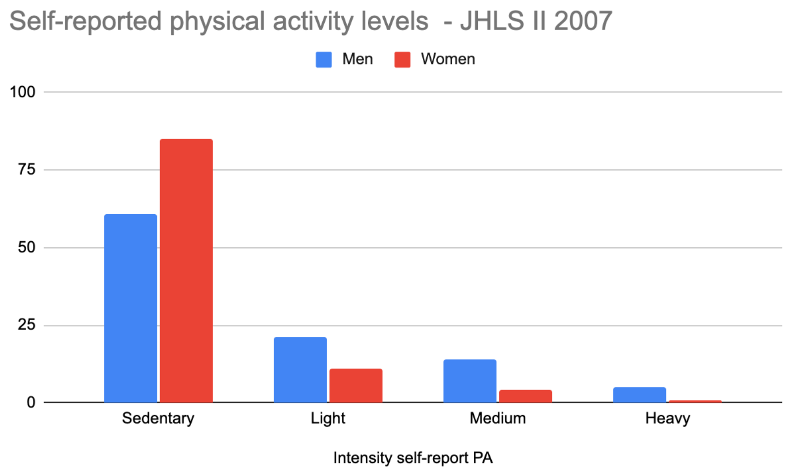

The study also collected self-reported information using the Global Physical Activity Questionnaire (GPAQ)(60) which captures physical activity across different domains (travel, work, recreation, sedentary time and total minutes of MVPA). The results of these show that the majority of physical activity being done by men is in occupational time with much less in recreational time. This difference is less pronounced in women who overall have a much lower occupational PA level and a comparable recreational PA level.

Figure S7.2 Self-report PA (min/d, GPAQ) for occupation, travel and recreation PA, by site for men and women from the METS study(59) JA = Jamaica

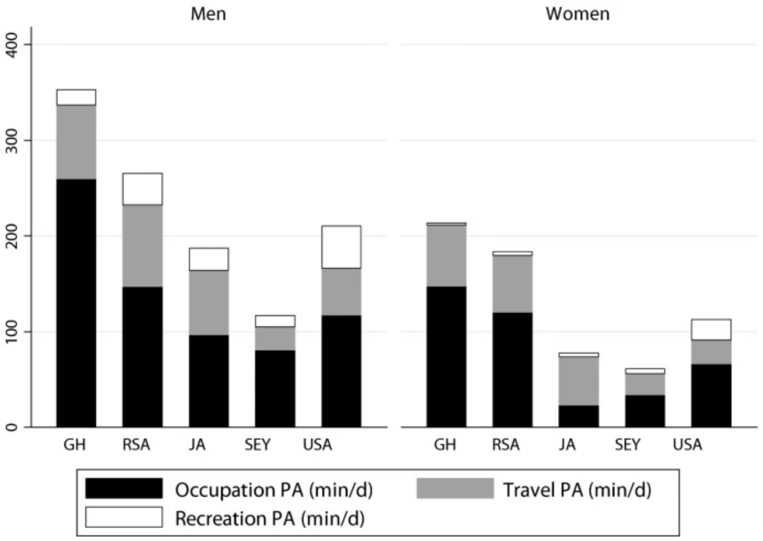

7.2 Modeling Physical Activity data for Jamaica

While it is clear that the population in Jamaica is largely sedentary, and that this state has persisted for some time, it is difficult to draw directly from the data what the physical activity parameters should be. We separated total physical activity into component domains and used modeled data from other studies, together with some assumptions from the existing data to construct a time trend for physical activity that roughly corresponds to the evidence available.

7.3 Data inputs for physical activity time series

As discussed above, we do not have sufficient information directly from the region to establish time trends so modeled data are used. We took the modeling approach described in Ng and Popkin(61) to estimate shifts in physical activity over time based on their assessments of changes in Brazil. They modeled these data from ILO statistics and the Compendium on Physical Activity.

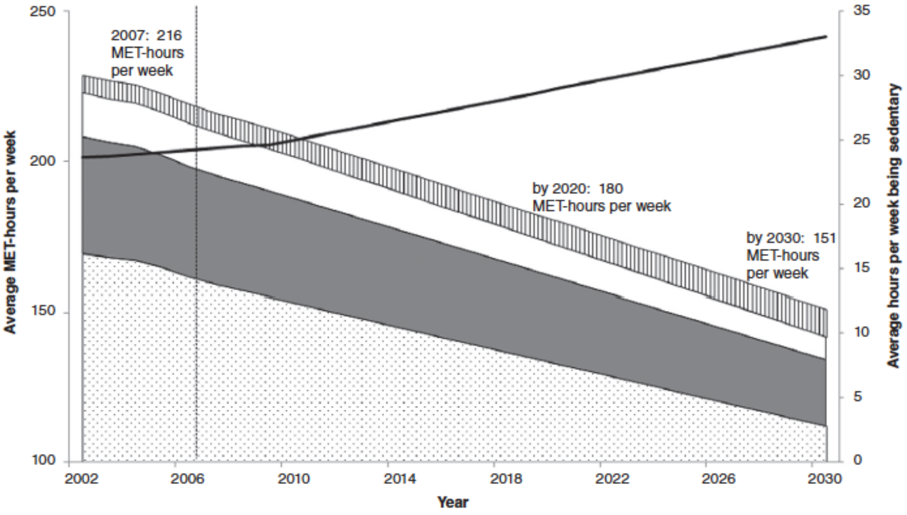

Figure 3 Brazilian adults metabolic equivalents of task (MET)-hours per week of all physical activity, and hours per week of time in sedentary behaviour: measured for 2002–2007, forecasted for 2009–2030.
Source: Occupational physical activity based on 2002–2007 UN-ILO statistics applying MET-intensity values from the Compendium of Physical Activity. Active leisure measure is from Sao Paolo, Brazil Physical Activity Study; applying the following MET-intensity value following the IPAQ guidelines: walking = 3.3 MET per hour; moderate only = 4 METs per hour; vigorous only = 8 METs per hour. All other energy expenditure and sedentary time values are estimated from applying the average activity measures found in the United States and China from periods of similar economic development (based on per capita gross domestic product purchasing power parity) for each of these countries (2002 Brazil to 1975 United States and 2006 China; 2008 Brazil to 1985 United States and 2009 China). Forecasting for 2009–2030 based on 2002–2007 slopes.
▥ active leisure PA, ☐ travel PA, ■ domestic PA, ⛆ occupational PA, ▬ sedentary time.

We assume a similar trend for a decrease in physical activity for Jamaica, although the starting points and relative contributions are slightly different. The paper assumes a roughly 1.5% annual decrease across occupational, domestic, and travel physical activity, and an annual increase of 2% in leisure physical activity. The total share of each domain is also important, occupational physical activity accounts for roughly 50% of all physical activity, followed by 27% domestic physical activity, 17% in travel, and just 5.5% in leisure.

We took the data presented in the JHLS 2000 survey to establish a baseline for what could be the initial MVPA. To do this, we took an average of the self-reported minutes spent in travel PA by age group and used a midpoint for the categories presented. We then estimated an average (mean) number of minutes for each category. Using this method we estimate around 16 minutes on average for travel MVPA per day in 2000, and 5 minutes for leisure time MVPA. These estimates are similar to those estimated for adults in high income countries(62) although higher than the proportion of MVPA from travel reported in Barbados(63) (17 vs 4% respectively). We then apply the proportions described above, which are similar to the GPAQ reported results from Jamaica in the METS study(59).

Figure S7.3 Modeled trends for the SSCH inputs for physical activity

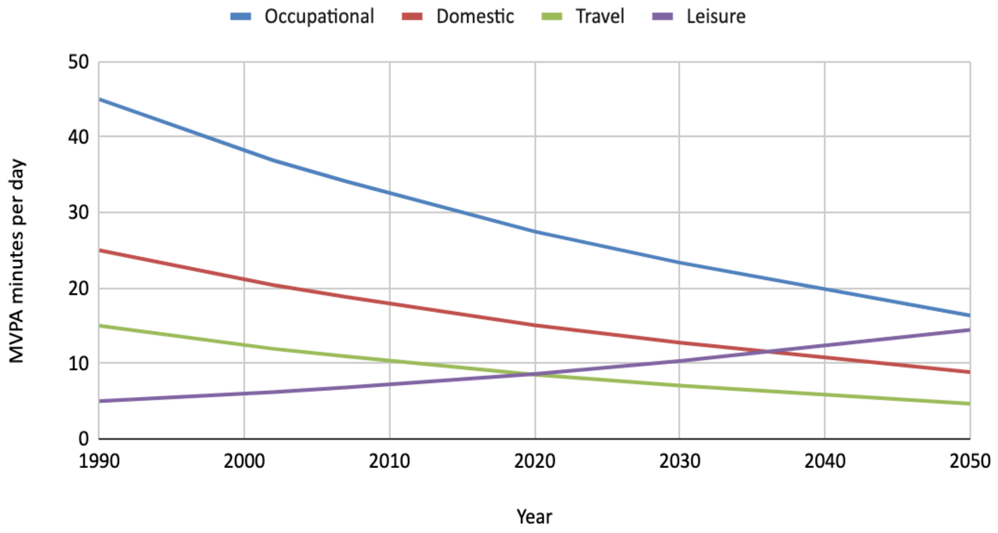

Table S7.2 Time series inputs for MVPA by domain

MVPA minutes per day	1990	2002	2007	2020	2030	2050
Occupational	45	36.90	34.13	27.48	23.36	16.35
Domestic	25	20.39	18.83	15.07	12.75	8.84
Travel	15	11.94	10.93	8.51	7.06	4.66
Leisure	5	6.20	6.82	8.59	10.31	14.44
Total	90	75.43	70.70	59.65	53.49	44.28

Another important study that has been used to calibrate these estimates was conducted in 2015 by Howitt et al.(63) in Barbados among adults 25 - 54 years. The study combines objectively measured data with the Recent Physical Activity Questionnaire (RPAQ) in the same sample. The study showed an overall average MVPA of 62.5 minutes per day (56.6 min/day in Women; 91.6 min/day in Men). Crucially, the study showed an average 30% overestimation of physical activity by subjectively measured compared to objectively measured PA. Over 60% of respondents overestimated their activity and the magnitude of overestimation was consistent across a number of sociodemographic strata.

The proportions of PA reported by participants in the survey were similar to those estimated in the data above for occupation and domestic PA (67% and 18%) but higher for leisure-time PA and lower for travel PA (21% and 4%) respectively. Obesity prevalence in adults in Barbados(18) is higher than that estimated for Jamaica(17) (33.8% versus 28.6%) and may be a result of differences in risk factors. The population from Barbados appears to be more sedentary, although, in the absence of objectively measured data from a recent sample in Jamaica, this is hard to ascertain.

The estimates for 2020 correspond roughly with the data from Barbados under the assumption that Jamaicans are on average more physically active, but that the differences between the sexes are similar to those across the Caribbean and that those differences persist into the future in the model projections.

Figure S7.4 Structure for estimating MVPA

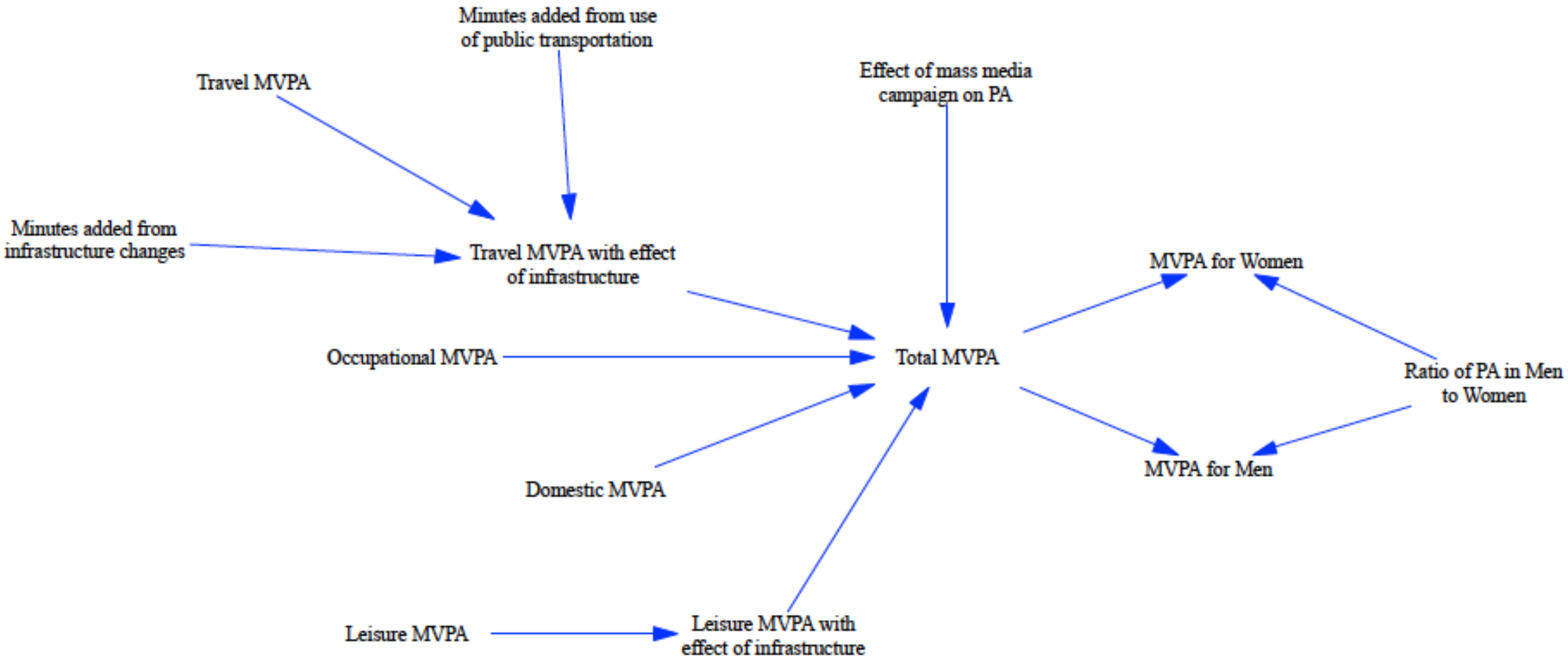

7.4 Equations for estimating MVPA

$MVPA_{Travel + infrastructure} = MVPA_{Travel} + InfrastructureMinutes_{Travel} + PublicTransportMinutes$

$MVPA_{Leisure + Infrastructure} = MVPA_{Leisure} + InfrastructureMinutes_{Leisure}$

$MVPA_{Total} = MVPA_{Travel + infrastructure} + MVPA_{Occupational} + MVPA_{Domestic} + MVPA_{Leisure + Infrastructure}$

$MVPA_{Total+Campaign} = MVPA_{Total} * PACampaign$

$MVPA_{Women} = MVPA_{Total} / (FracWomen + FracMen * RatioPA)$

$MVPA_{Men} = MVPA_{Women} * RatioPA$

Note: where there are variables related to interventions (infrastructure changes, increases in public transportation use, mass media campaigns etc.) the code of the model only activates this if a “switch” is turned on. For the baseline predictions, these are assumed to have no effect on the underlying assumptions for physical activity.

Table S7.3 Variables for estimating MVPA

Variable	Units	Description
$MVPA_{Travel}$	minutes/day	Average daily moderate-to-vigorous physical activity from travel
$MVPA_{Travel + infrastructure}$	minutes/day	Average daily moderate-to-vigorous physical activity from travel including the possible effects of improving travel infrastructure
$InfrastructureMinutes_{Travel}$	minutes/day	Average daily MVPA Minutes added from infrastructure changes to travel - set at 4 minutes per day(64)
PublicTransportMinutes	minutes/day	Average daily MVPA Minutes added from use of public transportation - set at 4.3 minutes per day(65)
$InfrastructureMinutes_{Leisure}$	minutes/day	Average daily MVPA Minutes added from infrastructure changes to leisure - set at 4 minutes per day(64)
$MVPA_{Leisure}$	minutes/day	Average daily moderate-to-vigorous physical activity from leisure time
$MVPA_{Occupational}$	minutes/day	Average daily moderate-to-vigorous physical activity from occupational activity (time series described above)
$MVPA_{Domestic}$	minutes/day	Average daily moderate-to-vigorous physical activity from domestic activity (time series described above)
$MVPA_{Total}$	minutes/day	Average daily moderate-to-vigorous physical activity
PACampaign	dimensionless	Increase in MVPA from a mass-media campaign set at a pooled relative risk of 1.53(66)
$MVPA_{Women}$	minutes/day	Average daily moderate-to-vigorous physical activity in Women
FracWomen	dimensionless	Fraction of women in the population taken from the World Population Prospects
FracMen	dimensionless	Fraction of men in the population taken from the World Population Prospects
RatioPA	dimensionless	The ratio of physical activity in Men compared to Women. Constant set at 3.5
$MVPA_{Men}$	minutes/day	Average daily moderate-to-vigorous physical activity in Men

7.5 Physical Activity Level estimation

MVPA is just one component of estimating physical activity. Total physical activity also includes sedentary time, time spent in light physical activity, and time spent sleeping. These are used to estimate physical activity level (PAL), a metric developed by the FAO/WHO/UNO to estimate the ratio of total daily energy expenditure to basal metabolic rate.

We estimate a Physical Activity Level (PAL), a ratio of total energy expenditure divided by the basal metabolic rate, using estimates of physical activity time and intensity. The PAL was developed by the Food and Agriculture Organization, World Health Organization, and United Nations University in 1985(67) to understand energy requirements for different populations.

Table S7.4 Reference ranges for PAL

Category	PAL value
Sedentary or light activity lifestyle	1.40-1.69
Active or moderately active lifestyle	1.70-1.99
Vigorous or vigorously active lifestyle	2.00-2.40*

7.6 Estimating physical activity intensity

The energy intensity of different types of activities are taken from the Compendium of Physical Activities estimates(68) for METs or metabolic equivalents, which are the ratio of metabolic rate related to an activity over the resting metabolic rate. One MET is defined as 1kcal/kg/hour and is roughly equivalent to the energy cost of sitting. Below are some examples of MET values associated with typical activities.

Table S7.5 MET values for typical activities

Activity	Estimated METs
Cycling	7.5
Walking	3.0
Riding in a bus	1.3
Household cleaning	3.3
Playing football	7.0
Sitting at a desk	1.5
Farming	4.8
Sleeping	0.95
Watching TV	1.0

There is a lot of variation in the estimated METs depending on the intensity of an activity. For instance, cycling competitively is associated with almost double the METs of cycling at a

leisurely pace. We do not have the kind of detail we may want for the intensity of the types of activities the population is engaged in, but at the same time, what is most important for this model is to arrive at a population average. Thus, we use some general average MET values that combine different types of activities. These averages are presented in the table below and are the ones used in the calculations.

Table S7.6 MET assumption inputs for PA categories

Type of PA	Model Average estimated METs
Sleeping	0.95
Light physical activity	2
Sedentary physical activity	1.5
Moderate to vigorous physical activity	5.2

These estimates can be changed, particularly in sensitivity analyses, to understand their relative impact on the PAL estimates. PAL can also be estimated as the sum of MET-minutes over the total minutes in a day(69).

7.7 Differences in physical activity levels between men and women

Because we assume that men are engaging on average 3 times more in MVPA than women, the sedentary time for women is also assumed to be greater than that for men and changes over time as MVPA decreases. Light PA, associated with walking around the house, doing small chores etc. is not expected to change much over time, but there is a modeled increase in sedentary time which matches the estimates from Ng and Popkin above.

Figure S7.5 SSCH modeled PAL levels for men and women

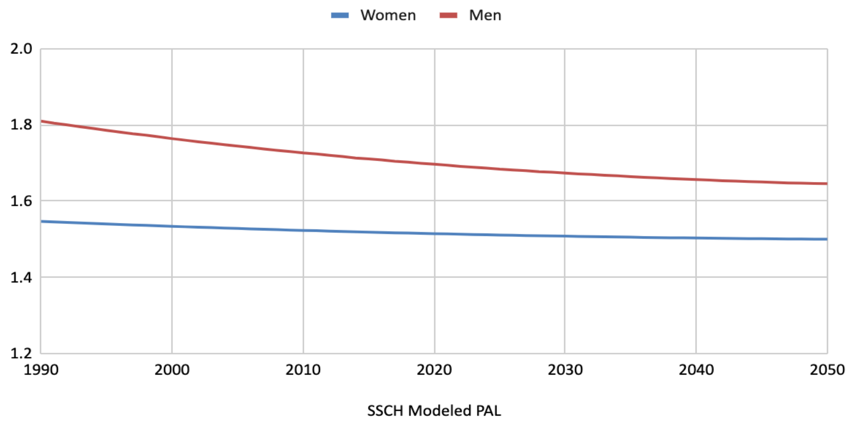

Men modeled as being 3.5 times more physically active at the outset with the gap narrowing over time. This narrowing is a result of an overall decrease in physical activity across both genders but more markedly in men. Women's PAL starts in the sedentary range (1.547) and decreases only slightly to end at 1.5 by 2050. Men's PAL is projected to decrease more dramatically from 1.8 in 1990 (which corresponds to a moderately active level) to a sedentary level of 1.65 by 2030.

Figure S7.6 Structure for calculating Physical Activity Level

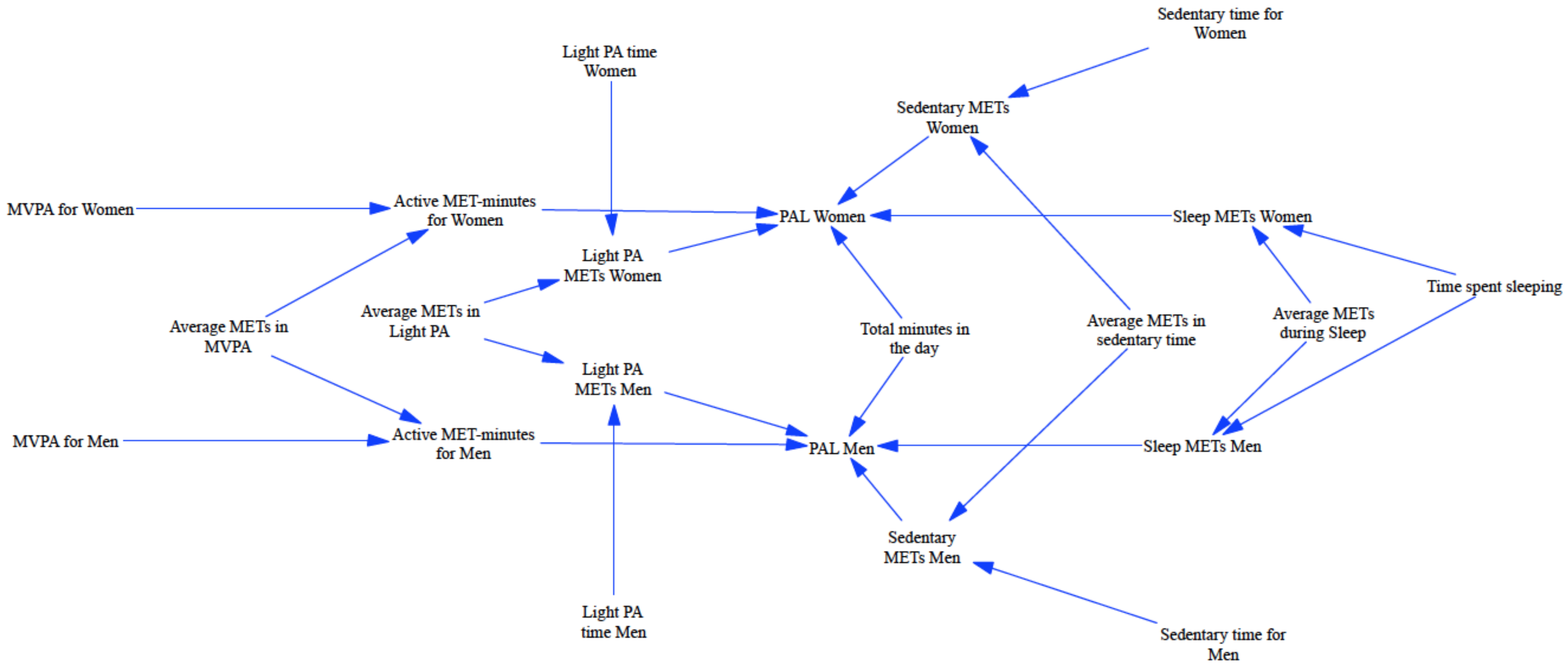

7.8 Equations to estimate physical activity levels

Men

$MVPAMETMinutes_{Men} = MVPA_{Men} * AverageMETs_{MVPA}$

$LPAMETMinutes_{Men} = LPA_{Women} * AverageMETs_{LPA}$

$SedentaryMinutes_{Men} = TotalMinutes - MVPA_{Men} - SleepMinutes - LPA_{Men}$

$SedentaryMETMinutes_{Men} = SedentaryMinutes_{Men} * AverageMETs_{Sedentary}$

$SleepMETMinutes = SleepMinutes * AverageMETs_{Sleep}$

$PAL_{Men} = (MVPAMETMinutes_{Men} + LPAMETMinutes_{Men} + SedentaryMETMinutes_{Men} + SleepMETMinutes)/TotalMinutes$

Women

$MVPAMETMinutes_{Women} = MVPA_{Women} * AverageMETs_{MVPA}$

$LPAMETMinutes_{Women} = LPA_{Women} * AverageMETs_{LPA}$

$SedentaryMinutes_{Women} = TotalMinutes - MVPA_{Women} - SleepMinutes - LPA_{Women}$

$SedentaryMETMinutes_{Women} = SedentaryMinutes_{Women} * AverageMETs_{Sedentary}$

$SleepMETMinutes = SleepMinutes * AverageMETs_{Sleep}$

$PAL_{Women} = (MVPAMETMinutes_{Women} + LPAMETMinutes_{Women} + SedentaryMETMinutes_{Women} + SleepMETMinutes)/TotalMinutes$

Data inputs

- Light PA time is estimated at around 360 minutes (6 hours) as per Howitt et a(63)l
- The time spent in light PA and sleeping are assumed to be the same for both men and women.
- Sedentary time is estimated as the time left after MVPA, light PA, and sleeping time have been subtracted from the total minutes in a day.

Table S7.7 Variables to calculate physical activity level

Variable	Units	Description
$MVPAMETMinutes_{Men}$ / $MVPAMETMinutes_{Women}$	MET-Minutes	MET-Minutes for Men / Women for moderate-to-vigorous physical activity
$MVPA_{Men}$ / $MVPA_{Women}$	minutes/day	Average daily moderate-to-vigorous physical activity in Men / Women
$AverageMETs_{MVPA}$	dimensionless	Metabolic equivalents - ratio of your working metabolic rate relative to your resting metabolic rate. Set as a constant 5.2 for MVPA
$LPAMETMinutes_{Men}$ / $LPAMETMinutes_{Women}$	MET-Minutes	MET-Minutes for Men / Women for light physical activity
LPA_{Men} / LPA_{Women}	minutes/day	Average daily light physical activity in Men / Women
$AverageMETs_{LPA}$	dimensionless	Metabolic equivalents - ratio of your working metabolic rate relative to your resting metabolic rate. Set as a constant 2 for LPA
$SedentaryMinutes_{Men}$ / $SedentaryMinutes_{Women}$	minutes/day	Time spent sedentary for Men / Women
TotalMinutes	minutes	Total minutes in a day = 1440
SleepMinutes	minutes	Minutes spent sleeping; estimated at 8 hours or 480 minutes
$SedentaryMETMinutes_{Men}$ / $SedentaryMETMinutes_{Women}$	MET-minutes	MET-Minutes for Men / Women in sedentary time
$AverageMETs_{Sedentary}$	dimensionless	Metabolic equivalents - ratio of your working metabolic rate relative to your resting metabolic rate. Set as a constant 1.5 for Sedentary time
SleepMETMinutes	MET-minutes	MET-Minutes in sleep (assumed to be the same for Men and Women)
$AverageMETs_{Sleep}$	dimensionless	Metabolic equivalents - ratio of your working metabolic rate relative to your resting metabolic rate. Set as a constant 0.95 for sleep
PAL_{Men} / PAL_{Women}	dimensionless	Ratio for Physical Activity Level - the ratio of total energy expenditure divided by the basal metabolic rate in Men / Women

8 Caloric intake

The other side of the obesity calculation is the estimation of caloric intake. Total caloric intake is estimated as the sum of calories from ultra-processed foods, fruit and vegetables, sugar-sweetened beverages and other sources. There are many studies associating different food group intake with diabetes risk and with different effects on obesity. However, the most well-documented independent connection to diabetes incidence from diet is sugar-sweetened beverage intake(26). Intake is high in the Caribbean as a whole and is estimated by the Global Dietary Database(70) at more than 2 servings per day per person for Jamaica. Ultra-processed foods may also have an effect on the incidence of diabetes(71) independent of obesity, although the relationship is less clear.

Studies that have looked at the effects of ultra-processed food consumption on diabetes incidence did not separate sugar-sweetened beverage consumption so it is difficult to ascertain whether any of the associated increase in risk comes just from the foods. However, ultra-processed foods are also of public health interest and have been gaining a focus as a possible intervention point through taxation and other regulatory measures. We therefore thought it important to include as a separate source of caloric intake in the model, even if it is not connected independently to diabetes onset. Finally, fruit and vegetable consumption are also associated with diabetes onset and studies have shown a dose-response relationship (discussed above) between fruit and vegetable consumption and a reduction in the relative risk of diabetes. Fruit and vegetable consumption across the Caribbean(72) is very low and increasing this consumption is seen as an important public health priority, not only as a possible way to reduce obesity but for the many benefits associated with a healthier diet. For the sake of simplicity, we did not attempt to disaggregate other caloric intake but rather kept it as a group. This could, in the future, be separated out by different food groups although it would be necessary to have sufficient evidence to support the initial data points as well as trends and projections.

8.1 Estimating caloric intake for men and women

Because the calculation for obesity relies on data disaggregated by sex, we made an estimation of intake for men and women. To do this we apply a similar approach to above. We used a ratio of the average total intake in men and women and the proportion of men and women in the population to estimate the average disaggregated intake from the total intake. The ratio to do this was estimated from data from a number of studies conducted in Jamaica, Barbados, and Jamaican diaspora populations (Table 8.1). The studies use either 24h recall data or food frequency questionnaires, as well as some objectively measured total energy intake using the doubly-labeled water method. The ratio that best calibrates with the historical data and also fits within the ranges reported in the evidence is 1.17.

Table S8.1 Caloric intake studies relevant to Jamaica and the Caribbean

Study	Country	Year	Measure	kcal/day men	kcal/day women	ratio
METs-study (Spanishtown)(73)	Jamaica	2010	24hr recall	2030	1671	1.2
METs-study (Spanishtown)	Jamaica	2010	DLW	2531	2077	1.22
Spanishtown cohort study(74)	Jamaica	2003	Food Frequency Questionnaire	2906	2327	1.25
BFACS(75)	Barbados	2000	24h recall	2136	1715	1.2
BNCS(75)	Barbados	2004	Food diaries	2127	1832	1.15
Health of the Nation(76)	Barbados	2014	24h recall	2333	1840	1.3

8.2 Sugar-sweetened beverage intake

The Caribbean drinks more sugary beverages(70) than anywhere else in the world. The trends do not seem to have changed much over the last thirty years, although they are projected to increase slightly. The JHLS surveys do not report continuous variables for SSB consumption, but the Global Dietary Database provides estimates for grams per day consumption for Jamaica from 1990 to 2015; these are estimated at 250g per serving. We assume projections going forward from 2015 to 2050 remain static at 2.2 servings/day.

Table S8.2 Sugar sweetened beverage intake time series inputs

**Year**	**SSBs (g/day)**	**SSBs (servings/day)**
1990	511.6145	2
1995	528.83643	2.1
2000	521.73163	2.1
2005	528.04199	2.1
2010	528.59027	2.1
2015	545.49304	2.2
2015 - 2050 SSCH assumption	545	2.2

8.3 Ultra-processed food consumption

We made a separation between ultra-processed food consumption from SSBs for the reasons stated above, but also to understand the independent effect of regulating just food products or just SSBs. Estimates for ultra-processed food consumption rely on data taken from a seven country study led by the Pan-American Health Organization on the consumption of ultra-processed food and drinks in Latin America(77). The report used the NOVA classification(78) for food and drinks and kcal per capita were estimated from sales trends, the countries included Argentina, Brazil, Chile, Colombia, Mexico, Peru and Venezuela. The sales trends from the study are projected below.

Figures S8.1, a,b Average daily retail of ultra-processed products in Latin America(77)

Figure 1

Average daily retail sales per capita of ultra-processed products in seven Latin American countries, 2009-2014, and projections for 2015 to 2019 (measured in kcal)

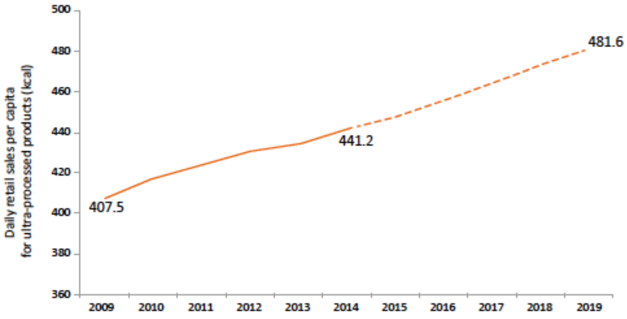

Data are presented as kilocalories per capita.

ii Infant formula sales rose from an average of 50.1 kcal per capita/day in 2009 to 68.4 kcal per capita/day in 2014 and are projected to rise further to 86.7 kcal per capita/day in 2019 *(45)*.

Figure 3

Average daily retail per capita sales of ultra-processed food and drink products in seven Latin American countries, 2009-2014 and projections for 2015 to 2019 (measured in kcal)

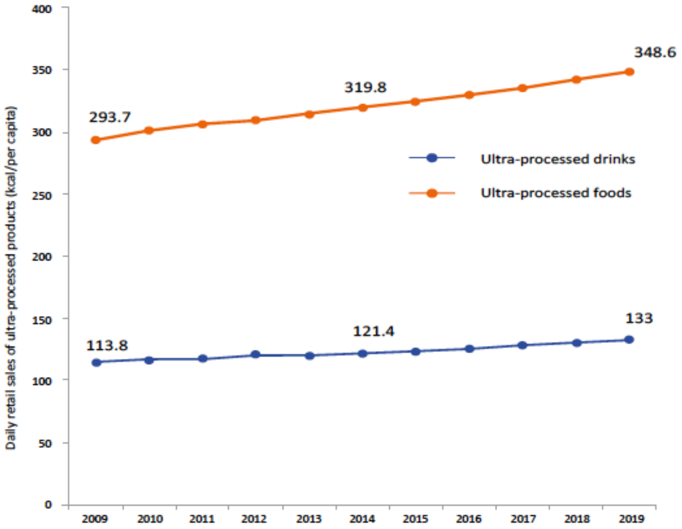

Data are presented as kilocalories per capita.

We know that sugar-sweetened beverage consumption is higher in the Caribbean than in Latin America, so it was important to separate out the calories coming from ultra-processed foods from those in ultra-processed drinks. The study reports an estimate of those calories separated by food and drink. The trends were extended for the estimates of kcal from foods to begin in 1990 at around 200kcal and continue to 640kcal in 2050.

Figure S8.2 SSCH modeled ultra-processed foods calories

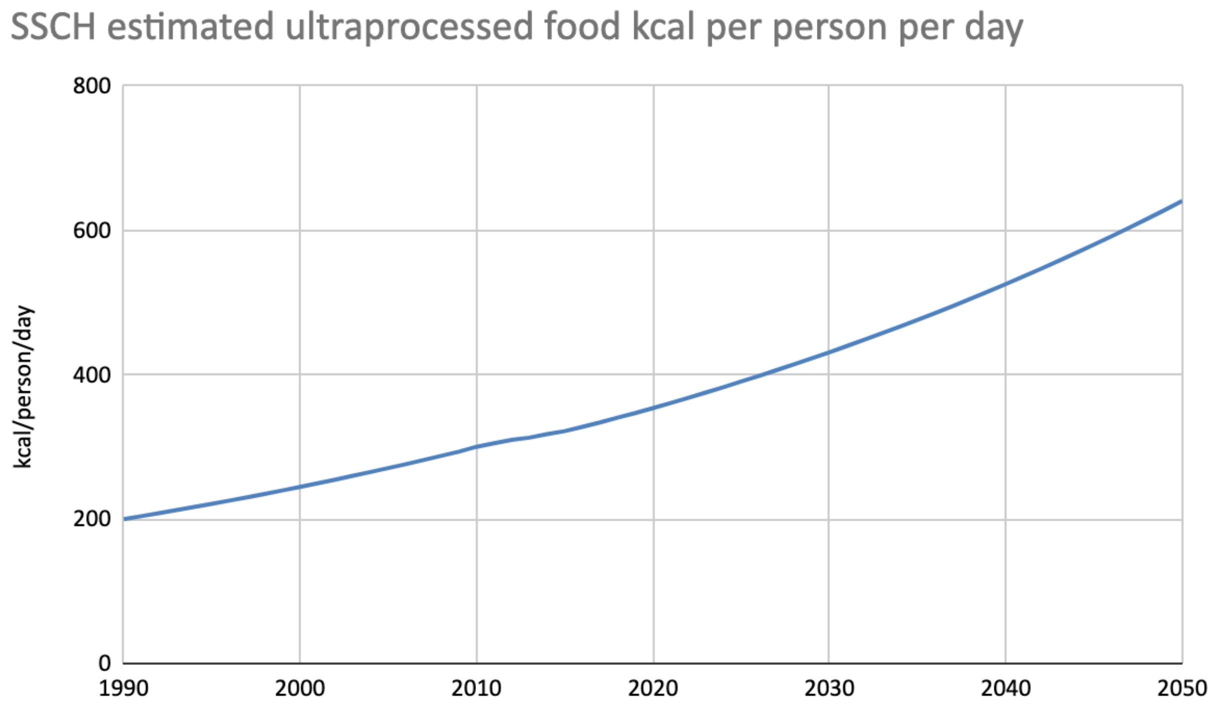

We only have a few studies from which to track the caloric intake of ultra-processed foods for the Caribbean. A recent study of adults in Barbados (25 - 64 years in 2012) found that 40.5% of total caloric intake was from ultra-processed food including 8.4% from sugar-sweetened beverages. Thus, roughly 30% of total intake in Barbados is from ultra-processed foods. This proportion can be compared to estimates from other parts of the world in the table below.

Table S8.3 Studies reporting estimates of ultra-processed food consumption

Country	Method/population	Proportion UPF including SSBs (%)	Total intake (kcal/day)
United Kingdom(79)	UK National Diet and Nutrition Survey 19+ years; food diary	54.3%	1823
Brazil(80)	Household Budget Surveys (1987, 1995, 2002, 2008); urban households, last was national sample; food purchase records	1987 - 18.7%<br>1995 - 21.0%<br>2002 - 26.1%<br>2008 - 29.6%	NR
Mexico(81)	Mexican National Health and Nutrition Survey 2012 (all ages); 24h recall	26.2% (adults)	1923
United States(82)	NHANES 2009- 2010 (all ages) 24h recall	57.6%	8.6 MJ/d
Chile(83)	Population-based national survey (all ages), 2010, 24h recall	28.6%	1819
Barbados(76)	Health of the Nation Survey, population-based adults 25-64 years, 24h recall	40.5%	2071

Using the average trends for ultra-processed food intake from the seven country PAHO study, we calibrate to levels similar to those in Barbados. The resulting SSCH estimates for the proportion of consumption of ultra-processed foods (including SSBs) from total kcal is presented below.

Figure S8.3 SSCH estimates of proportion of total caloric intake from ultra-processed foods

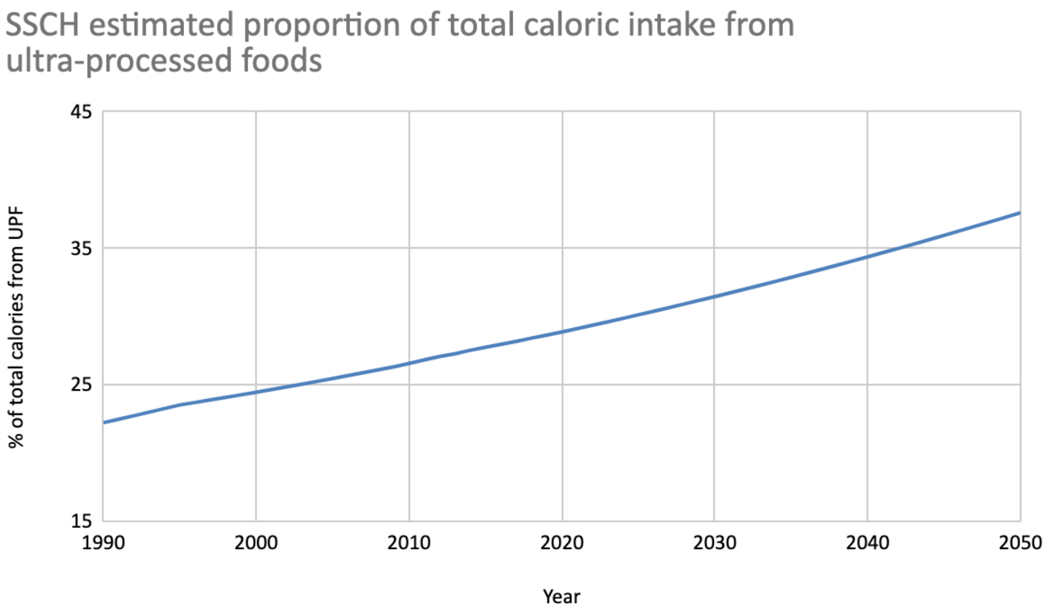

The estimate for kcal from other sources is held steady at 1550 kcal/day. Fruit and vegetable intake is estimated at 200 kcal/day (or 200g per day at 1 kcal per g).

8.4 Estimates of total caloric intake

Adding these together, we estimate the following intake for men and women. It should be noted that data from the METs study(73) indicate that participants in Jamaica were underreporting caloric intake by about 30%. We take this into account in the calibration of the estimates of intake presented here. The evidence taken from surveys done in Barbados and Jamaica are presented as dots along the curves below to show the calibration.

Figure S8.4 SSCH total caloric intake estimates for men and women

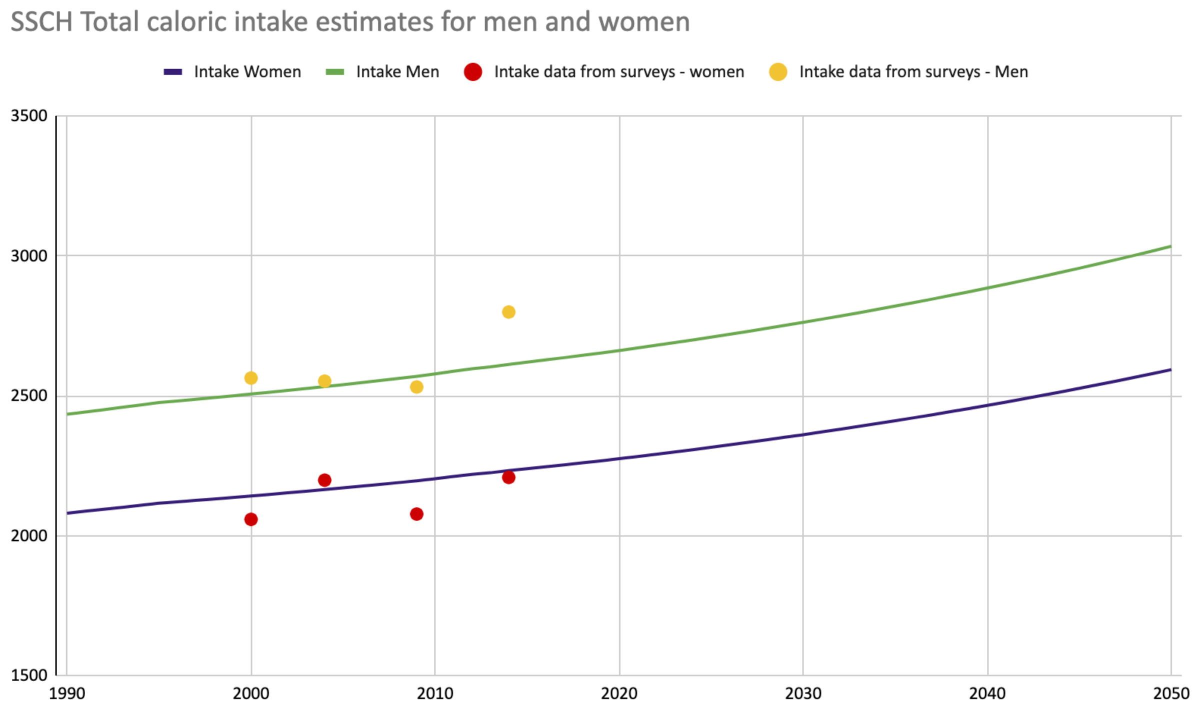

Figure S8.5 Structure for estimating caloric intake

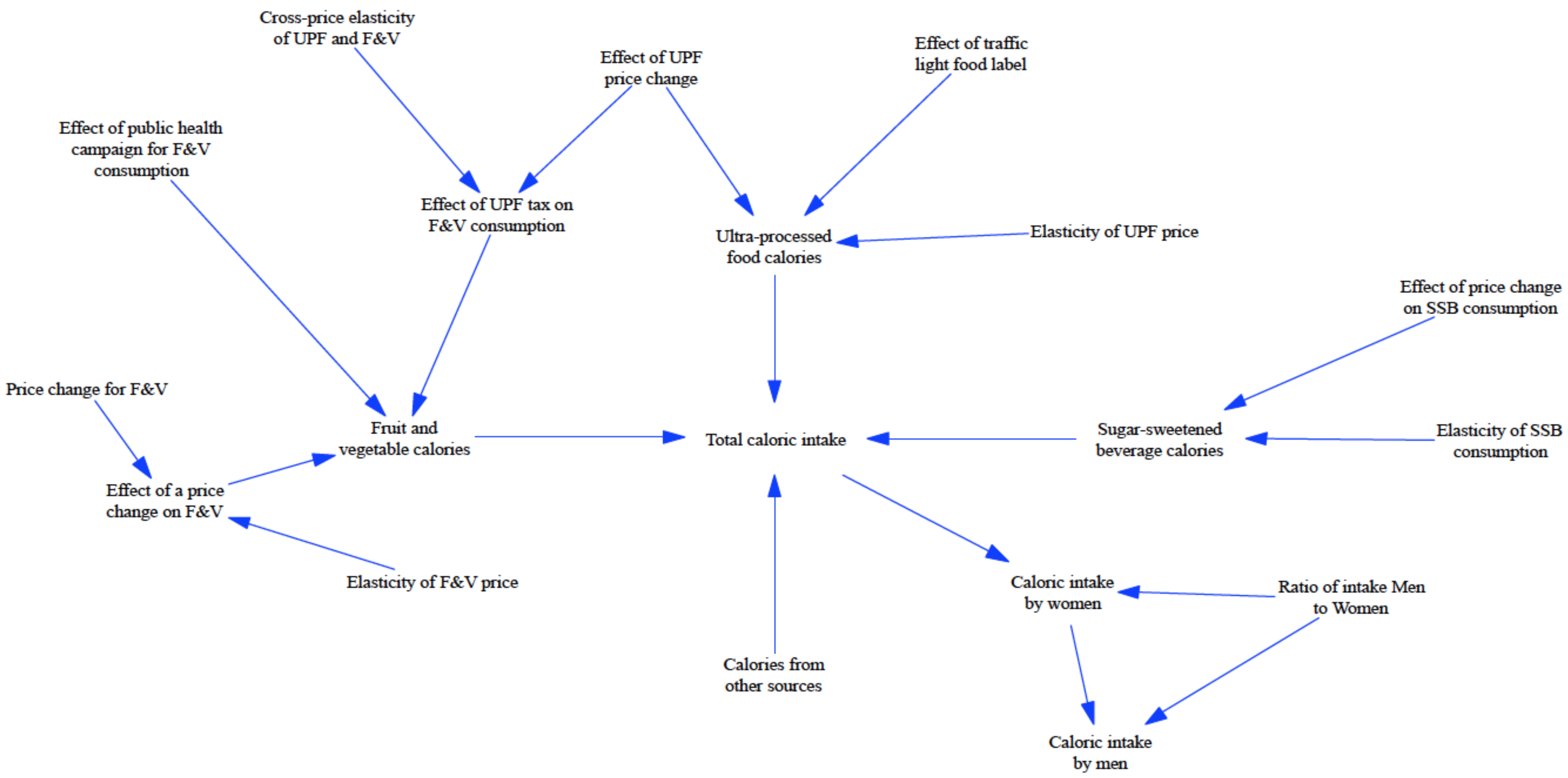

8.4 Equations to estimate caloric intake

$EffectPC_{FVConsumption}$ = FVPC * ElasFV

$Consumption_{FV+interventions}$ = $Consumption_{FV}$ + ($Consumption_{FV}$ * $EffectPC_{FVConsumption}$) + $EffectCampaign_{FV}$

$Kcal_{FV}$ = $Consumption_{FV+interventions}$ * 1 kcal/g

$EffectPC_{UPF}$ = UPFPC * ElasUPF

$Kcal_{UPF}$ = $Consumption_{UPF}$ + ($Consumption_{UPF}$ *$EffectPC_{UPF}$) - ($Consumption_{UPF}$ * $Effect_{FOPL}$)

$Kcal_{SSB}$ = SSBkcal * ($Consumption_{SSB}$ - (ElasSSB * SSBPC) * $Consumption_{SSB}$))

$Kcal_{Total}$ = $Kcal_{FV}$ + $Kcal_{UPF}$ + $Kcal_{SSB}$ + $Kcal_{Other}$

$Kcal_{Women}$ = $Kcal_{Total}$ / (FracWomen + (FracMen *RatioKcal))

$Kcal_{Men}$ = $Kcal_{Women}$ * RatioKcal

Price change is estimated as a percentage change in the price by the consumer, which is not always the same as a percentage tax (if it is a decrease, this should be a negative number)

Table S8.4 Variables to estimate caloric intake

Variable	Units	Description
$EffectPC_{FVConsumption}$	dimensionless	Proportion change in fruit and vegetable consumption as a result of a price change
FVPC	dimensionless	Proportion change in fruit and vegetable prices
ElasFV	dimensionless	Price elasticity of fruits and vegetables, constant set at -0.65(84)
$Consumption_{FV}$	g/person/day	Average daily consumption of fruits and vegetables per day over the course of a year
$EffectCampaign_{FV}$	g/person/day	Effect of a public health campaign on fruit and vegetable consumption, constant set at 4g/person/day on average over a year(85)
$Kcal_{FV}$	kcal/g	Calories consumed from fruits and vegetables (assumed to have 1kcal/g)
$EffectPC_{UPF}$	dimensionless	Proportion change in ultra-processed food consumption as a result of a price change
UPFPC	dimensionless	Proportion change on ultra-processed foods prices
ElasUPF	dimensionless	Price elasticity of ultra-processed foods, constant set at -0.9(86)
$Consumption_{UPF}$	kcal/day	Average calories consumed from ultra-processed foods per day in a year
$Effect_{FOPL}$	kcal/day	Proportional change in caloric intake from introducing front-of-package labelling for unhealthy foods, constant set at .0359(87)
(87)$Kcal_{SSB}$	kcal/day	Average calories consumed from sugar-sweetened beverages per day in a year
SSBkcal	kcal/unit	Average kcal per serving of SSBs, constant set at 130
$Consumption_{SSB}$	units/day	Average consumption of SSBs in units per day
ElasSSB	dimensionless	Price elasticity of SSBs, constant set at 1.7(88)
(88)SSBPC	dimensionless	Proportion change on SSB price
$Kcal_{Total}$	kcal/day	Total daily average caloric intake in a year
$Kcal_{Other}$	kcal/day	Daily caloric intake from other sources besides fruits and vegetables, ultra-processed foods, and sugar-sweetened beverages
$Kcal_{Women}$ / $Kcal_{Men}$	kcal/day	Average daily caloric intake for women / men in a year
RatioKcal	dimensionless	Ratio of difference in caloric intake of men to women. Constant set at 1.17

9 Calibration and baseline model predictions

We chose three major outcomes to measure for the SSCH model: diabetes prevalence, diabetes incidence, and obesity prevalence (with BMI estimates). It is possible to compare the prevalence for obesity and diabetes to survey data from Jamaica and the region. Incidence data are not available, although we do compare to the estimates from the Global Burden of Disease study (see section on diabetes onset). Calibration of some of the inputs was done to improve matching with the historical data that we do have. However, many of the assumptions are interdependent and we do not have strong data for all of the inputs. It may be that a data-driven adjustment in one input (caloric intake, for example) would affect the calibration of inputs in another area (physical activity) for which there are less data available.

9.1 Diabetes prevalence

The earliest survey results available for Jamaica that takes a nationally representative sample are from the Jamaica Health and Lifestyle survey - 2000 reported a prevalence of 7.2% based on a fasting blood glucose sample for ages 15 - 74 years (a sub-sample of the study had a venous fasting blood sample taken) using a point-of-care finger-prick device. The study reports an over-sampling of females and of older adults than younger, which could lead to an overestimation of diabetes, although the screening methods could lead to a slight underestimate as well. In general, the JHLS study estimates prevalence for 15-74 year olds, where the SSCH model is for 20+. One would expect, given that age is a risk factor for diabetes, that the prevalence will be slightly higher in the SSCH model than from the JHLS surveys.

Figure S9.1 Estimated diabetes prevalence from the SSCH model compared to measured estimates from studies

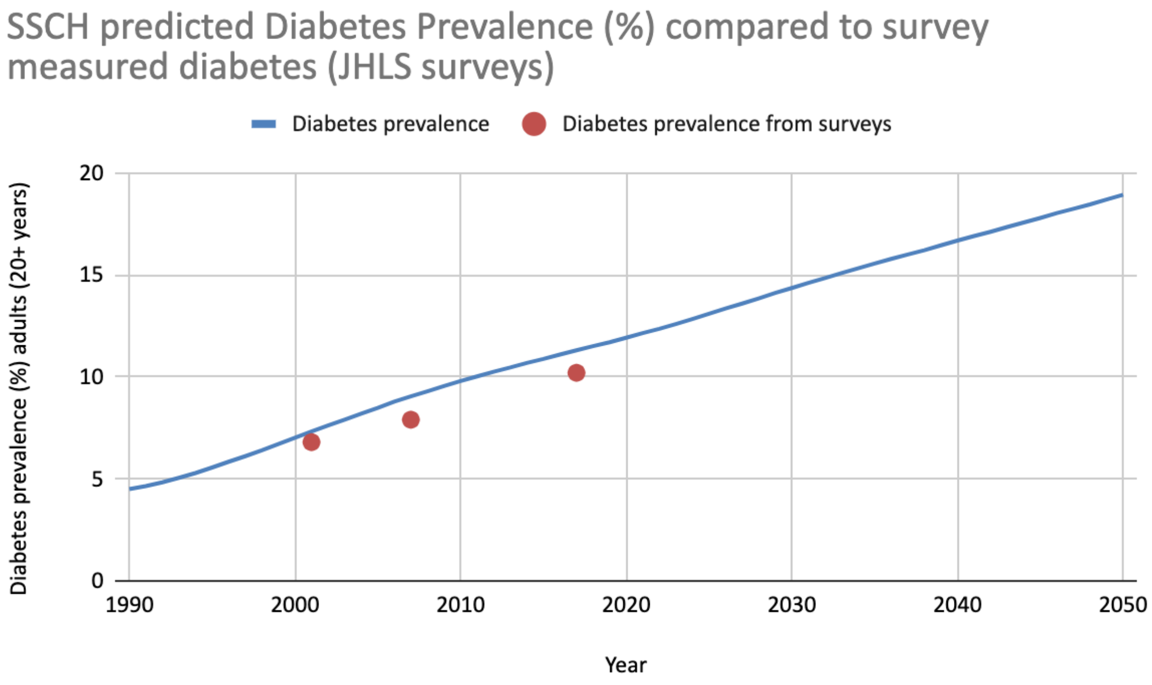

Figure S9.2 Estimated obesity prevalence from the SSCH model compared to measured estimates from studies

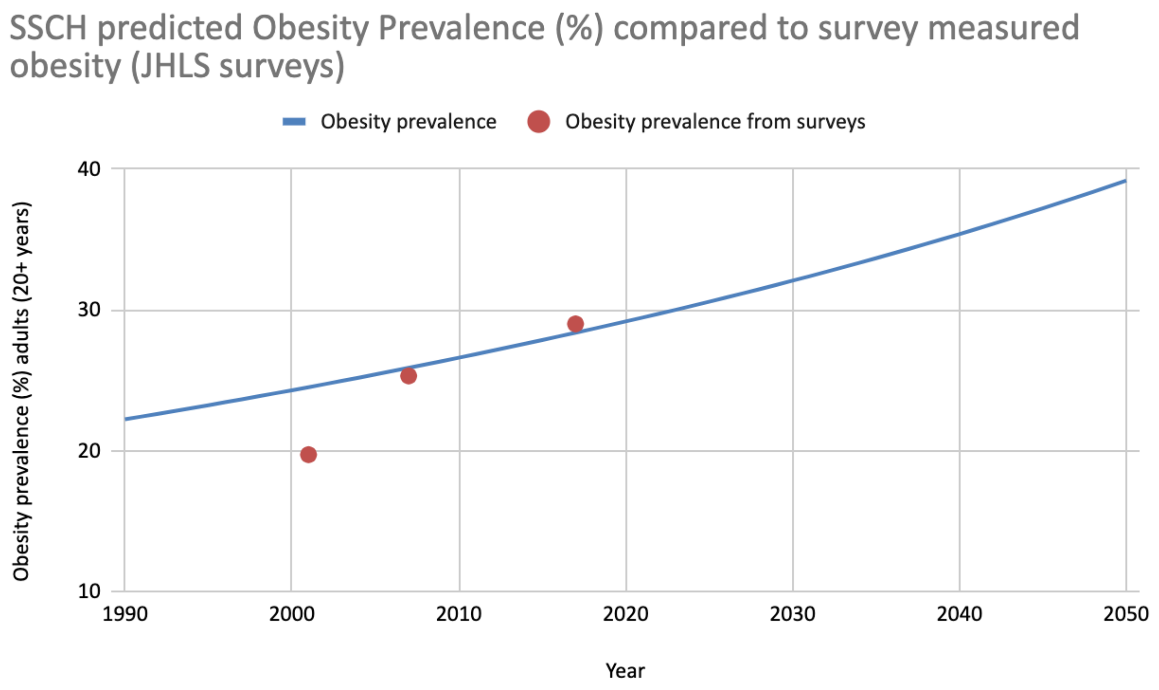

Figure S9.3 Estimated BMI from the SSCH model compared to measured estimates from studies

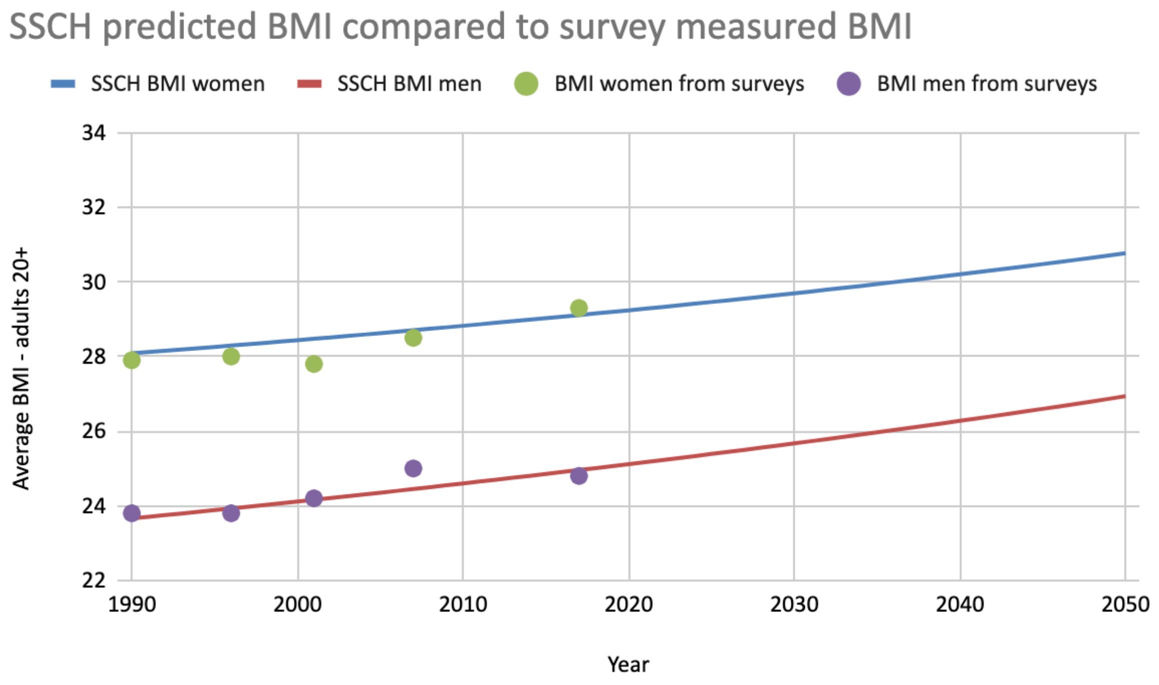

10 Scenario testing

We ran a series of scenarios that corresponded as closely as possible with priority interventions defined by stakeholders in the group model-building workshops in Jamaica(28,29). These are presented in Table 11.1 organized by broad domains for diet, physical activity, and care to people with diabetes. The full workshop report including causal diagrams is available upon request to the authors.

Table S10.1. Stakeholder-identified priority areas for intervention simulation

Policies for evaluation from a systems science perspective		
**Unhealthy Diet**	**Physical Activity**	**Care to people with diabetes**
1. Cost and availability of fruit and vegetables	1. Workplace promotion and facilities for physical activity	1. Improve treatment to target on blood glucose, blood pressure, and lipids
2. Mandatory nutrition standards for school foods and eating patterns	2. Investment into active transport and leisure infrastructure	2. Universal access to essential medicines for diabetes
3. A tax on sugar-sweetened beverages and other processed, unhealthy foods	3. Parking restrictions and other fiscal measures to reduce car use	3. Increase screening of people at high risk to diagnose pre-diabetes and early diabetes
		4. Implementation of a multi-disciplinary team model for treatment

To meet these priorities, we reviewed the literature, prioritizing studies conducted in the Caribbean, and in the absence of those, pooled estimates from systematic reviews to simulate the impact of the interventions alone and in combination. For some interventions, there was not enough evidence to adequately understand the impact of implementation. We then developed complementary scenarios based on other interventions discussed in the workshop. Those scenarios and their effect sizes are presented in Table 11.2.

We did not model effects of changes on fruit and vegetable intake for obesity. In the way the model is constructed, any increase in fruit and vegetable intake would increase caloric intake and thus increase weight gain. There is evidence that this is not the case and that at the very least, increases in fruit and vegetable intake do not lead to weight gain and may, in fact, lead to weight loss(90,91). We also included front-of-package labeling as well since the Healthy Caribbean Coalition has been calling for them to be adopted around the Caribbean.

The effect sizes from the literature are limited in their applicability to the Caribbean because most were developed in other settings or using pooled estimates. Nevertheless, interventions targeting sugar sweetened beverages and ultra processed foods have the largest impact on diabetes and obesity prevalence.

Table S10.2. A summary of literature-based scenarios for testing in the SSCH model and 30-year effects on diabetes and obesity prevalence

Intervention	Details	Effect size	Change in diabetes prevalence by 2050	Change in obesity prevalence by 2050
Community-based physical activity interventions	Meta-analysis of interventions at the community level to improve physical activity in the sedentary or those at high-risk for type 2 diabetes.	Adds a median 25 MVPA minutes per week(92)	-0.16 (0.7%)	-0.06 (0.1%)
Development of walking and cycling routes	Development of a cycling and walking path in a middle-income urban setting.	Adds a mean 15 min/week of MVPA(93); Effects are only seen for those living within 500m of the development.	-0.09 (0.5%)	-0.04 (0.1%)
Development of public transport routes	Pooled physical activity time that comes from adding a new public transport route (bus, tram, or train)	1.76 MET-hours per week(65)	-0.13 (0.6%)	-0.05 (0.1%)
Public information campaign to increase physical activity	Mass media physical activity campaign	Pooled relative risk increased MVPA in those targeted by 28%(66)	-0.72 (3%)	-0.3 (0.7%)
Interventions to reduce sedentary time	Pooled reduction in mean sedentary time from interventions mostly focused the workplace	Mean reduction of 91 min/day of sedentary time(94)	-0.56 (2.7%)	-0.23 (0.58%)
Changes in price of sugar sweetened beverages	Increase the price of sugar sweetened beverages by 20% (minimum recommended increase(95))	Price elasticity of -1.3, pooled estimate from a systematic review(88)	-0.89 (4.3%)	-0.39 (1%)
Changes in price of unhealthy foods	Increase the price of unhealthy foods by 20% (assumed to be ultra processed foods excluding SSBs)	A pooled estimate of 10% increase in price lead to a 9% decrease in unhealthy foods(86)	-0.07 (0.3%)	-0.87 (2.2%)
Changes in price of fruits and vegetables	Decrease price of fruits and vegetables by 20%	Pooled estimate for middle income countries on price elasticity of -0.65 of fruits and vegetables(84)	-0.05 (0.2%)	Not modeled
Public information campaign on fruit and vegetable consumption	Mass media public information campaign on fruit and vegetable intake	Up to 4g/day increase in consumption of fruits and vegetables from an economic model(85)	-0.004 (0.01%)	Not modeled
Front-of-package warning labels (FOPL)	Adopt FOPL of the nutrition warning label type, similar to those used in Chile(83)	FOPLs lead to a 6.6% total calorie reduction, 13% increase in fruit and vegetable intake and a 13% decrease in UPF(96)	-0.09 (0.4%)	-0.75 (1.9%)

10.1 Magnitude change of changing individual inputs

In addition to exploring effect sizes drawn from the scientific literature, we modeled a number of scenarios for magnitude changes in physical activity and diet. To estimate these changes we applied a magnitude change to the existing baseline trends starting in 2020 and projecting to 2050. These are presented below as dose-response curves where there are incremental changes to the determinant. The annual change compared to the baseline curve is plotted over time from the intervention year (set as 2020) and projected forward 30 years. The curves are presented below in Figure 10.1a-i.

Not surprisingly, magnitude increases or decreases in physical activity or components of diet yield changes in the outcomes of a similar magnitude. What is perhaps more meaningful is that the impact of many of the changes is not linear, especially for diabetes prevalence. This is mostly likely to do with other mechanisms at play including the aging of the population, and feedbacks like the balancing loop for weight gain that eventually stabilizes at a new equilibrium BMI and basal metabolic rate once there is a change. The central conclusion from all of these curves is the importance of sustaining interventions or even attempting to increase changes over time.

Changes to obesity prevalence are more linear in nature, but these, of course, have effects on pre-diabetes and diabetes onset as well. The magnitude of change for these is also less pronounced than those for diabetes prevalence. The reductions in obesity are found from the reduction of consumption of ultra-processed foods which currently make up an estimated 30-40% of the Caribbean diet(97). That relationship is nonlinear so that greater reductions in intake lead to greater reductions in prevalence over time.

For downstream interventions, a reduction in diabetes mortality from the uptake of diabetes self-management education leads to an increase in diabetes prevalence, all things being equal. However, these clearly represent a positive outcome in terms of public health though they may offset reductions from upstream interventions. Targeting people at high risk of diabetes can also lead to substantial reductions in diabetes prevalence but much depends on the proportion of the population that is identified and adheres to an intervention. Even 25% of the pre-diabetes population following a strict lifestyle intervention administered through the health system is aspirational and unlikely to be possible on a great scale.

Figure S10.1 a-i. Dose-response curves for magnitude changes in physical activity, diet, and health system interventions

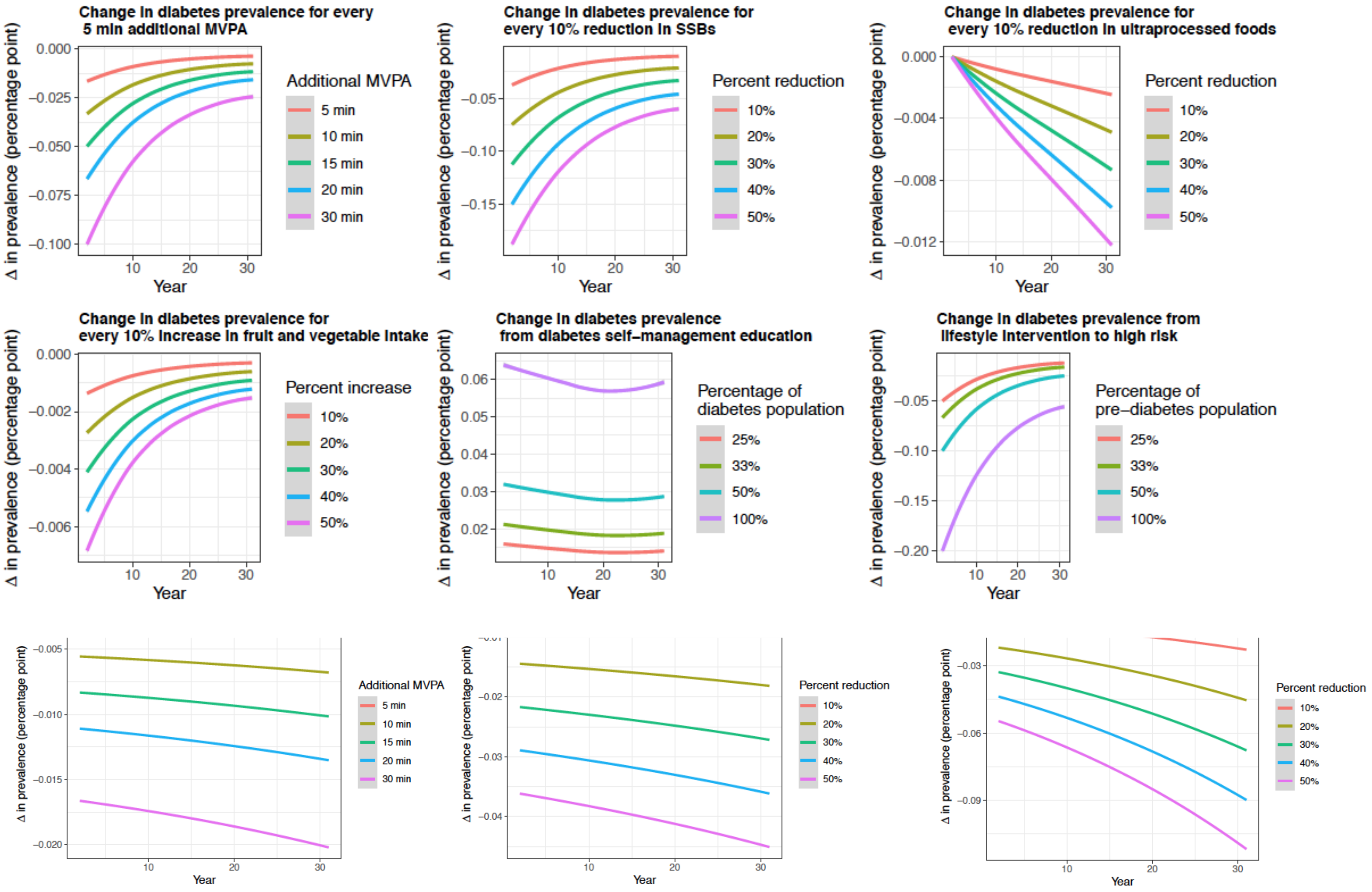

10.2 Aspirational scenarios

We also ran a series of scenarios that are unrealistic and largely aspirational to understand the impact of shifting certain patterns that are typical of the Caribbean including a large number of sedentary hours, women being more than three times less physically active than men, the effect of replacing ultra-processed foods with fruits and vegetables, and the impact of different levels of weight loss at the population level. These are presented in Table 11.3.

Table S10.3 Aspirational scenarios and their impacts on diabetes and obesity prevalence

Scenario	Change in diabetes prevalence p.p (%)		Change in obesity prevalence p.p. (%)	
	2030	2050	2030	2050
Baseline prediction	15.4	20.9	32.1	39.2
Replace 4 hours of sedentary time with light PA	-0.8 (-5.2%)	-1.6 (-7.6%)	-0.2 (-0.6%)	-0.6 (-1.5%)
Women as physically active as men	-0.9 (-5.8%)	-1.5 (-7.2%)	-0.3 (-0.9%)	-0.7 (-1.8%)
Replace all ultraprocessed foods with fruit and vegetables	-0.1 (-0.6%)	-0.3 (-1.4%)	NA	NA
No increase in obesity prevalence after 2020	-0.1 (0.6%)	-0.9 (-4.3%)	NA	NA
Weight loss of 500g per year for the whole adult population	-0.5 (-3.2%)	-3.9 (-18.7%)	-12.6 (-39.2%)	-34.4 (-87.7%)
Weight loss of 500g for women only	-0.4 (-2.4%)	-2.6 (-12.4%)	-8.5 (-26.5%)	-12.9 (32.9%)

The purpose of these scenarios is mostly illustrative as they are extreme and highly unlikely. However, they show that for lowering diabetes prevalence, increasing physical activity is critical and that intervening in women, who are more physically inactive and more obese than men, could yield significant results at the population level.

There are also benefits to reducing sedentary time and replacing it even with light physical activity(94) although the amount of reduction is unlikely to be as great as the one we model here.

10.2 Achieving global targets

We also used the model to explore the necessary changes in diet and physical activity to achieve targets set by the WHO Global Action Plan on NCDs to stop the rise in obesity and diabetes prevalence by 2025 (using 2010 as a starting reference). A detailed discussion of

this analysis is presented in the main paper. Briefly, we explored different magnitude changes in increasing MVPA (doubling, then tripling) and decreasing caloric intake (by 20% and 30%) on diabetes and obesity prevalence. The initial analysis maintains future trends in physical activity and caloric intake so that it is just a magnitude change that is applied to the baseline trend. The results are presented in the figures below (see Figure 11.2, same as Figure 3 in the main text).

Figure S10.2 Impact of changes in caloric intake and MVPA on achieving global targets to stop the rise in diabetes and obesity prevalence in adults.

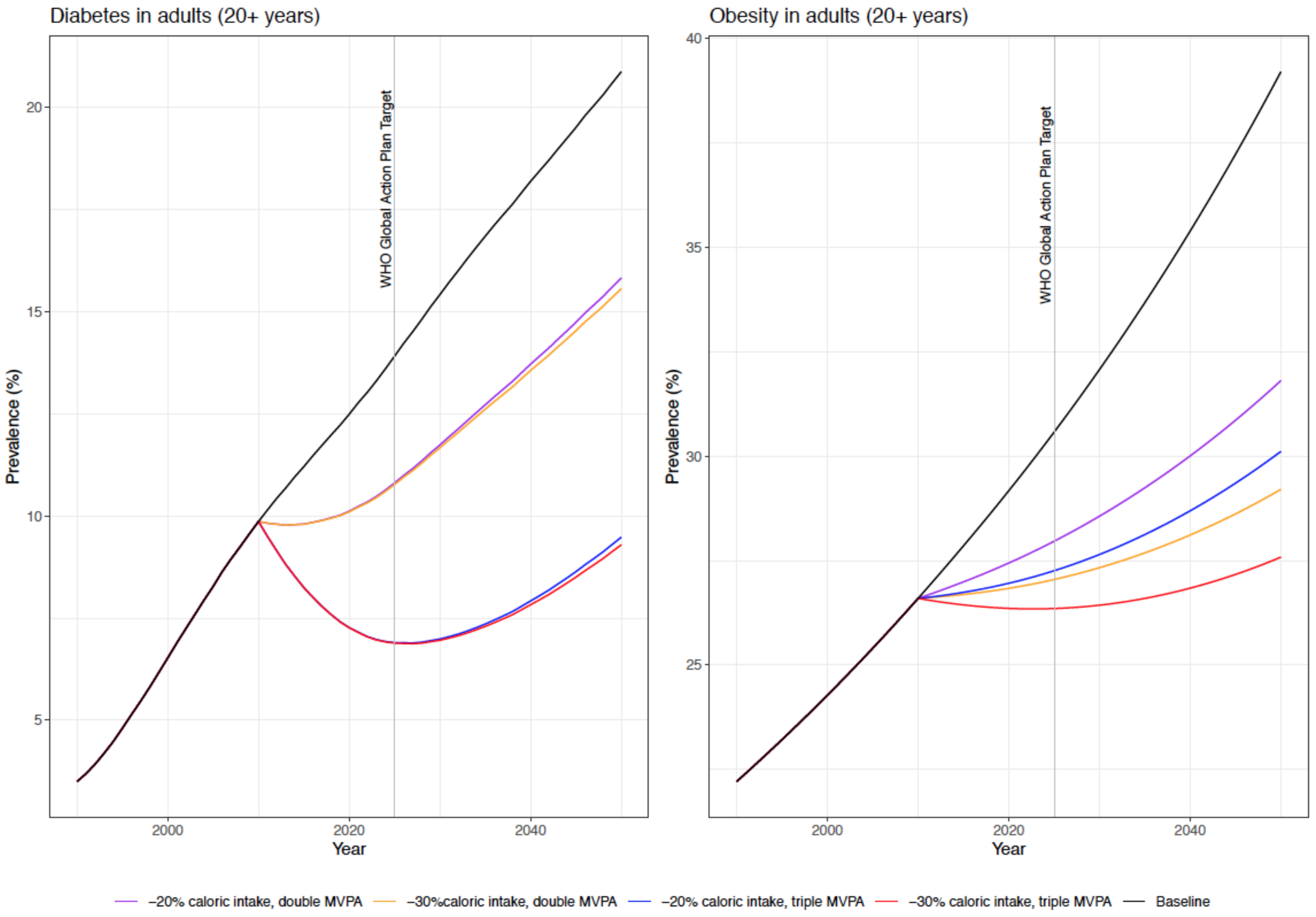

We also ran a parallel analysis which did not maintain trends but instead applied a static difference to MVPA and caloric intake from the value used in 2010 and maintains that value through to 2025. The results of those scenarios are presented in Figure 11.3. We see from the figures that regardless of projecting trends forward, the only scenario that manages to achieve the target of stopping the rise in diabetes prevalence is the most extreme of tripling MVPA and reducing caloric intake by 30%. The trends only start to cause a rise in diabetes prevalence following the year 2025. For obesity the impact is not as dramatic, but again, the only scenario that achieves it is the most extreme, regardless of trends.

Figure S10.3 Analysis of global targets with no decreasing trend in MVPA or increasing trend in caloric intake

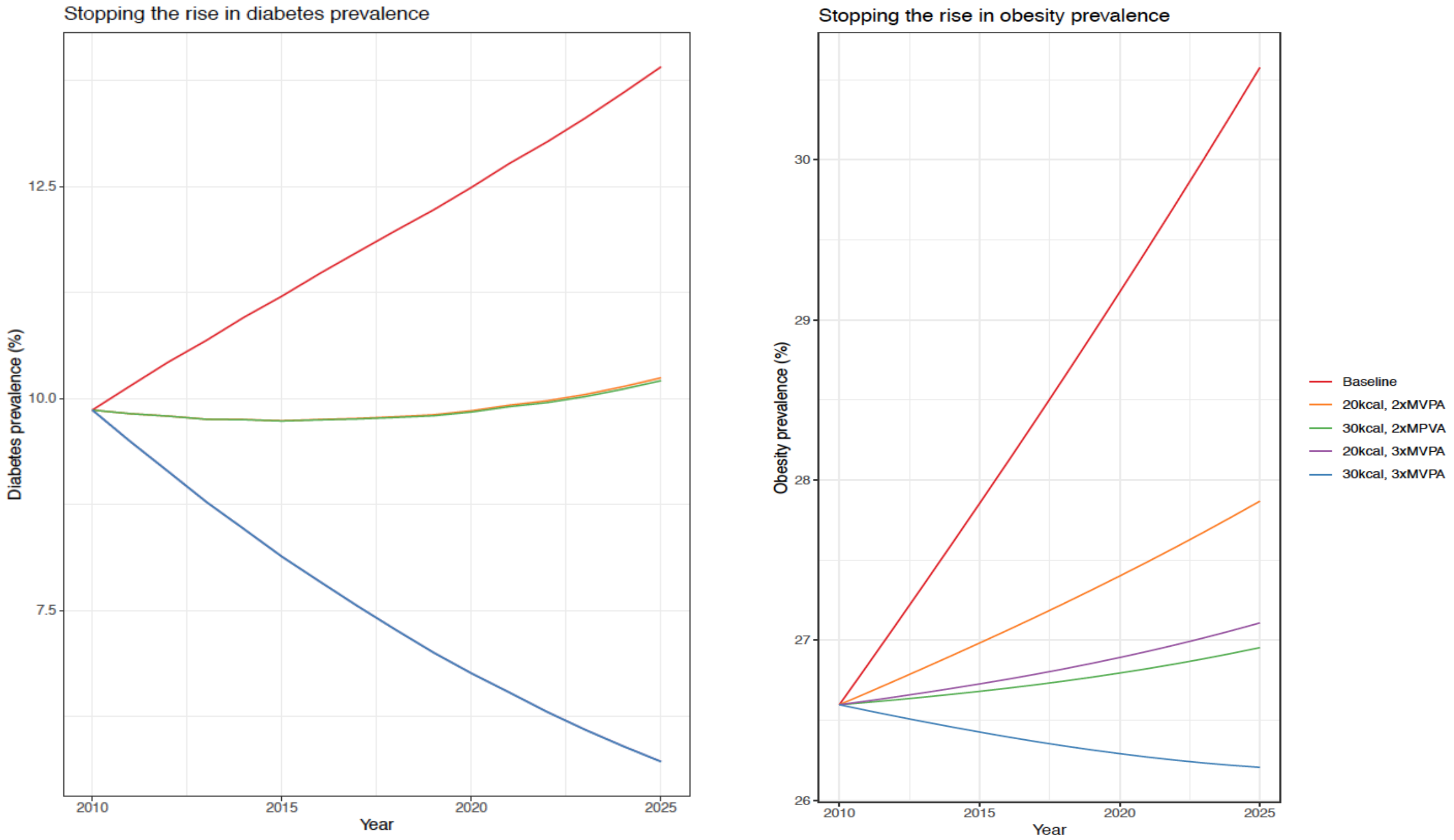

11 Sensitivity analyses

We carried out a multi-variable as well as univariable sensitivity analysis. We chose ranges corresponding to the values reported in the literature for the effect sizes where they were available. Where no confidence intervals or certainty limits were available, we varied within a range of 20% more or less than the chosen value as recommended by Sterman et al.

Table S11.1 Ranges for the sensitivity analyses

Variable	baseline	min	max
Baseline all-cause mortality rate in people under 55 (adults/1000/year)	2.09	1.94	2.26
Baseline all-cause mortality rate in people over 55 (adults/1000/year)	31.96	30.11	33.97
Pre-diabetes onset rate in the non-obese (adults/1000/year)	22	15	40.8
Relative risk of prediabetes onset in the obese	1.72	1.3	1.7
Relative risk of prediabetes onset in the over 55	1.2	1	1.5
Proportion of remission from prediabetes per year (%)	7	5	10
Diabetes onset rate from prediabetes (adults/1000/year)	50	30	65
Relative risk of diabetes onset in the obese	3.9	2.3	4.5
Relative risk of diabetes per unit serving of SSBs (%)	26	11	43
Relative risk of diabetes onset in the over 55	2.44	2	2.6
Relative risk of all-cause mortality for people with diabetes over 55	1.6	1.4	2.6
Relative risk of all-cause mortality for people with diabetes under 55	3.5	2	4.6

11.1 Multivariable sensitivity analysis of epidemiological assumptions

We used Latin hypercube sampling(89) to select values within minimum and maximum ranges for the variables above. The method samples values for parameters within ranges using a multidimensional distribution that yields a near-random sample that can more accurately reflect the variability in a real-world scenario than a simple random sample. We used a sample of 100 runs and ran each of these scenarios through the model to explore the variability in the predictions of the main outcome variable: diabetes prevalence. The majority of the runs show a clustering near the predicted trends of around 19% with a few extreme scenarios of up to 30% diabetes prevalence in 2050 and as low as 12% prevalence.

Figure S11.1 Multivariate sensitivity analyses effect on diabetes prevalence estimates

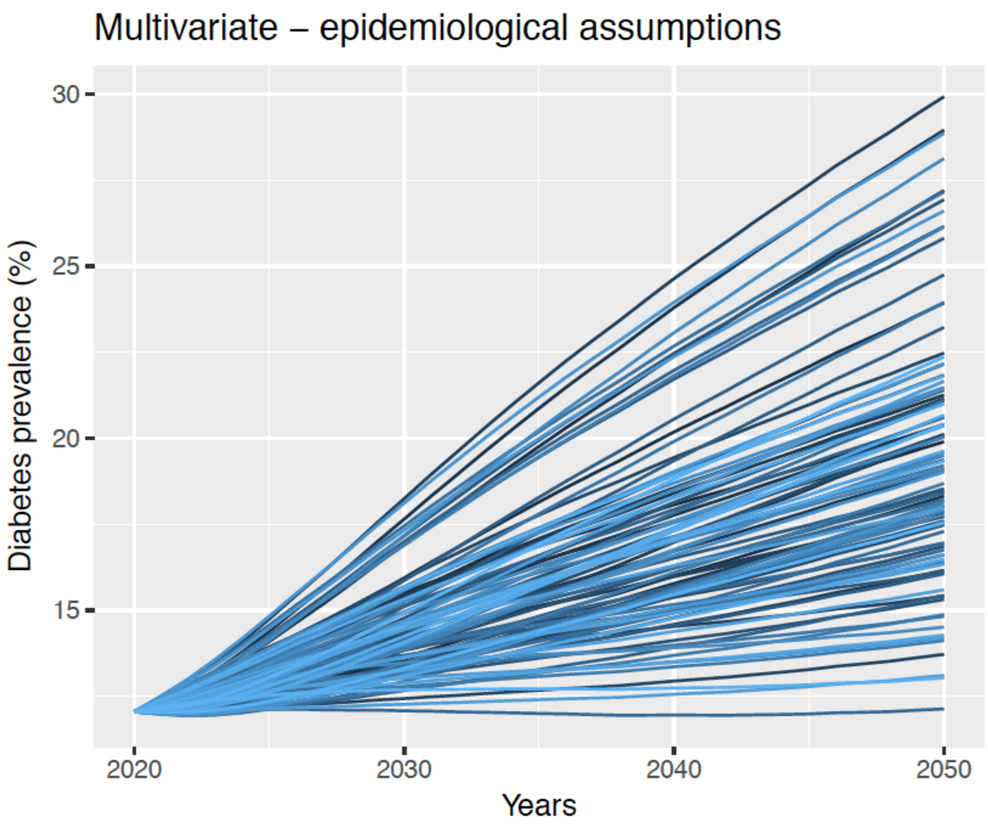

11.2 Univariable sensitivity analysis of epidemiological assumptions

We also explored the effect of individually varying the baseline assumptions for some of the key input variables using latin hypercube sampling to select within the ranges above for one variable at a time.

Diabetes prevalence is most sensitive to the baseline assumptions for prediabetes incidence and diabetes incidence rates. There are many factors that influence these rates and this model only considers a few. It is not surprising that these key flow rates are the most important determinants of the main outcome. The rates chosen calibrate well with historical data from population-based surveys of diabetes prevalence, but in the absence of longitudinal data it is impossible to know the precision of the rates to the true incidence. The only other two variables that have a larger influence on the outcome are the prediabetes recovery rate, which is also an important determinant of the stock of prediabetes and so has an influence on the diabetes stock as well. Finally, the relative risk of mortality in people with diabetes over 55 also influences the diabetes stock. Because the population of people with diabetes is older than the total population, it is not surprising that changes in mortality in this fraction of the population would influence the outflow from the diabetes stock. Varying SSB consumption, one of the only determinants linked to diabetes incidence, also has an impact on the diabetes stock and because of the relatively high relative risk (1.26) related to a unit consumption, can have a large impact on diabetes incidence and thus prevalence.

Figures S11.2a-o Univariate sensitivity analyses effect on diabetes prevalence

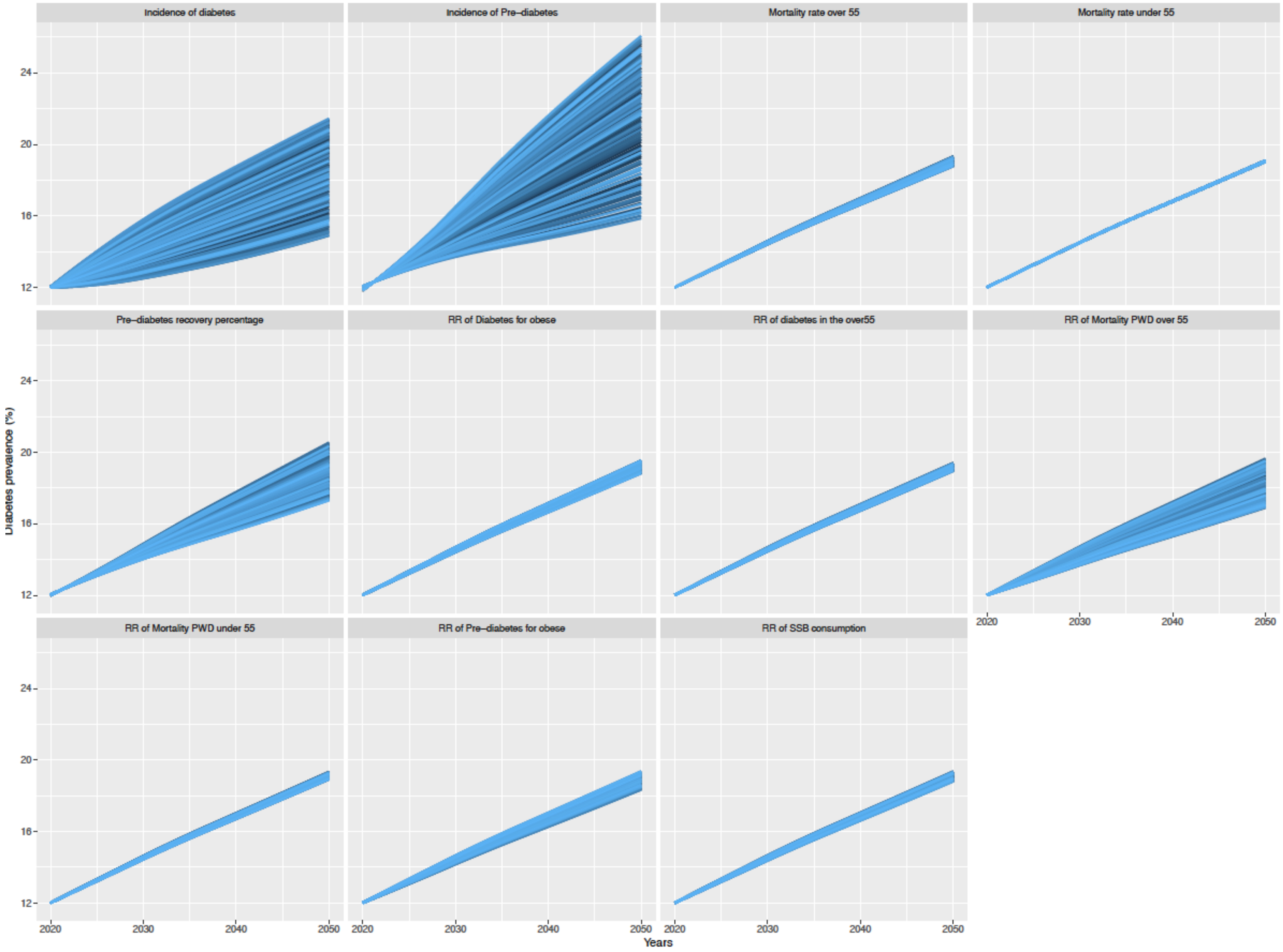

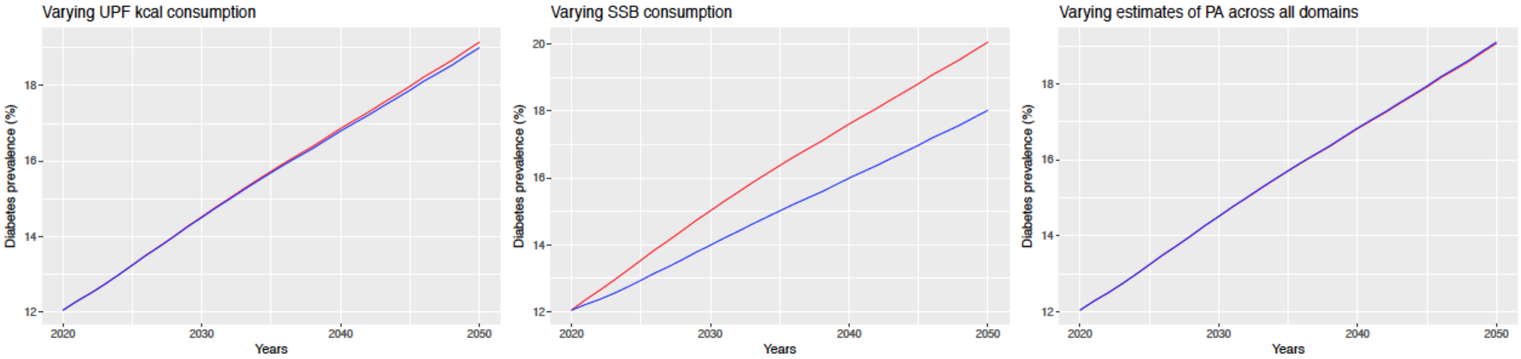
Varying UPF kcal consumption
Varying SSB consumption
Varying estimates of PA across all domains
Diabetes prevalence (%)
Years
2020
2030
2040
2050
12
14
16
18
20

References

1. Monks T, Currie CSM, Onggo BS, Robinson S, Kunc M, Taylor SJE. Strengthening the reporting of empirical simulation studies: Introducing the STRESS guidelines. Journal of Simulation. 2019 Jan 2;13(1):55–67.
2. Taylor R, Ramachandran A, Yancy WS, Forouhi NG. Nutritional basis of type 2 diabetes remission. BMJ. 2021 Jul 7;n1449.
3. World Population Prospects 2019 [Internet]. United Nations, Department of Economic and Social Affairs, Population Division; 2019 [cited 2019 Jul 1]. Available from: https://population.un.org/wpp/DataQuery/
4. Jones AP, Homer JB, Murphy DL, Essien JDK, Milstein B, Seville DA. Understanding Diabetes Population Dynamics Through Simulation Modeling and Experimentation. Am J Public Health. 2006 Mar;96(3):488–94.
5. GBD Results Tool | GHDx [Internet]. [cited 2019 May 8]. Available from: http://ghdx.healthdata.org/gbd-results-tool
6. Roglic G, Unwin N, Bennett PH, Mathers C, Tuomilehto J, Nag S, et al. The burden of mortality attributable to diabetes: realistic estimates for the year 2000. Diabetes Care. 2005 Sep;28(9):2130–5.
7. Gu K, Cowie CC, Harris MI. Mortality in Adults With and Without Diabetes in a National Cohort of the U.S. Population, 1971–1993. Diabetes Care. 1998 Jul 1;21(7):1138–45.
8. Gregg EW, Cheng YJ, Srinivasan M, Lin J, Geiss LS, Albright AL, et al. Trends in cause-specific mortality among adults with and without diagnosed diabetes in the USA: an epidemiological analysis of linked national survey and vital statistics data. The Lancet. 2018 Jun;391(10138):2430–40.
9. Chen L, Islam RM, Wang J, Hird TR, Pavkov ME, Gregg EW, et al. A systematic review of trends in all-cause mortality among people with diabetes. Diabetologia. 2020 Sep;63(9):1718–35.
10. Hambleton IR, Jeyaseelan S, Howitt C, Sobers-Grannum N, Hennis AJ, Wilks RJ, et al. Cause-of-death disparities in the African diaspora: exploring differences among shared-heritage populations. Am J Public Health. 2015 Jul;105 Suppl 3:S491-498.
11. Hambleton IR, Howitt C, Jeyaseelan S, Murphy MM, Hennis AJ, Wilks R, et al. Trends in Longevity in the Americas: Disparities in Life Expectancy in Women and Men, 1965-2010. Chang C-K, editor. PLoS ONE. 2015 Jun 19;10(6):e0129778.
12. World Health Organization, International Diabetes Federation. Definition and diagnosis of diabetes mellitus and intermediate hyperglycaemia: report of a WHO/IDF consultation [Internet]. 2006 [cited 2021 Sep 30]. Available from: http://www.who.int/diabetes/publications/diagnosis_diabetes2006/en/
13. American Diabetes Association. Diagnosis and Classification of Diabetes Mellitus. Diabetes Care. 2014 Jan 1;37(Supplement_1):S81–90.
14. Ministry of Health and Social Development. STEPS Risk Factor Survey Report (BVI) [Internet]. British Virgin Islands; 2010 Aug. Available from: https://cdn.who.int/media/docs/default-source/ncds/ncd-surveillance/data-reporting/british-virgin-islands/steps/2009_bvi_steps_report-vi.pdf?sfvrsn=18efcacc_5&download=true
15. Dominica Ministry of Health. Dominica STEPS Survey 2008 Fact Sheet [Internet]. 2008. Available from: https://cdn.who.int/media/docs/default-source/ncds/ncd-surveillance/data-reporting/dominica/steps/dominica_2008_steps_factsheet.pdf?sfvrsn=b1ee05e4_5&download=true
16. Belize Ministry of Health, The Centers for Disease Control and Prevention, Pan American Health Organization. The Central America Diabetes Initiative (CAMDI): Survey of Diabetes, Hypertension and Chronic Disease Risk Factors, Belize [Internet]. 2009. Available from: https://iris.paho.org/handle/10665.2/7687
17. Jamaica Health and Lifestyle Survey III (2016-2017) Preliminary Key Findings [Internet]. Kingston, Jamaica: Tropical Medicine Research Institute, University of the

West Indies; 2018. Available from: https://www.moh.gov.jm/wp-content/uploads/2018/09/Jamaica-Health-and-Lifestyle-Survey-III-2016-2017.pdf
18. Unwin N, Rose A, George K, Hambleton IR, Howitt C. The Barbados Health of the Nation Survey: Core Findings. St Michael, Barbados: Chronic Disease Research Centre, The Unviersity of the West Indies and the Barbados Ministry of Health; 2015 Jan.
19. Cowie CC, Rust KF, Ford ES, Eberhardt MS, Byrd-Holt DD, Li C, et al. Full Accounting of Diabetes and Pre-Diabetes in the U.S. Population in 1988-1994 and 2005-2006. Diabetes Care. 2009 Feb 1;32(2):287–94.
20. Wilks RJ, Younger N, Tulloch-Reid MK, McFarlane S, Francis D. Jamaica Health and Lifestyle Survey II 2007–8 [Internet]. Kingston, Jamaica: Tropical Medicine Research Institute, University of the West Indies; 2008. Available from: https://www.moh.gov.jm/wp-content/uploads/2015/05/Jamaica-Health-and-Lifestyle-Survey-2007-8.pdf
21. On behalf of EDEG, Forouhi NG, Balkau B, Borch-Johnsen K, Dekker J, Glumer C, et al. The threshold for diagnosing impaired fasting glucose: a position statement by the European Diabetes Epidemiology Group. Diabetologia. 2006 May;49(5):822–7.
22. Davidson MB, Landsman PB, Alexander CM. Lowering the Criterion for Impaired Fasting Glucose Will Not Provide Clinical Benefit. Diabetes Care. 2003 Dec 1;26(12):3329–30.
23. Borch-Johnsen K, Colagiuri S, Balkau B, Gl□mer C, Carstensen B, Ramachandran A, et al. Creating a pandemic of prediabetes: the proposed new diagnostic criteria for impaired fasting glycaemia. Diabetologia [Internet]. 2004 Aug [cited 2021 Sep 30];47(8). Available from: http://link.springer.com/10.1007/s00125-004-1468-6
24. Gerstein HC, Santaguida P, Raina P, Morrison KM, Balion C, Hunt D, et al. Annual incidence and relative risk of diabetes in people with various categories of dysglycemia: A systematic overview and meta-analysis of prospective studies. Diabetes Research and Clinical Practice. 2007 Dec 1;78(3):305–12.
25. Xu XY, Leung AYM, Smith R, Wong JYH, Chau PH, Fong DYT. The relative risk of developing type 2 diabetes among individuals with prediabetes compared with individuals with normoglycaemia: Meta-analysis and meta-regression. J Adv Nurs. 2020 Dec;76(12):3329–45.
26. Neuenschwander M, Ballon A, Weber KS, Norat T, Aune D, Schwingshackl L, et al. Role of diet in type 2 diabetes incidence: umbrella review of meta-analyses of prospective observational studies. BMJ. 2019 Jul 3;366:l2368.
27. Smith AD, Crippa A, Woodcock J, Brage S. Physical activity and incident type 2 diabetes mellitus: a systematic review and dose–response meta-analysis of prospective cohort studies. Diabetologia. 2016 Dec 1;59(12):2527–45.
28. Guariguata L, Rouwette EA, Murphy MM, Saint Ville A, Dunn LL, Hickey GM, et al. Using Group Model Building to Describe the System Driving Unhealthy Eating and Identify Intervention Points: A Participatory, Stakeholder Engagement Approach in the Caribbean. Nutrients. 2020 Jan 31;12(2):384.
29. Guariguata L, Unwin N, Garcia L, Woodcock J, Samuels TA, Guell C. Systems science for developing policy to improve physical activity, the Caribbean. Bulletin of the World Health Organization. 2021;Accepted.
30. Lindstrom J, Tuomilehto J. The Diabetes Risk Score: A practical tool to predict type 2 diabetes risk. Diabetes Care. 2003 Mar 1;26(3):725–31.
31. Tillin T, Sattar N, Godsland IF, Hughes AD, Chaturvedi N, Forouhi NG. Ethnicity-specific obesity cut-points in the development of Type 2 diabetes - a prospective study including three ethnic groups in the United Kingdom. Diabet Med. 2015 Feb;32(2):226–34.
32. Nichols GA, Schroeder EB, Karter AJ, Gregg EW, Desai J, Lawrence JM, et al. Trends in Diabetes Incidence Among 7 Million Insured Adults, 2006–2011. Am J Epidemiol. 2015 Jan 1;181(1):32–9.
33. Tabaei B, Chamany S, Driver C, Kerker B, Silver L. Incidence of Self-Reported

Diabetes in New York City, 2002, 2004, and 2008. Prev Chronic Dis [Internet]. 2012 Jun [cited 2021 Sep 30]; Available from: http://www.cdc.gov/pcd/issues/2012/11_0320.htm
34. Consortium TI. Long-Term Risk of Incident Type 2 Diabetes and Measures of Overall and Regional Obesity: The EPIC-InterAct Case-Cohort Study. PLOS Medicine. 2012 Jun 5;9(6):e1001230.
35. Magliano DJ, Islam RM, Barr ELM, Gregg EW, Pavkov ME, Harding JL, et al. Trends in incidence of total or type 2 diabetes: systematic review. BMJ. 2019 Sep 11;l5003.
36. Anjana RM, Rani CSS, Deepa M, Pradeepa R, Sudha V, Nair HD, et al. Incidence of Diabetes and Prediabetes and Predictors of Progression Among Asian Indians: 10-Year Follow-up of the Chennai Urban Rural Epidemiology Study (CURES). Diabetes Care. 2015 Aug 1;38(8):1441–8.
37. Vijayakumar G, Manghat S, Vijayakumar R, Simon L, Scaria LM, Vijayakumar A, et al. Incidence of type 2 diabetes mellitus and prediabetes in Kerala, India: results from a 10-year prospective cohort. BMC Public Health. 2019 Dec;19(1):140.
38. Latifi SM, Karandish M, Shahbazian H, Hardani Pasand L. Incidence of Prediabetes and Type 2 Diabetes among People Aged over 20 Years in Ahvaz: A 5-Year Perspective Study (2009–2014). Journal of Diabetes Research. 2016;2016:1–6.
39. Kolberg JA, Jorgensen T, Gerwien RW, Hamren S, McKenna MP, Moler E, et al. Development of a Type 2 Diabetes Risk Model From a Panel of Serum Biomarkers From the Inter99 Cohort. Diabetes Care. 2009 Jul 1;32(7):1207–12.
40. Vaidya A, Cui L, Sun L, Lu B, Chen S, Liu X, et al. A prospective study of impaired fasting glucose and type 2 diabetes in China. Medicine (Baltimore) [Internet]. 2016 Nov 18 [cited 2021 Mar 9];95(46). Available from: https://www.ncbi.nlm.nih.gov/pmc/articles/PMC5120921/
41. Ligthart S, van Herpt TTW, Leening MJG, Kavousi M, Hofman A, Stricker BHC, et al. Lifetime risk of developing impaired glucose metabolism and eventual progression from prediabetes to type 2 diabetes: a prospective cohort study. Lancet Diabetes Endocrinol. 2016 Jan;4(1):44–51.
42. Kowall B, Rathmann W, Strassburger K, Meisinger C, Holle R, Mielck A. Socioeconomic status is not associated with type 2 diabetes incidence in an elderly population in Germany: KORA S4/F4 Cohort Study. Journal of Epidemiology & Community Health. 2011 Jul 1;65(7):606–12.
43. Owei I, Umekwe N, Provo C, Wan J, Dagogo-Jack S. Insulin-sensitive and insulin-resistant obese and non-obese phenotypes: role in prediction of incident pre-diabetes in a longitudinal biracial cohort. BMJ Open Diab Res Care. 2017 Jul;5(1):e000415.
44. Hadaegh F, Derakhshan A, Zafari N, Khalili D, Mirbolouk M, Saadat N, et al. Pre-diabetes tsunami: incidence rates and risk factors of pre-diabetes and its different phenotypes over 9 years of follow-up. Diabet Med. 2017 Jan;34(1):69–78.
45. Zhu Y, Sidell MA, Arterburn D, Daley MF, Desai J, Fitzpatrick SL, et al. Racial/Ethnic Disparities in the Prevalence of Diabetes and Prediabetes by BMI: Patient Outcomes Research To Advance Learning (PORTAL) Multisite Cohort of Adults in the U.S. Diabetes Care. 2019 Dec 1;42(12):2211–9.
46. Kowall B, Rathmann W, Kuss O, Herder C, Roden M, Stang A, et al. Reversion from prediabetes to normoglycaemia after weight change in older persons: The KORA F4/FF4 study. Nutrition, Metabolism and Cardiovascular Diseases. 2021 Feb;31(2):429–38.
47. Forouhi NG, Luan J, Hennings S, Wareham NJ. Incidence of Type 2 diabetes in England and its association with baseline impaired fasting glucose: The Ely study 1990–2000. Diabetic Medicine. 2007;24(2):200–7.
48. Diabetes Prevention Program Research Group. 10-year follow-up of diabetes incidence and weight loss in the Diabetes Prevention Program Outcomes Study. The Lancet. 2009 Nov;374(9702):1677–86.
49. Liu X, Wu S, Song Q, Wang X. Reversion From Pre–Diabetes Mellitus to Normoglycemia and Risk of Cardiovascular Disease and All-Cause Mortality in a

Chinese Population: A Prospective Cohort Study. JAHA [Internet]. 2021 Feb 2 [cited 2021 Sep 30];10(3). Available from: https://www.ahajournals.org/doi/10.1161/JAHA.120.019045
50. Vistisen D, Kivimäki M, Perreault L, Hulman A, Witte DR, Brunner EJ, et al. Reversion from prediabetes to normoglycaemia and risk of cardiovascular disease and mortality: the Whitehall II cohort study. Diabetologia. 2019 Aug;62(8):1385–90.
51. for the PREDAPS Study Group, Giráldez-García C, Cea-Soriano L, Albaladejo R, Franch-Nadal J, Mata-Cases M, et al. The heterogeneity of reversion to normoglycemia according to prediabetes type is not explained by lifestyle factors. Sci Rep. 2021 Dec;11(1):9667.
52. Falguera M, Vilanova MB, Alcubierre N, Granado-Casas M, Marsal JR, Miró N, et al. Prevalence of pre-diabetes and undiagnosed diabetes in the Mollerussa prospective observational cohort study in a semi-rural area of Catalonia. BMJ Open. 2020 Jan;10(1):e033332.
53. Fallah-Fini S, Rahmandad H, Huang TT-K, Bures RM, Glass TA. Modeling US Adult Obesity Trends: A System Dynamics Model for Estimating Energy Imbalance Gap. Am J Public Health. 2014 Jul;104(7):1230–9.
54. Flack KD, Siders WA, Johnson L, Roemmich JN. Cross-Validation of Resting Metabolic Rate Prediction Equations. Journal of the Academy of Nutrition and Dietetics. 2016 Sep;116(9):1413–22.
55. Roza AM, Shizgal HM. The Harris Benedict equation reevaluated: resting energy requirements and the body cell mass. The American Journal of Clinical Nutrition. 1984 Jul 1;40(1):168–82.
56. STEPwise Approach to NCD Risk Factor Surveillance (STEPS) [Internet]. [cited 2021 Oct 1]. Available from: https://www.who.int/teams/noncommunicable-diseases/surveillance/systems-tools/steps
57. Craig CL, Marshall AL, Sj??Str??M M, Bauman AE, Booth ML, Ainsworth BE, et al. International Physical Activity Questionnaire: 12-Country Reliability and Validity: Medicine & Science in Sports & Exercise. 2003 Aug;35(8):1381–95.
58. Wilks R.J. Jamaica Health and Lifestyle Survey I. Kingston, Jamaica: Epidemiology Research Unit, Tropical Medicine Research Institute, University of the West Indies; 2000.
59. Dugas LR, Bovet P, Forrester TE, Lambert EV, Plange-Rhule J, Durazo-Arvizu RA, et al. Comparisons of intensity-duration patterns of physical activity in the US, Jamaica and 3 African countries. BMC Public Health. 2014 Aug 27;14:882.
60. Bull FC, Maslin TS, Armstrong T. Global Physical Activity Questionnaire (GPAQ): Nine Country Reliability and Validity Study. Journal of Physical Activity and Health. 2009 Nov;6(6):790–804.
61. Ng SW, Popkin BM. Time use and physical activity: a shift away from movement across the globe. Obes Rev. 2012 Aug;13(8):659–80.
62. Götschi T, Tainio M, Maizlish N, Schwanen T, Goodman A, Woodcock J. Contrasts in active transport behaviour across four countries: how do they translate into public health benefits? Prev Med. 2015 May;74:42–8.
63. Howitt C, Brage S, Hambleton IR, Westgate K, Samuels TA, Rose AM, et al. A cross-sectional study of physical activity and sedentary behaviours in a Caribbean population: combining objective and questionnaire data to guide future interventions. BMC Public Health. 2016 Oct 1;16(1):1036.
64. Ogilvie D, Foster CE, Rothnie H, Cavill N, Hamilton V, Fitzsimons CF, et al. Interventions to promote walking: systematic review. BMJ. 2007 Jun 7;334(7605):1204.
65. Xiao C, Goryakin Y, Cecchini M. Physical Activity Levels and New Public Transit: A Systematic Review and Meta-analysis. American Journal of Preventive Medicine. 2019 Mar 1;56(3):464–73.
66. Abioye AI, Hajifathalian K, Danaei G. Do mass media campaigns improve physical activity? a systematic review and meta-analysis. Arch Public Health. 2013 Dec;71(1):20.

67. Food and Agriculture Organization of the United Nations, United Nations University, World Health Organization, editors. Human energy requirements: report of a Joint FAO/WHO/UNU Expert Consultation: Rome, 17-24 October 2001. Rome: Food and Agricultural Organization of the United Nations; 2004. 96 p. (FAO, food and nutrition technical report series).
68. Ainsworth BE, Haskell WL, Herrmann SD, Meckes N, Bassett DR, Tudor-Locke C, et al. 2011 Compendium of Physical Activities: A Second Update of Codes and MET Values. Medicine & Science in Sports & Exercise. 2011 Aug;43(8):1575–81.
69. Harms T, Berrigan D, Gershuny J. Daily metabolic expenditures: estimates from US, UK and polish time-use data. BMC Public Health. 2019 Jun 3;19(2):453.
70. Singh GM, Micha R, Khatibzadeh S, Shi P, Lim S, Andrews KG, et al. Global, Regional, and National Consumption of Sugar-Sweetened Beverages, Fruit Juices, and Milk: A Systematic Assessment of Beverage Intake in 187 Countries. PLoS One [Internet]. 2015 Aug 5 [cited 2019 Feb 18];10(8). Available from: https://www.ncbi.nlm.nih.gov/pmc/articles/PMC4526649/
71. Srour B, Fezeu LK, Kesse-Guyot E, Allès B, Debras C, Druesne-Pecollo N, et al. Ultraprocessed Food Consumption and Risk of Type 2 Diabetes Among Participants of the NutriNet-Santé Prospective Cohort. JAMA Intern Med. 2020 Feb 1;180(2):283.
72. Guariguata L, Brown C, Sobers N, Hambleton I, Samuels TA, Unwin N. An updated systematic review and meta-analysis on the social determinants of diabetes and related risk factors in the Caribbean. Revista Panamericana de Salud Pública. 2018;42:1–11.
73. Orcholski L, Luke A, Plange-Rhule J, Bovet P, Forrester TE, Lambert EV, et al. Under-reporting of dietary energy intake in five populations of the African diaspora. Br J Nutr. 2015 Feb 14;113(3):464–72.
74. Jackson M, Walker S, Forrester T, Cruickshank JK, Wilks R. Social and dietary determinants of body mass index of adult Jamaicans of African origin. European Journal of Clinical Nutrition. 2003 Apr;57(4):621–7.
75. Sharma S, Cao X, Harris R, Hennis AJM, Wu S-Y, Leske MC. Assessing dietary patterns in Barbados highlights the need for nutritional intervention to reduce risk of chronic disease. Journal of Human Nutrition and Dietetics. 2008;21(2):150–8.
76. Harris RM, Rose AMC, Forouhi NG, Unwin N. Nutritional adequacy and dietary disparities in an adult Caribbean population of African descent with a high burden of diabetes and cardiovascular disease. Food Sci Nutr. 2020 Mar;8(3):1335–44.
77. Pan American Health Organization. Ultra-processed food and drink products in Latin America: Sales, sources, nutrient profiles, and policy implications. Washington, D.C.: PAHO; 2019.
78. Monteiro CA, Cannon G, Lawrence ML, Pereira Machado P. Ultra-processed foods, diet quality, and health using the NOVA classification system. Rome, Italy: FAO; 2019.
79. Rauber F, Steele EM, Louzada ML da C, Millett C, Monteiro CA, Levy RB. Ultra-processed food consumption and indicators of obesity in the United Kingdom population (2008-2016). Meyre D, editor. PLoS ONE. 2020 May 1;15(5):e0232676.
80. Martins APB, Levy RB, Claro RM, Moubarac JC, Monteiro CA. Participacao crescente de produtos ultraprocessados na dieta brasileira (1987-2009). Rev Saúde Pública. 2013 Aug;47(4):656–65.
81. Marrón-Ponce JA, Sánchez-Pimienta TG, Louzada ML da C, Batis C. Energy contribution of NOVA food groups and sociodemographic determinants of ultra-processed food consumption in the Mexican population. Public Health Nutr. 2018 Jan;21(1):87–93.
82. Martínez Steele E, Raubenheimer D, Simpson SJ, Baraldi LG, Monteiro CA. Ultra-processed foods, protein leverage and energy intake in the USA. Public Health Nutr. 2018 Jan;21(1):114–24.
83. Cediel G, Reyes M, da Costa Louzada ML, Martinez Steele E, Monteiro CA, Corvalán C, et al. Ultra-processed foods and added sugars in the Chilean diet (2010). Public Health Nutr. 2018 Jan;21(1):125–33.
84. Green R, Cornelsen L, Dangour AD, Turner R, Shankar B, Mazzocchi M, et al. The

effect of rising food prices on food consumption: systematic review with meta-regression. BMJ. 2013 Jun 17;346:f3703.
85. Dallongeville J, Dauchet L, de Mouzon O, Réquillart V, Soler L-G. Increasing fruit and vegetable consumption: a cost-effectiveness analysis of public policies. European Journal of Public Health. 2011 Feb;21(1):69–73.
86. Afshin A, Peñalvo JL, Del Gobbo L, Silva J, Michaelson M, O'Flaherty M, et al. The prospective impact of food pricing on improving dietary consumption: A systematic review and meta-analysis. Adams J, editor. PLoS ONE. 2017 Mar 1;12(3):e0172277.
87. Cecchini M, Warin L. Impact of food labelling systems on food choices and eating behaviours: a systematic review and meta-analysis of randomized studies. Obesity Reviews. 2016;17(3):201–10.
88. Cabrera Escobar MA, Veerman JL, Tollman SM, Bertram MY, Hofman KJ. Evidence that a tax on sugar sweetened beverages reduces the obesity rate: a meta-analysis. BMC Public Health. 2013 Dec;13(1):1072.
89. Stein M. Large Sample Properties of Simulations Using Latin Hypercube Sampling. Technometrics. 1987 May;29(2):143–51.
90. Kaiser KA, Brown AW, Brown MMB, Shikany JM, Mattes RD, Allison DB. Increased fruit and vegetable intake has no discernible effect on weight loss: a systematic review and meta-analysis. Am J Clin Nutr. 2014 Aug 1;100(2):567–76.
91. Ledoux TA, Hingle MD, Baranowski T. Relationship of fruit and vegetable intake with adiposity: a systematic review. Obesity Reviews. 2011 May 1;12(5):e143–50.
92. Wahlich C, Chaudhry UAR, Fortescue R, Cook DG, Hirani S, Knightly R, et al. Effectiveness of adult community-based physical activity interventions with objective physical activity measurements and long-term follow-up: a systematic review and meta-analysis. BMJ Open. 2020 Apr;10(5):e034541.
93. Pazin J, Garcia LMT, Florindo AA, Peres MA, Guimarães AC de A, Borgatto AF, et al. Effects of a new walking and cycling route on leisure-time physical activity of Brazilian adults: A longitudinal quasi-experiment. Health & Place. 2016 May;39:18–25.
94. Prince SA, Saunders TJ, Gresty K, Reid RD. A comparison of the effectiveness of physical activity and sedentary behaviour interventions in reducing sedentary time in adults: a systematic review and meta-analysis of controlled trials. Obes Rev. 2014 Nov;15(11):905–19.
95. World Health Organization. Global action plan for the prevention and control of noncommunicable diseases 2013-2020 [Internet]. Geneva, Switzerland; 2013 [cited 2016 Mar 11]. Available from: http://www.who.int/nmh/events/ncd_action_plan/en/
96. Shangguan S, Afshin A, Shulkin M, Ma W, Marsden D, Smith J, et al. A Meta-Analysis of Food Labeling Effects on Consumer Diet Behaviors and Industry Practices. American Journal of Preventive Medicine. 2019 Feb;56(2):300–14.
97. Harris RM, Rose AMC, Soares-Wynter S, Unwin N. Ultra-processed food consumption in Barbados: evidence from a nationally representative, cross-sectional study. J Nutr Sci. 2021;10:e29.
