# Supplementary material for: Exploring ways to respond to rising obesity and diabetes in the Caribbean using a system dynamics model
Source: PLOS Glob Public Health. 2022 May 19;2(5):e0000436. doi: 10.1371/journal.pgph.0000436 (PMC10021196; doi:10.1371/journal.pgph.0000436)
Supplement: Attachment — Submitted filename: RESPONSE_TO_REVIEWRS_PLOSGH_Guariguata.docx [file pgph.0000436.s002.docx]

Please use the space provided to explain your answers to the questions above. You may also include additional comments for the author, including concerns about dual publication, research ethics, or publication ethics. (Please upload your review as an attachment if it exceeds 20,000 characters)

Reviewer #1: This is an impressively comprehensive modeling exercise to assess the feasibility of achieving global targets for diabetes prevalence and mortality, and more broadly to assess likely trends and intervention impacts in Jamaica over the next 30 years. I recommend acceptance. One element that you may want to consider in the introductory remarks or discussion is the social determinants of health (particularly poverty), as well as the specificity of Jamaica's history, and the impacts these may have on current diabetes patterns. Diabetes has a strong relationship to poverty, and both diabetes and poverty track with formerly colonized and enslaved status in the Americas. While Britain paid slave owners for the inconvenience of abolition in 1834, it has not paid reparations to the Jamaican descendants of enslaved people - worth 7.6 billion pounds today if they were to get the same deal as the slave owners. Given the enormous investments in upstream and downstream programs necessary to level off diabetes prevalence, this British responsibility could prove very relevant.

Response: Thank you for your comments. The historical foundations of the NCD epidemic are an important discussion point in the Caribbean and worth considering. I have added to the discussion a reference to the CARICOM Ten Point Plan for Reparatory Justice (lines 765-770) and the overall lack of funding for NCDs on the global health agenda.

Findings from the JHLS II did not support differences in fasting blood glucose by socioeconomic status but I did include findings from a systematic review that shows that diabetes is higher among lower education levels, which is suggestive of a need for targeted interventions to the poorest. I have added that in (lines 591-594).

Overall, this is excellent, thorough modeling and the paper is well-written.

Reviewer #2: This manuscript presents the results of a modeling exercise in Jamaica to assess trends and policy strategies to address obesity and diabetes in Jamaica. This study builds upon a previously published systems dynamic model with a high level of adaptation to Jamaica. The authors' top-line finding is that targets put forth in the WHO Global Action Plan are totally unrealistic. They also suggest specific measures both upstream and downstream which are likely to make the biggest difference in the future levels of obesity and diabetes in Jamaica. Overall, I found this study to be methodologically rigorous and well-conducted, and I think it should be published in PLOS Global Public Health.

Major comments:

- What I liked most about this study was how it used a well-known modeling platform and then did a lot of formative work to tailor the models to Jamaica and also to incorporate the best-possible input data into the model. It is very impressive.

- I am wondering if the authors might include a brief summary of how the model works beyond the model figures. I am not a quantitative modeler and am unfamiliar with systems modeling. But I am a reasonably average consumer of quantitative modeling papers. I found it hard to understand how the simulation was being performed, whether it was a Markov (assume not), whether uncertainty was incorporated. Are these based on regressions? Pardon my ignorance but a bit more statistical detail for a non-modeler would be useful in the "model implementation" section, rather than referring to the software used. Forgive me if I missed it, but I did not these details in the appendix.

LG Comments: I have added a brief explanation in the methods for the mathematical basis of the modelling (lines 112-113).

- I am confused if the model reports diabetes-related deaths and whether this is part of the present manuscript. I note that death is included in the model figure and is part of the original AJPH paper using this model. For example, the paragraph at the top of page 17 has quite a bit of language in the results section describing the model's impact on death, but it is not clear to me if this is referring to the model results or making an interpretation based on the model assumptions in Table 2. I would ask the authors to either present the death estimates, if they have them, or to clarify if estimated deaths will be reported in a separate publication.

Response: Thank you for raising an important point. The mortality estimates in the model are all-cause mortality for the different glycemic groups. The metric mentioned in the paper is all-cause mortality among people with diabetes. It is difficult to establish diabetes-related deaths in general because many CVD or other deaths may have had diabetes as the underlying cause. Where deaths are mentioned, I’ve clarified that it is all-cause mortality in people with diabetes. (Lines 338, 342,464).

Minor comments:

- Might the authors consider reducing the number of abbreviations to enhance clarity and readability?

Response: Thank you. I’ve removed abbreviations that were only used one or two times.

- I recommend resizing the axes of Figure 2; the vertical orientation makes it a bit difficult to interpret.

Response: I’ve reconfigured the figures to hopefully make them more readable and easier to interpret.

- Thank you for making the code publicly available.

- Some text in results may be better suited for the methods section either in the full text or appendix. (For example line 311: "Scenarios for obesity reduction do not include any downstream interventions like bariatric surgery because there is no evidence that these have a measurable impact at the population level.")

Response: The comment has been noted. This has been moved to the methods section (lines 222-224).

- I found the structure of the results section quite difficult to follow. Do the italics refer to both headings and also subheadings? It would be helpful to more clearly differentiate which text refers to which heading level and to keep more consistent labeling.

Response: I’ve combined some of the subheadings in the results and discussion sections to make them more readable and easier to interpret.

- Line 406 referencing diabetes reversal, I would suggest referencing the results of the DiRECT study to strengthen this paragraph

Response: Thank you. I’ve added a citation for the DiRECT trial. (line 435).

Lean MEJ, Leslie WS, Barnes AC, et al. Primary care-led weight management for remission of type 2 diabetes (DiRECT): an open-label, cluster-randomised trial. The Lancet 2018; 391(10120): 541-51.

Lean MEJ, Leslie WS, Barnes AC, et al. Durability of a primary care-led weight-management intervention for remission of type 2 diabetes: 2-year results of the DiRECT open-label, cluster-randomised trial. The Lancet Diabetes & Endocrinology 2019; 7(5): 344-55.

- Lines 414-418, these are very nice and well-thought-out points about monitoring and targets at the national level. Are there any examples of countries that have done this successfully that you could reference? Might also be useful to consider how the new draft WHO report on global diabetes targets fits into the paradigm you propose:

<https://www.who.int/publications/m/item/improving-health-outcomes-of-people-with-diabetes-mellitus>

Response: The Global Obesity Atlas released their latest edition after the initial submission of the manuscript. It finds that no country is on track to achieve targets for obesity. I have added this reference to the discussion to support that point.

I have included a reference for the monitoring targets set for the Global Diabetes Compact, as per your suggestion (line 441-442) and have also added a mention on progress to the WHO Global Monitoring Framework for NCDs (lines 444-462).

- This paper does an excellent job throughout of citing appropriate literature and positioning its results within the broader landscape of prior studies. At the same time, I wonder if there is a role for simplifying or abbreviating some of the elements to enhance readability. For example, in my estimation, the discussion is approximately 3,000 words, and the limitations section is approximately 1,000 words itself.

Response: Thank you. I have reviewed the discussion in detail and removed parts of it to reduce the length. I have done the same for the limitations.
